# The Prevalence and Characteristics of E-Cigarette Users in the U.S.

**DOI:** 10.3390/ijerph14101200

**Published:** 2017-10-11

**Authors:** David T. Levy, Zhe Yuan, Yameng Li

**Affiliations:** Lombardi Comprehensive Cancer Center, Georgetown University, Washington, DC 20007, USA; zy70@georgetown.edu (Z.Y.); yl954@georgetown.edu (Y.L.)

**Keywords:** electronic cigarettes, e-cigarettes, prevalence, demographics, public policy

## Abstract

Studies have examined the characteristics of individuals who use e-cigarettes, including sociodemographic and smoking characteristics, and the relationship of e-cigarette use to tobacco control policies. While most studies consider a subset of these characteristics with weak measures of regular e-cigarette use, this study uses a large, recent U.S. survey to simultaneously consider the association of each of these factors with different use measures. Data from the May 2014 Tobacco Use Supplement-Current Population Survey is supplemented with information on tobacco control policies. The prevalence of ever, current (at least 1 of the last 30 days), and regular (at least 20 of the last 30 days) e-cigarette use were 7.7%, 2.1% and 0.9%, implying that 27.0% of ever users were current users of which 45.3% were regular users. E-cigarette use varied by socio-demographic characteristics and by smoking status, and depended on the measure of use adopted. However, regardless of measures, e-cigarette use was higher among those smokers who smoked more cigarettes. The association with policies was generally weak, but we found more regular use by smokers in low tax and low tobacco control spending states. The results indicate that the user characteristics differ depending on the e-cigarette use measure. The measure of use should be carefully considered in analyzing how e-cigarette use affects cigarette use.

## 1. Introduction

While conventional cigarette smoking in the U.S. has fallen to an all-time low [1], the use of alternative nicotine delivery products has increased [2,3]. In particular, there has been a large increase in e-cigarette use, especially among the youth and young adults [4,5,6]. With the growth in e-cigarette use, attention has focused on the characteristics of e-cigarette users. Studies have considered the socio-demographic and socio-economic characteristics of individuals, the use of other tobacco products, especially cigarettes, and the states or regions with heaviest use.

Focusing on the U.S., five recent studies [4,5,7,8,9] employed an online sample with one also using random digit dialing [5] to examine individual characteristics of ever and current e-cigarette users in the U.S. Current use was defined as at least one of the last 30 days in all studies. The studies generally found higher use among those at younger ages, males, Whites, current compared to former smokers, former compared to never smokers, and users of non-combustible tobacco compared to non-users. Results for income and education levels were more mixed. Another online survey [10] focused on current and former cigarette smokers, but also considered established e-cigarette use defined as at least 50 times lifetime as well as ever and current use. They obtained similar results to other studies, but the role of younger age diminished in going from ever to current and more established use. In addition, although daily smokers were significantly more likely to report ever use than some day and former smokers, this pattern reversed for more established use, with former smokers being three times more likely than daily smokers to be established users. A study using the 2014 National Health Interview Study (NHIS) also found the highest rates of daily e-cigarette use among recent quitters [11]. A 2014 NHIS study considering only workers [12] found lower current e-cigarette use among former smokers (not distinguished by years quit) and never smokers than current smokers.

Studies have also considered the characteristics of smokers or recent quitters who use e-cigarettes. Studies [8,9,13,14,15,16,17] have generally found that those who use e-cigarettes smoked more cigarettes per day, made more quit attempts, and had higher intention to quit.

Several studies have focused on the association of e-cigarette use with tobacco control policies. Huang et al. [8] found that the odds of ever use was 20% lower (*p* < 0.10) among individuals living in states with comprehensive smoking bans. They did not find cigarette price associated with e-cigarette use, but another study found the highest ever use in states with high cigarette prices [10]. In a separate study, Huang et al. [18] did not find a consistent relationship between e-cigarette use and the price of conventional cigarettes, but, like other demand studies [18,19,20], found e-cigarette use responsive to e-cigarette prices.

While a large literature considers the factors associated with e-cigarette use, individual studies usually consider only a subset of the factors. Unlike previous studies, we simultaneously consider the role of a broad array of individual characteristics, tobacco user characteristics, and state policies. In addition, we employ the large scale, nationally representative May 2014 Tobacco Use Supplement (TUS) of the Current Population Survey (CPS) for the United States. We consider three different measures of e-cigarette use: ever use, at least one day, and at least 20 days use in the last 30 days. Unlike previous studies, we focus on at least 20 days use as a measure of regular use.

## 2. Methods

### 2.1. Primary Data

The TUS-CPS is a repeated cross sectional study, collected in July 2014, January 2015, and May 2015, each includes about 54,000 households containing over 150,000 individuals and is available online [21]. The TUS is a special supplement to the CPS, and is designed to ask extensive questions on tobacco use. A probability sample employs stratified clusters of households drawn from an initial sampling frame that covers the civilian, non-institutionalized population ages 18 and older. About two-thirds of respondents completed the questionnaire by telephone and responses for the remaining third were obtained through in-person interviews. Self-responders (*n* = 163,920), which were over 35% of respondents for each of the three months, were eligible to answer the more detailed tobacco-use questions.

### 2.2. E-Cigarette and Smokeless Tobacco Use

Respondents to the TUS-CPS were first asked whether they had used an e-cigarette even once. Those who had ever used were asked if they now use e-cigarettes every day, some days, or not at all, and those who used e-cigarette some days were asked “how many of the past 30 days did you use an e-cigarette”. We defined e-cigarette use by three measures: ever use as having used an e-cigarette even once, current use as having used e-cigarettes at least one day in the last 30 days, and regular use as having used 20 or more days in last month. Respondents with missing answers to any of the three questions (2560 out of 163,920) were omitted leaving a population of 161,360. With the same questions asked about smokeless tobacco (SLT) use, we created variables for ever, current, and regular SLT use in the last month. Respondents with missing answer to any of those SLT use questions were also omitted (*n* = 238).

### 2.3. Cigarette Smoking Variables

Respondents to the TUS-CPS were first asked whether they had smoked at least 100 cigarettes in their lifetime, and then were asked whether they currently smoked every day, some days, or not at all (leaving 160,825 valid responses to these two questions). A current smoker was defined as someone who had smoked at least 100 cigarettes and who was smoking some days or every day at the time of the survey. Former smokers included individuals who had smoked at least 100 cigarettes and were not smoking at the time of the survey. They were asked how long ago they quit, and we distinguished ≤1 year, >1–3 years, and more than 3 years quit. Former smokers with missing quit time and former smokers who quit less than one year and stated no smoking 12 months ago (*n* = 1086) were omitted.

Current smokers were asked whether they smoked every day or some days, and the number of smoking days in last month for someday smokers. Unknown responses were treated as missing values (*n* = 356) and were omitted. They were also asked the average number of cigarettes they smoked per day (CPD). We weighted their average number of CPD by the number of days smoked in the previous 30 days for some day smokers. We also classified current smokers into four groups by CPD < 5, 5–14, 15–24, and 25 or more. Missing values (*n* = 600) related to CPD were omitted from the total population. Cigarette dependency was also measured by time until first cigarette smoked upon awakening on the days that they smoked, distinguished by less than 5 min, 5–30 min, and more than 30 min. Missing values (*n* = 1243) were omitted. We used current smokers’ intensity (frequency and dependency) when analysing the current population but we used the intensity (12 months ago) when analysing the 12 months ago smoker population (including smokers of at least one year and new quitters). Since the time until first cigarette 12 months ago was not asked for current smokers, we used current practice for smokers and practice 12 months ago for recent quitters. Smokers 12 months ago were omitted from the sample if any of their past practices were unknown and could not be replaced by current practice.

### 2.4. Socio-Demographic Variables

Using socio-demographic information included in the CPS data, the sample was distinguished by sex, age (18–24, 25–34, 35–44, 45–64, 65+), and racial/ethnic groups (White, Black or African American, Asian only, and other races). A separate variable was created to distinguish Hispanics. Educational levels were classified into four groups (less than high school, high school graduate, associate degree, and college graduate and above). Four income levels (less than $20,000, $20,000–$39,999, $40,000–$74,999, and $75,000 or more) were distinguished. We also distinguished marital status by three types (never married, married-spouse present, married-spouse absent or widowed/divorced/separated), urban-rural residency, and employment status (employed and others who are not in labour market or without response).

### 2.5. Policy Variables

Cigarette taxes and tobacco control spending in each state were obtained from the CDC StateSystem [22]. Using the state tax in Jan 2015 and smoking control spending in 2015, we ranked each state from lowest quarter to the highest quarter for both variables. One state, Minnesota, had imposed an e-cigarette tax by the time of survey [22] and was distinguished. Since smoke-free worksites have been associated with reduced smoking [23], we included an indicator for smoke-free worksites by state law, obtained from www.tobacconomics.org. By July 2014, three states, North Dakota, New Jersey, and Utah, had imposed an e-cigarette-free worksites ban. Since the impact of smoke-free worksites is less likely to have any effect if the individual works outdoors, an indicator was created with information from the TUS-CPS regarding whether the individual worked indoors but not at home.

### 2.6. Time and Region Variation

Separate indicators were also included for three survey months and nine regions of residency (East North Central, East South Central, Middle Atlantic, Mountain, New England, Pacific, South Atlantic, West North Central, West South Central) [24].

### 2.7. Statistical Methods

Using the FREQ procedure of SAS (version 9.4, SAS Institute Inc., Cary, NC, USA), unweighted frequencies were calculated separately for the three e-cigarette use measures. A chi-square analysis of within-category differences was conducted for each of the socio-demographic factors, tobacco control policies, smoking intensity and the measures of SLT use status. Using the SURVEYLOGISTIC procedure of SAS (version 9.4), weighted (using self-response weights provided by TUS-CPS) logistic regression models were estimated using the Fisher scoring method as the optimization technique. Separate multivariate logistic regression equations were estimated for the total population and its subsets by smoking status for each of the three measures to isolate the contribution of each variable. The smoker 12 months ago population (*n* = 21,300) excluded non-smokers 12 months ago and included former smokers < 1 year. Current never smokers and current smokers who stated they did not smoke at all one year ago were considered as part of the never smoking population (*n* = 106,138). Those former smokers who quit >1–3 years (*n* = 2892) and those who quit more than 3 years ago (*n* = 27,578) were separated into two different subpopulations. For each of the subpopulations, we first estimated separate equations for males and females, and found that the results by sex were similar, based on the coefficients for individual variables from the female equations being contained within the confidence interval of the corresponding coefficient from the male equations. Consequently, we pooled the male and female equations and allowed for interactions by for the variables whose coefficients were found to differ (e.g., ages 18–34 and former smokers 1–3 years).

## 3. Results

### 3.1. Frequency of E-Cigarette Use

Table 1 presents e-cigarette use rates by individual characteristics, state policies and cigarette and SLT use characteristics. We focus on the results for the regular use measure. For comparability, we discuss the percentage of current users among ever users as the current-ever ratio and percentage of regular among current users as regular-current ratio.

#### 3.1.1. Total Population

As shown in Table 1, the prevalence of ever, current and regular e-cigarette use for the total population was 7.7%, 2.1% and 0.9%, implying that 27.0% of ever users were current users of which 45.3% were regular users. The current-ever and regular-current ratios were close by most characteristics, with exceptions as noted below.

Regular e-cigarette use was 1.1% among males and 0.8% among females, and for both sexes was 1.3% among age groups 18–24 and 25–34, decreasing to 1.2%, 1.0%, and 0.4% among age groups 35–44, 45–64, and ages 65+. The current-ever ratio was lower among those at younger than at older ages. Regular e-cigarette use was higher among Whites and Other races than Blacks and Asians. Regular e-cigarette use was higher among those with an associate or a high school degree than among those with less than 12 years or who had a college or higher degree, a relatively small percentage (21.9%) of college graduates who tried e-cigarettes as current users. Regular e-cigarette use was lower at high incomes, among married with spouse present, and among those employed. 

Regarding smoking status, regular e-cigarette use was highest among former smokers who quit in the last year (12.7%), followed by former smokers who quit >1–3 years (7.4%), current smokers (3.6%), former smokers who quit more than 3 years (0.3%) and never smokers (0.1%). Former smokers who were quit less than one year had the highest current-ever (37.6%) and regular-current (77.8%) ratios, followed closely by those who quit >1–3 years (32.5% and 75.4%), followed by former smokers >3 years (17.5% and 59.1%) and current smokers (28.1% and 34.0%). Never smokers had the lowest ratio of current-ever use (14.3%) and regular-current use (31.8%). Among current smokers, regular e-cigarette use with CPD 1–4 is largest (5.4%) compared with other CPD categories. It also has highest current-ever ratio (33.8%) and regular-current ratio (46.1%). Those who smoked their first cigarette 5–30 min upon awakening have the lowest regular use rate and the lowest regular to current rate. Regular e-cigarette use was also lower among everyday than someday smokers. Regular e-cigarette use was similar between regular and non-regular SLT users, although ever and current e-cigarette use was higher among SLT than non SLT users.

Regular e-cigarette use was higher in the states with lower than with higher cigarette taxes, and in low than in high tobacco control spending states. Regular e-cigarette use was lower in states with the highest cigarette worksite ban than other states, but the prevalence of ever e-cigarette use was similar across these states. Regular e-cigarette use was similar in states with an e-cigarette worksite ban and states with e-cigarette tax to other states, but ever e-cigarette use was lower in states with highest e-cigarette worksite ban.

#### 3.1.2. E-Cigarette Use by Sex and Smoking Status

When we divided the sample by sex (not shown—see Supplementary Table S1e,f), regular e-cigarette use was higher among males than females (especially for those age 18–24 and smokers who quit less than one year).

When we divided the sample by smoking category (smokers one year ago, former smokers who quit >1–3 years, and former smokers >3 years and never smokers one year ago (not shown—see Supplementary Table S1a–d), former smokers who quit >1–3 years had high rates of regular use and current use among those ages 35–64, whereas never smokers, smokers one year ago, and former smokers who quit >3 years had declining e-cigarette regular and current use with age. Different patterns of use were also found by income: regular use increased with income for smokers one year ago and former smokers >1–3 years, unlike former smokers >3 years and never smokers one year ago.

### 3.2. Logistic Regression Analysis

Table 2, Table 3, Table 4, Table 5 and Table 6 present the logistic regression results first for the total population and then for the smokers 12 months ago, former (>1–3 and >3 years), and never smokers 12 months ago populations based on their smoking status one year ago. In each table, separate logistic regression equations were estimated with ever, current and regular e-cigarette use as the dependent variables.

#### 3.2.1. Total Population

Table 2 presents the logistic equations for the sample that included all individuals regardless of smoking status. The odds of regular e-cigarette use was higher among males than females at age 18–34, among those with a high school and associate degree, and for those living in metropolitan areas, and was lower among age 45–64 and 65+, among Blacks and Asians compared to Whites, and among Hispanic compared to non-Hispanic. Compared to the equation with the ever and current use measures as dependent variables, the differentials by age, sex, and marital status were similar for the regular use measure. Among the policy variables, the only consistent finding that emerged was lower e-cigarette use in states at the highest quarter of tobacco control spending.

The odds of regular use were about 140 times greater for former smokers who quit in the last year than never smokers. However, their odds ratio fell to 56 for current use and to 41 for ever use, suggesting that the likelihood of becoming a former smoker in the last year is more closely related to regular use. Similarly, for those who quit >1–3 years, the odds ratio for regular use was near 80 (males showed higher odds than females only by current use as indicated by the interaction term), again indicating that recent quitting is related to regular use. The odds of regular use among former smokers >3 years was 4 times higher than never smokers. For all e-cigarette use measures, the odds ratio was around 20–40 for current smokers, indicating substantially higher use for smokers compared to never smokers.

Consistently higher odds across measures was observed for some day smokers than every day smokers and smokers who smoked the first cigarette <5 min upon awakening than those who smoked the first cigarette >30 min upon awakening; however significantly lower regular use odds among those who smoked 15–24 and 25+ CPD reversed to significantly higher odds for the ever use measure compared to 1–4 CPD smokers.

#### 3.2.2. By Smoking Status

Among those who were smokers one year ago (Table 3), the odds of regular e-cigarette use were less among those age 65 and above, Blacks compared to Whites, and Hispanics compared to non-Hispanics and greater among those with high school and associate degrees and among males at age 18–34 than females the same age. Compared to the ever and current use measures, the differentials by age became less prominent with regular use. High tobacco control spending states was associated with less regular, current and ever e-cigarette use in comparison to the states with very low spending, but no relationship was observed for other policy variables. For those who were smokers one year ago, the odds of regular use by former smokers ≤1 year was almost 4 times greater than for smokers, declining to 1.5 for current use and 1.3 for ever use. The odds of regular and current use by former smokers ≤1 year increased 1.5 times for males compared to females (as indicated by the interaction term). Compared to 1–4 CPD, the odds ratio of regular use increased from 1.5 for 5–14 CPD, to 1.9 for 15–24 CPD and to 2.6 for 25+ CPD, with similar increases for ever and current use. The odds ratios of current and ever use were 1.3–1.5 for someday smokers than every day smokers, but not significant for regular use. The odds ratios of smoking ≤5 and 5–30 min upon awakening compared to >30 min upon awakening were both 1.2 with ever use, but not significant with current use and regular use. The odds of ever and current e-cigarette use was 5–6 times greater among SLT users compared to non-SLT users and male SLT current users had lower odds than females by current use measure, but not related for regular use.

Among former smokers >1–3 years (Table 4), lower odds of regular use were obtained for males, age 18–24, Hispanics and non-metropolitan residents and higher odds of regular use among males than females at all income levels, with different patterns shown for other individual characteristics for the ever and current use measures. Among policies, no relationship was found with regular e-cigarette use, but the odds of ever use was higher in high cigarette tax than low cigarette tax states. The odds of ever e-cigarette use was higher among those who ever used SLT than non-users, but this relationship weakened and appeared to reverse itself for current and regular use.

Among former smokers who quit more than 3 years (Table 5), the odds of e-cigarette use decreased with age for all use measures. While some differences were found for marital status and metropolitan residence for ever use, they weakened for regular use. Odds of ever use was higher among those who ever used SLT, but the ability to detect differences for regular use was limited by the lack of individual cases.

For never smokers one year ago (Table 6), the odds of regular e-cigarette use were higher for unmarried and married-spouse absent or widowed/separated compared to married-spouse present and lower for older age groups, Blacks and other races compared to Whites, and Hispanics compared to non-Hispanics. Relationships found for ever and current use for younger age groups, income, education and metropolitan residency weakened for regular use. Those who transitioned to smoking in the past year had substantially higher use rates for all measures. Compared to low tax states, high cigarette tax states had higher ever use, while no relationship was found with regular use. Med-low tobacco control spending states had higher odds than low spending states by all measures. States with either cigarette or e-cigarette worksite bans or the three taxing e-cigarettes (North Dakota, New Jersey, and Utah) had lower ever use odds. Ever e-cigarette use was higher with SLT use, but became insignificant for current and regular use.

## 4. Discussion

We found different patterns of ever, current (at least 1 of the last 30 days) and regular (at least 20 of the last 30 days) use associated with individual characteristics, smoking behaviors and state tobacco control policies. As in previous studies [4,5,7,8,9,10,11], high rates of ever use translate into low current use rates, only about 25%, in our study. Furthermore, we found that less than half of current use translates into regular use. We found that some of the tendencies found for ever and current use, such as higher e-cigarette use rates among the young became less prominent for regular users.

Like other U.S. studies [4,5,7,8,9,10,11,12], there were substantial differences by smoking status. We found substantially higher use by smokers and short- and mid-term former smokers for all measures. However, recent quitters had particularly high rates of regular use compared to smokers, long-term former smokers and never smokers. The current-ever (14.3%) and the regular-current (31.8%) ratios were substantially lower among never smokers than current and former smokers who had quit more than three years ago, while these ratios were substantially higher among recent (within the last 3 years) former smokers than others. These findings indicate that ever and current use is likely to overstate more regular e-cigarette use by never smokers and at the same time understate current and particularly regular use by those who have quit smoking.

We found that e-cigarette ever use increased with the number of cigarettes smoked per day for those who were smokers one year ago. However, that relationship reverses, i.e., current smokers who smoke more CPD are less likely to be regular users, suggesting that e-cigarettes are particularly effective in enabling heavy smokers to quit. This outcome is consistent with recent findings using the same sample to examine cessation, finding that regular e-cigarette use was associated with past year cessation [25]. We also found that those who smoked soon after awakening were more likely to have ever used e-cigarettes, but that relationship weakened for current and regular use. Regular use of e-cigarettes may reduce cigarette dependency or those more dependent may be less likely to progress to regular use by this measure of dependency. Someday smoking was related to e-cigarette use. These smokers may be transitioning from cigarette use to stay dual smokers. Tobacco control policies may also be needed to stress the importance of quitting rather than dual use for those who are most addicted. While differences were observed among ever and current users in the use of smokeless tobacco, these differences diminished for regular users.

Our findings suggest that media or other policies that encourage regular rather than occasional e-cigarette use as a replacement for cigarettes may promote cessation. However, the importance of eventual quitting cigarette use rather than dual use should be stressed.

Unlike many of the previous e-cigarette prevalence studies, we incorporated the relationship of e-cigarette use to tobacco control policies at the state level. The associations were generally weak, but e-cigarettes use among smokers was less in states with high levels of tobacco control spending, which may reflect smokers being discouraged from switching to e-cigarettes or having already switched to e-cigarettes in states with stronger tobacco control policies. These relationships were not observed for former smokers >1 year or never smokers. Smoke-free air laws (three states) may discourage ever use, but not current or regular use, and taxes for e-cigarettes (one state) were not found to reduce their use. Further research is needed on the role of policies, especially as those policies are in place for longer periods of time.

We focused on use in at least 20 of the last 30 days as our measure of regular use. However, we also considered at least 5 of the last 30 days and at least 10 of the last 30 days use (see Supplementary Tables S1a–f, S2a,b, S3a,b, S4a,b, and S5a,b). In general, we found that e-cigarette use decreased with the frequency of use overall and for each of the variables considered. Decreases in use appeared to be continuous as we went from less frequent to more frequent use. Nevertheless, our measure of regular use only takes into account use in the last month and does not incorporate use over more prolonged periods of time, nor does it incorporate the intensity of daily use, i.e., the number of e-cigarette use occasions per day. Future study should consider these other characteristics of regular use.

Our study focused on the U.S., since use patterns are likely to vary for other countries with different populations and regulatory policies toward cigarettes and e-cigarettes. However, a recent meta-analysis including studies from many countries also found ever and current e-cigarette use highest among smokers and lowest among never smokers [26]. Studies for European countries [27,28,29,30] and Canada [31] have also found more use at younger ages and higher income and educational levels, although one study found greater use at higher ages [32].

While our study applied a large, representative sample, the data is cross-sectional, and is thus subject to recall bias and does not indicate potential transitions over time. The extent of transition by current users’ different levels of e-cigarette use and to alternative forms of nicotine delivery products were not considered. In developing appropriate measures of use, dual and exclusive e-cigarette use may require different definitions in gauging transitions to long-term use [33]. Longitudinal studies will be needed that distinguish not only by age, but also by cohort. For example, an individual experiencing first generation e-cigarettes for the first time six years ago is likely to have a different experience than those experiencing recent generation e-cigarettes, and their transitions may depend on the policies regarding cigarette and e-cigarette use that were in place at particular ages.

The results also depend on the variables included in the equations, and the way that they are measured. Other characteristics, such as tobacco use by one’s peers or parents may be important. While we included state policies, federal, state and local tobacco control policies may be relevant, and their relationship to e-cigarette use may depend on the way that they are measured. 

The public health implications of e-cigarette use depend on the likely use cigarette patterns in the absence of e-cigarettes [33]; exclusive use of e-cigarettes by smokers who would not have otherwise quit cigarette use or who would have otherwise initiated smoking improves population health, whereas smokers who regularly use e-cigarettes rather than quit use or e-cigarette use by those who would not have other initiated cigarette use yields population harm. In order to gauge these effects, it will be important to distinguish which individuals use e-cigarettes, i.e., whether those who use e-cigarettes have the characteristics of those who are likely to have initiated smoking from never smoker or to have quit as a current smoker, and how e-cigarette use affects transitions into and out of other types of tobacco, such as smokeless tobacco and cigarettes.

## 5. Conclusions

Our study indicates that the measure of e-cigarette use which is adopted is likely to be important. We have found important differences in use patterns between never, current and former smokers, and, most important, the patterns of use differed depending on the measure used. In particular, our results indicate that it is essential to distinguish infrequent, experimental use from more established, regular use in analyzing the role of e-cigarette use in the uptake and cessation of cigarette and other tobacco product use [33].

## Figures and Tables

**Table 1:** Ever, current and regular measures of e-cigarette use, total population, CPS-TUS, 2014/5.

Variable	Categories	Sample Size	Ever Use of an E-Cigarette		Current Use (≥1 of Past 30 Days)		Regular Use (≥20 of Past 30 Days)		Ratio (Current/Ever Use)	Ratio (Regular/Current Use)
			Rate	χ2 (*p*)	Rate	χ2 (*p*)	Rate	χ2 (*p*)		
Overall		158,626	7.7%		2.1%		0.9%		27.0%	45.3%
Sex	Male	70,588	8.6%	134.6	2.3%	20.9	1.1%	44.7	26.4%	49.7%
	Female	88,038	7.0%	<0.001	1.9%	<0.001	0.8%	<0.001	27.6%	41.3%
Age	18–24	10,470	14.8%		3.3%		1.3%		22.3%	39.4%
	25–34	25,705	12.8%		3.0%		1.3%		23.3%	44.9%
	35–44	26,191	9.4%		2.6%		1.2%		28.0%	44.6%
	45–64	58,333	7.0%	3357.7	2.1%	544.0	1.0%	204.2	30.0%	46.7%
	65+	37,927	2.3%	<0.001	0.7%	<0.001	0.4%	<0.001	32.5%	49.6%
Race	White	131,464	8.1%		2.2%		1.0%		27.5%	46.1%
	Black	16,248	4.7%		1.1%		0.4%		22.5%	36.6%
	Asian	6388	3.6%	548.9	0.8%	192.2	0.3%	105.3	21.5%	38.8%
	Other Races	4526	12.7%	<0.001	3.4%	<0.001	1.5%	<0.001	27.0%	42.6%
Hispanic	Hispanic	16,724	4.7%	235.1	1.1%	88.5	0.4%	50.8	23.3%	40.0%
	Non-Hispanic	141,902	8.1%	<0.001	2.2%	<0.001	1.0%	<0.001	27.3%	45.6%
Education	Less than 12 years	15,903	8.1%		2.2%		0.9%		27.5%	41.0%
	High school degree	44,417	9.2%		2.6%		1.2%		28.4%	46.9%
	Associate degree	45,940	10.2%	1,422.4	2.9%	540.5	1.3%	256.6	28.1%	45.4%
	College or higher degree	52,366	4.2%	<0.001	0.9%	<0.001	0.4%	<0.001	21.9%	44.5%
Family income	$0–$19,999	27,356	10.2%		2.8%		1.2%		27.8%	43.2%
	$20,000–$39,999	36,380	8.9%		2.6%		1.1%		29.1%	44.6%
	$40,000–$74,999	43,473	7.7%	604.0	2.0%	225.7	0.9%	75.3	25.8%	45.9%
	$75,000 or more	51,417	5.7%	<0.001	1.4%	<0.001	0.7%	<0.001	25.3%	47.9%
Marital status	Never Married	34,492	12.1%		2.8%		1.2%		23.4%	42.0%
	Married Spouse Present	83,335	5.6%	1,484.4	1.6%	235.7	0.7%	85.9	28.2%	46.5%
	Married Spouse Absent or Widowed/Divorced/Separated	40,799	8.4%	<0.001	2.5%	<0.001	1.2%	<0.001	29.9%	46.9%
Employment status	Employed	94,362	8.5%	167.4	2.2%	5.8	1.0%	14.4	25.6%	47.4%
	Not in labor force or unemployed	64,264	6.7%	<0.001	2.0%	0.016	0.8%	0.000	29.7%	42.1%
Metropolitan status	MSA	122,702	7.4%	82.3	2.0%	15.5	0.9%	2.9	27.2%	45.9%
	Non-MSA	35,924	8.9%	<0.001	2.4%	<0.001	1.0%	0.087	26.5%	43.6%
Indoor workers	Yes	64,676	8.3%	48.5	2.1%	1.7	1.0%	3.1	25.9%	46.5%
	No	93,950	7.4%	<0.001	2.1%	0.187	0.9%	0.080	27.9%	44.5%
Current smoking status	Current smoker	20,350	37.7%		10.6%		3.6%		28.1%	34.0%
	Never smokers	104,654	1.4%		0.2%		0.1%		14.3%	31.8%
	Former (<1 year)	3152	43.4%		16.3%		12.7%		37.6%	77.8%
	Former (1–3 years)	2892	30.2%	39,836.2	9.8%	13,251.1	7.4%	8,418.7	32.5%	75.4%
	Former (≥3 years)	27,578	3.1%	<0.001	0.5%	<0.001	0.3%	<0.001	17.5%	59.1%
Smoking frequency	Everyday smokers	16,627	37.8%	1.1	10.1%	19.7	3.1%	65.4	26.1%	45.2%
	Someday smokers	3723	36.9%	0.3	12.6%	<0.001	5.8%	<0.001	34.1%	46.3%
Cigarette per day	1–4	4184	34.4%		11.6%		5.4%		33.8%	46.1%
	5–14	7657	37.4%		10.4%		3.6%		27.8%	35.0%
	15–24	6989	39.4%	32.9	10.1%	7.2	2.6%	57.8	25.5%	26.2%
	25+	1520	40.2%	<0.001	10.9%	0.1	3.0%	<0.001	27.2%	27.1%
First cigarette upon awakening	≤5 min	3501	42.6%		11.5%		4.1%		27.1%	35.1%
	5–30 min	6810	38.7%	64.2	10.6%	4.7	3.2%	6.2	27.4%	30.0%
	>30 min	10,039	35.3%	<0.001	10.2%	0.1	3.7%	0.0	29.0%	36.4%
Smokeless tobacco use ^§^	Yes	12,039	25.3%	5,601.8	4.5%	77.8	0.9%	0.1	-	-
	No	146,587	6.3%	<0.001	2.0%	<0.001	0.9%	0.8	-	-
State level cigarette tax ranking ^†^	Lowest quarter	32,990	8.6%		2.6%		1.2%		30.7%	44.8%
	Med-low quarter	47,024	7.8%		2.1%		1.0%		26.9%	48.0%
	Med-high quarter	40,094	7.7%	71.9	2.0%	80.2	0.8%	37.3	25.9%	41.8%
	Highest quarter	38,518	6.9%	<0.001	1.7%	<0.001	0.8%	<0.001	24.5%	46.3%
Tobacco control spending per capita ^†^	Lowest quarter	40,960	7.8%		2.3%		1.0%		29.7%	44.7%
	Med-low quarter	40,428	8.0%		2.1%		1.0%		26.7%	46.2%
	Med-high quarter	49,506	6.9%	79.9	1.9%	23.9	0.9%	8.4	26.8%	46.3%
	Highest quarter	27,732	8.7%	<0.001	2.1%	<0.001	0.9%	0.038	24.3%	43.6%
State level worksite smoking ban	Highest level	98,752	7.7%	0.0	2.0%	9.6	0.9%	9.5	25.9%	44.4%
	Not highest level	59,874	7.7%	0.843	2.2%	0.002	1.0%	0.002	28.9%	46.7%
State level worksite e-cigarette ban	Highest level	7129	6.7%	11.8	1.8%	3.2	0.8%	2.1	26.9%	43.8%
	Not highest level	151,497	7.8%	0.001	2.1%	0.075	1.0%	0.149	27.0%	45.4%
State level e-cigarette tax	Yes	3003	8.8%	5.1	2.2%	0.2	1.3%	4.8	24.9%	60.6%
	No	155,623	7.7%	0.025	2.1%	0.678	0.9%	0.028	27.1%	45.0%
Census division	East North Central	18,088	8.8%		2.3%		1.0%		26.1%	44.4%
	East South Central	10,926	8.9%		2.7%		1.3%		30.5%	49.3%
	Middle Atlantic	13,226	6.4%		1.6%		0.7%		25.2%	42.0%
	Mountain	18,768	8.7%		2.6%		1.2%		29.6%	46.3%
	New England	14,249	6.3%		1.6%		0.7%		25.2%	43.2%
	Pacific	21,002	6.8%		1.6%		0.7%		23.6%	46.3%
	South Atlantic	29,781	6.7%		1.9%		0.8%		27.9%	44.8%
	West North Central	16,529	9.3%	282.2	2.4%	124.3	1.0%	73.1	25.9%	41.6%
	West South Central	16,057	8.4%	<0.001	2.4%	<0.001	1.2%	<0.001	28.3%	48.8%

^§^ The prevalence of e-cigarette use by three measures are estimated among SLT users categorized by the same measure (SLT ever use: *n* = 12,039; current use: *n* = 2656; and regular use 1933). ^†^ Data for state level cigarette tax ranking are based on the January 2015 level and for per capita tobacco control spending are for the year 2015.

**Table 2:** Odds ratios for ever, current and regular measures of e-cigarette use, CPS-TUS, 2014/15.

Variable	Category	Ever Use of an E-Cigarette		Current Use (≥1 of Past 30 Days)		Regular Use (≥20 of Past 30 Days)	
		OR ^Δ^	95% CI	OR	95% CI	OR	95% CI
**Intercept**		0.01 ***	0.010–0.015	0.00 ***	0.002–0.004	0.00 ***	0.000–0.001
Sex (ref: Female)	Male	0.68 ***	0.634–0.735	0.89 **	0.797–0.992	1.04	0.883–1.224
Age (ref: 35–44)	18–24	1.97 ***	1.727–2.237	1.14	0.928–1.398	0.90	0.653–1.231
	25–34	1.15 ***	1.035–1.272	0.91	0.769–1.072	0.84	0.650–1.075
	45–64	0.70 ***	0.649–0.752	0.79 ***	0.699–0.885	0.84 **	0.712–0.998
	65+	0.34 ***	0.304–0.380	0.47 ***	0.392–0.568	0.51 ***	0.391–0.666
	Age 18–34 * Male	1.79 ***	1.585–2.028	1.46 ***	1.204–1.770	1.54 ***	1.157–2.040
Race (ref: White)	Asian	0.61 ***	0.504–0.745	0.50 ***	0.342–0.732	0.40 ***	0.214–0.756
	Black	0.54 ***	0.484–0.607	0.44 ***	0.360–0.538	0.38 ***	0.273–0.522
	Other races	1.27 ***	1.075–1.493	1.34 **	1.056–1.698	1.05	0.763–1.456
Hispanic (ref: Non-Hispanic)	Hispanic	0.72 ***	0.638–0.804	0.55 ***	0.448–0.668	0.40 ***	0.294–0.536
Education (ref: Less than 12 years)	High school degree	1.25 ***	1.119–1.387	1.23 ***	1.054–1.440	1.39 ***	1.101–1.756
	Associate degree	1.63 ***	1.458–1.815	1.46 ***	1.248–1.714	1.37 **	1.077–1.745
	College or higher degree	1.25 ***	1.107–1.416	0.99	0.811–1.199	0.84	0.624–1.117
Family income (ref: $0–$19,999)	$20,000–$39,999	1.00	0.918–1.098	1.00	0.875–1.140	1.05	0.865–1.268
	$40,000–$74,999	0.98	0.889–1.073	0.89	0.775–1.031	0.94	0.763–1.161
	$75,000 or more	1.07	0.962–1.190	1.04	0.886–1.224	1.09	0.860–1.373
Marital status (ref: Married–Spouse Present)	Never Married	1.27 ***	1.172–1.369	0.99	0.871–1.117	0.92	0.762–1.098
	Married-Spouse Absent or Widowed/Divorced/Separated	1.25 ***	1.169–1.346	1.11 *	0.992–1.238	1.16 *	0.994–1.355
Employment status (ref: Employed)	Not in labor force or unemployed	1.02	0.930–1.110	1.10	0.961–1.268	1.01	0.825–1.232
Metropolitan status	MSA	1.22 ***	1.135–1.316	1.16 **	1.036–1.303	1.15 *	0.977–1.362
Indoor workers (ref: No)	Yes	1.06	0.977–1.148	1.13 *	0.992–1.281	1.09	0.910–1.299
Current smoking status ^§^ (ref: Never Smokers)	Former(<1 year)	40.69 ***	34.987–47.333	55.66 ***	43.846–70.649	137.53 ***	96.2–196.5
	Former (1–3 years)	20.61 ***	18.147–23.404	34.56 ***	27.589–43.302	79.58 ***	56.3–112.4
	Former (1–3 years) * Male	0.90	0.726–1.108	1.36 **	1.049–1.750	1.28	0.949–1.731
	Former (≥3 years)	2.37 ***	2.131–2.643	2.41 ***	1.880–3.077	4.45 ***	3.1–6.5
	Current smokers	21.29 ***	18.006–25.166	34.46 ***	25.905–45.841	39.37 ***	24.5–63.4
	Current * Someday smokers	1.33 ***	1.135–1.562	1.47 ***	1.172–1.842	1.88 ***	1.306–2.716
	Current * 5–14 CPD	1.46 ***	1.249–1.712	1.03	0.824–1.297	0.84	0.578–1.223
	Current * 15–24 CPD	1.59 ***	1.342–1.886	0.94	0.736–1.207	0.51 ***	0.328–0.775
	Current * 25+ CPD	1.91 ***	1.535–2.369	1.14	0.837–1.551	0.56 **	0.331–0.963
	Current * ≤ 5 min first cig	1.28 ***	1.136–1.443	1.25 **	1.052–1.481	1.62 ***	1.207–2.162
	Current * 5–30 min first cig	1.25 ***	1.136–1.372	1.15 **	1.002–1.318	1.18	0.929–1.501
Smokeless tobacco use ^†^ (ref: No)	Yes	6.05 ***	4.921–7.439	4.37 ***	1.947–9.787	1.35	0.144–12.668
	Yes * Male	0.66 ***	0.529–0.832	0.28 ***	0.121–0.667	0.31	0.030–3.098
State level cigarette tax ranking ^‡^ (ref: Lowest quarter)	Med-low quarter	1.07	0.958–1.191	0.96	0.819–1.135	1.13	0.893–1.427
	Med-high quarter	1.14 **	1.022–1.281	0.95	0.803–1.132	0.99	0.775–1.274
	Highest quarter	1.25 ***	1.093–1.439	0.90	0.715–1.120	1.02	0.736–1.419
Tobacco control spending per capita ^‡^ (ref: Lowest quarter)	Med-low quarter	1.04	0.954–1.140	0.98	0.855–1.125	1.11	0.910–1.345
	Med-high quarter	0.95	0.866–1.043	0.91	0.792–1.051	0.96	0.785–1.184
	Highest quarter	0.87 ***	0.778–0.962	0.73 ***	0.623–0.864	0.77 **	0.609–0.978
State level worksite smoking ban	Highest level	0.92 *	0.837–1.001	0.99	0.864–1.143	0.95	0.776–1.158
State level worksite e-cigarette ban	Highest level	0.84 **	0.714–0.996	1.11	0.847–1.440	1.21	0.814–1.796
State level e-cigarette tax	Yes	0.88	0.700–1.115	0.96	0.665–1.392	1.24	0.755–2.041
Survey time (ref: July 2014)	Jan 2015	1.04	0.972–1.116	0.96	0.866–1.071	0.92	0.787–1.070
	May 2015	0.99	0.926–1.067	0.86 ***	0.768–0.958	0.94	0.806–1.103
Census division (ref: Pacific)	East North Central	0.83 ***	0.727–0.957	0.92	0.740–1.144	0.86	0.628–1.187
	East South Central	0.81 ***	0.705–0.933	1.05	0.843–1.308	1.29	0.948–1.755
	Middle Atlantic	0.80 ***	0.692–0.933	0.91	0.714–1.155	0.85	0.594–1.210
	Mountain	1.20 **	1.043–1.373	1.34 ***	1.080–1.668	1.42 **	1.035–1.948
	New England	0.65 ***	0.549–0.762	0.88	0.679–1.145	0.85	0.572–1.264
	South Atlantic	0.85 ***	0.751–0.960	1.05	0.860–1.276	1.17	0.874–1.557
	West North Central	0.88 *	0.763–1.019	1.02	0.812–1.276	1.02	0.731–1.429
	West South Central	0.84 ***	0.730–0.958	1.09	0.881–1.346	1.24	0.910–1.681

**^Δ^** Odds ratio and significance level of *p*-value: “***” < 0.01; 0.01 < “**” < 0.05; 0.05 < “*” ≤ 0.1. **^§^** Interaction terms of smoking frequency (CPD 1–4, 5–14, 15–24, 25+ and someday smokers) and dependency (first cigarette < 5 min, 5–30 min, > 30 min upon awakening) were created against current smokers. ^†^ The smokeless tobacco use measure in each model corresponds to the e-cigarette use measure. ^‡^ Data for state level cigarette tax ranking are based on the January 2015 level and for per capita tobacco control spending are for the year 2015.

**Table 3:** Odds ratios for, ever, current and regular measures of e-cigarette use of smokers 12 months ago, CPS-TUS, 2014/5.

Variable	Category	Ever Use of an E-Cigarette		Current Use (≥1 of Past 30 Days)		Regular Use (≥20 of Past 30 Days)	
		OR ^Δ^	95% CI	OR	95% CI	OR	95% CI
**Intercept**		0.38 ***	0.284–0.505	0.08 ***	0.053–0.128	0.02 ***	0.011–0.044
Sex (ref: Female)	Male	0.57 ***	0.515–0.620	0.74 ***	0.650–0.846	0.84	0.686–1.037
Age (ref: 35–44)	18–24	1.56 ***	1.296–1.865	1.15	0.886–1.481	1.05	0.721–1.539
	25–34	1.11	0.973–1.275	0.98	0.803–1.183	0.89	0.655–1.205
	45–64	0.73 ***	0.663–0.804	0.80 ***	0.701–0.923	0.83 *	0.675–1.008
	65+	0.45 ***	0.392–0.521	0.57 ***	0.462–0.703	0.63 ***	0.465–0.860
	Age 18–34 * Male	1.45 ***	1.237–1.701	1.32 **	1.043–1.660	1.52 **	1.075–2.154
Race (ref: White)	Asian	0.70 **	0.538–0.922	0.60 **	0.369–0.981	0.54	0.241–1.200
	Black	0.65 ***	0.570–0.750	0.56 ***	0.439–0.701	0.51 ***	0.348–0.760
	Other races	1.28 **	1.054–1.554	1.47 ***	1.121–1.939	1.30	0.889–1.913
Hispanic (ref: Non-Hispanic)	Hispanic	0.73 ***	0.625–0.853	0.61 ***	0.476–0.787	0.46 ***	0.315–0.682
Education (ref: Less than 12 years)	High school degree	1.18 ***	1.052–1.321	1.21 **	1.018–1.444	1.38 **	1.053–1.806
	Associate degree	1.51 ***	1.340–1.699	1.49 ***	1.245–1.781	1.45 ***	1.095–1.922
	College degree or higher	1.56 ***	1.347–1.808	1.42 ***	1.130–1.780	1.26	0.885–1.791
Family income (ref: $0-$19,999)	$20,000–$39,999	1.03	0.935–1.143	0.98	0.846–1.136	0.96	0.769–1.199
	$40,000–$74,999	1.02	0.917–1.141	0.89	0.759–1.052	0.90	0.700–1.156
	$75,000 or more	1.20 ***	1.058–1.364	1.02	0.847–1.232	1.08	0.820–1.423
Marital status (ref: Married-Spouse Present)	Never Married	0.94	0.845–1.035	0.87 *	0.753–1.011	0.88	0.703–1.096
	Married-Spouse Absent or Widowed/Divorced/Separated	1.04	0.955–1.138	0.98	0.861–1.109	1.11	0.925–1.338
Employment status (ref: Employed)	Not in labor force or unemployed	1.00	0.899–1.111	1.19 **	1.012–1.389	1.02	0.807–1.292
Metropolitan status (ref: Non-MSA)	MSA	1.20 ***	1.102–1.310	1.14 **	1.002–1.302	1.09	0.894–1.330
Indoor workers (ref: No)	Yes	0.99	0.891–1.090	1.17 **	1.006–1.366	1.09	0.873–1.348
Current smoking status (ref: Current Smokers)	Ex-Smokers (<1 year)	1.29 ***	1.099–1.507	1.50 ***	1.220–1.845	3.94 ***	3.056–5.078
	Ex-Smokers (<1 years) * Male	1.15	0.913–1.440	1.60 ***	1.198–2.128	1.49 **	1.055–2.097
Smoking frequency 12 month ago (ref: Everyday smokers)	Someday smokers	1.27 ***	1.099–1.466	1.45 ***	1.158–1.804	1.28	0.920–1.778
Cigarette per day 12 months ago (ref: 1–4)	5–14	1.50 ***	1.293–1.731	1.53 ***	1.218–1.932	1.54 **	1.093–2.174
	15–24	1.69 ***	1.442–1.983	1.95 ***	1.526–2.501	1.93 ***	1.337–2.796
	25 +	2.25 ***	1.858–2.730	2.67 ***	2.004–3.562	2.56 ***	1.668–3.912
First cigarette upon awakening ^§^ (ref: >30 min)	≤5 min	1.24 ***	1.114–1.386	1.07	0.914–1.247	1.09	0.866–1.374
	5–30 min	1.17 ***	1.069–1.271	0.97	0.854–1.104	0.86	0.704–1.043
Smokeless tobacco use ^†^ (ref: No)	Yes	4.56 ***	3.454–6.028	5.73 ***	2.137–15.375	1.87	0.096–36.491
	SLT use * Male	0.80	0.593–1.085	0.25 ***	0.088–0.715	0.28	0.013–5.931
State level cigarette tax ranking ^‡^ (ref: Lowest quarter)	Med-low quarter	1.02	0.898–1.168	0.95	0.787–1.155	1.25	0.934–1.682
	Med-high quarter	1.10	0.959–1.267	0.92	0.748–1.127	1.06	0.774–1.456
	Highest quarter	1.00	0.844–1.189	0.83	0.635–1.090	1.06	0.697–1.616
Tobacco control spending per capita ^‡^ (ref: Lowest quarter)	Med-low quarter	0.91	0.818–1.018	0.87 *	0.737–1.020	1.03	0.813–1.313
	Med-high quarter	0.90 *	0.798–1.008	0.90	0.760–1.063	1.01	0.788–1.305
	Highest quarter	0.82 ***	0.725–0.938	0.68 ***	0.567–0.827	0.73 **	0.548–0.975
State level worksite smoking ban (ref: Not highest level)	Highest level	1.03	0.923–1.152	1.02	0.864–1.208	0.92	0.713–1.179
State level worksite e-cigarette ban (ref: Not highest level)	Highest level	0.83 *	0.657–1.037	1.08	0.777–1.513	1.06	0.610–1.843
State level e-cigarette tax (ref: No)	Yes	1.16	0.864–1.549	0.82	0.522–1.300	1.07	0.572–2.008
Survey time (ref: July 2014)	Jan 2015	1.00	0.920–1.090	0.96	0.850–1.086	0.92	0.766–1.102
	May 2015	0.93	0.850–1.014	0.78 ***	0.683–0.891	0.80 **	0.659–0.973
Census division (ref: Pacific)	East North Central	0.83 **	0.700–0.988	0.91	0.699–1.171	0.82	0.555–1.216
	East South Central	0.81 **	0.677–0.960	0.90	0.696–1.164	0.98	0.677–1.424
	Middle Atlantic	0.86	0.714–1.040	0.96	0.714–1.276	0.94	0.606–1.455
	Mountain	1.02	0.849–1.215	1.29 *	0.995–1.680	1.25	0.844–1.863
	New England	0.78 **	0.635–0.961	0.94	0.692–1.280	0.87	0.540–1.392
	South Atlantic	0.86 *	0.730–1.003	0.92	0.723–1.166	1.05	0.735–1.492
	West North Central	0.84 *	0.700–1.015	0.98	0.749–1.285	0.98	0.652–1.476
	West South Central	0.74 ***	0.625–0.878	0.94	0.731–1.210	1.07	0.733–1.556

**^Δ^** Odds ratio and significance level of *p*-value: “***” < 0.01; 0.01 < “**” < 0.05; 0.05 < “*” ≤0.1. **^§^** The time to awakening reported for current smokers was their current habit and former smokers corresponded to time one year ago. ^†^ The smokeless tobacco use measure in each model corresponds to the e-cigarette use measure. **^‡^** Data for state level cigarette tax ranking are based on the January 2015 level and for per capita tobacco control spending are for the year 2015.

**Table 4:** Odds ratios for ever, current and regular use measures of e-cigarette use of former smokers who quit more than one year and less than or equal to three years, CPS-TUS, 2014/5.

Variable	Category	Ever Use of an E-cigarette		Current Use (≥1 of Past 30 Days)		Regular Use (≥20 of Past 30 Days)	
		OR ^Δ^	95% CI	OR	95% CI	OR	95% CI
**Intercept**		0.17 ***	0.073–0.376	0.06 ***	0.017–0.185	0.04 ***	0.009–0.163
Sex (ref: Female)	Male	0.44 ***	0.236–0.810	0.44	0.165–1.199	0.21 **	0.060–0.753
Age (ref: 35–44)	18–24	1.22	0.668–2.237	0.15 ***	0.035–0.626	0.03 ***	0.003–0.201
	25–34	1.11	0.827–1.492	0.63 **	0.395–0.992	0.63 *	0.371–1.078
	45–64	1.01	0.754–1.347	0.86	0.573–1.290	1.13	0.715–1.772
	65+	0.58 **	0.370–0.900	0.60	0.316–1.120	0.67	0.326–1.365
	Age 18–24 * Male	1.31	0.602–2.844	7.17 **	1.393–36.915	24.68 ***	2.398–254.059
Race (ref: White)	Asian	0.95	0.501–1.815	1.44	0.567–3.662	1.08	0.317–3.647
	Black	0.61 **	0.388–0.968	0.32 **	0.130–0.759	0.38 *	0.142–1.017
	Other races	1.22	0.740–2.024	1.38	0.665–2.879	1.82	0.850–3.895
Hispanic (ref: Non-Hispanic)	Hispanic	0.66 *	0.433–1.001	0.30 ***	0.130–0.714	0.25 ***	0.090–0.693
Education (ref: Less than 12 years)	High school degree	1.50 *	0.972–2.324	1.22	0.652–2.277	1.52	0.731–3.145
	Associate degree	1.67 **	1.084–2.577	1.58	0.834–2.974	1.50	0.719–3.139
	College degree or higher	1.49 *	0.938–2.362	0.73	0.364–1.460	0.82	0.369–1.837
Family income (ref: $0–$19,999)	$20,000–$39,999	0.98	0.627–1.534	0.95	0.474–1.905	0.87	0.395–1.910
	$40,000–$74,999	1.27	0.805–1.986	1.05	0.530–2.062	0.93	0.430–2.002
	$75,000 or more	0.78	0.485–1.256	1.02	0.494–2.087	0.90	0.394–2.039
	Income $20,000–$39,999 * Male	2.61 ***	1.281–5.325	2.78 *	0.866–8.950	6.54 ***	1.603–26.705
	Income $40,000–$74,999 * Male	1.43	0.707–2.879	1.67	0.539–5.201	4.08 **	1.013–16.406
	Income $75,000+ * Male	2.19 **	1.078–4.464	2.33	0.758–7.138	4.72 **	1.155–19.247
Marital status (ref: Married-Spouse Present)	Never Married	1.04	0.796–1.354	0.68 *	0.436–1.068	0.64	0.372–1.110
	Married-Spouse Absent or Widowed/Divorced/Separated	1.14	0.880–1.487	0.92	0.628–1.343	0.78	0.514–1.193
Employment status (ref: Employed)	Not in labor force or unemployed	0.98	0.708–1.364	1.17	0.718–1.913	1.21	0.667–2.193
Metropolitan status (ref: Non-MSA)	MSA	1.34 **	1.026–1.758	1.77 ***	1.168–2.671	1.64 **	1.043–2.591
Indoor workers (ref: No)	Yes	1.40 **	1.058–1.848	1.11	0.713–1.732	1.40	0.826–2.362
Smokeless tobacco use ^§^ (ref: No)	Yes	2.77 ***	2.096–3.654	0.52	0.189–1.410	0.41	0.109–1.542
State level cigarette tax ranking ^†^ (ref: Lowest quarter)	Med-low quarter	1.04	0.697–1.563	0.97	0.545–1.725	0.88	0.460–1.689
	Med-high quarter	1.31	0.879–1.962	1.47	0.822–2.616	1.42	0.729–2.763
	Highest quarter	1.89 **	1.137–3.141	1.60	0.702–3.643	1.69	0.653–4.392
Tobacco control spending per capita ^†^ (ref: Lowest quarter)	Med-low quarter	1.17	0.854–1.609	1.27	0.780–2.056	1.13	0.673–1.903
	Med-high quarter	1.09	0.774–1.526	0.95	0.580–1.569	0.70	0.405–1.193
	Highest quarter	1.09	0.746–1.588	1.28	0.707–2.299	1.02	0.552–1.897
State level worksite smoking ban (ref: Not highest level)	Highest level	0.73 *	0.529–1.013	0.66 *	0.408–1.076	0.77	0.442–1.348
State level worksite e–cigarette ban (ref: Not highest level)	Highest level	1.10	0.639–1.891	1.34	0.662–2.705	1.47	0.685–3.133
State level e-cigarette tax (ref: No)	Yes	0.93	0.417–2.071	2.03	0.635–6.468	1.12	0.301–4.156
Survey time (ref: July 2014)	Jan 2015	1.19	0.928–1.521	1.22	0.832–1.773	1.33	0.862–2.059
	May 2015	1.37 **	1.067–1.755	1.30	0.892–1.899	1.56 **	1.023–2.367
Census division (ref: Pacific)	East North Central	0.71	0.435–1.168	0.94	0.420–2.082	0.65	0.270–1.563
	East South Central	0.92	0.538–1.561	1.88	0.858–4.096	2.29 *	0.942–5.580
	Middle Atlantic	0.49 ***	0.288–0.823	0.99	0.441–2.201	0.81	0.322–2.021
	Mountain	1.72 *	0.988–2.993	1.72	0.758–3.918	1.33	0.499–3.536
	New England	0.36 ***	0.202–0.632	0.76	0.315–1.855	0.74	0.269–2.008
	South Atlantic	0.94	0.608–1.450	1.46	0.712–2.979	1.41	0.600–3.299
	West North Central	0.88	0.527–1.472	1.40	0.612–3.196	1.42	0.561–3.613
	West South Central	1.07	0.675–1.693	1.39	0.684–2.829	1.32	0.584–2.976
	Mountain * Male	0.72	0.379–1.371	1.57	0.657–3.739	2.88 **	1.104–7.496

**^Δ^** Odds ratio and significance level of *p*-value: “***” < 0.01; 0.01 < “**” < 0.05; 0.05 < “*” ≤ 0.1. **^§^** The smokeless tobacco use measure in each model corresponds to the e-cigarette use measure. ^†^ Data for state level cigarette tax ranking are based on the January 2015 level and for per capita tobacco control spending are for the year 2015.

**Table 5:** Odds ratios for ever, current and regular measures of e-cigarette use of e-cigarette use for former smokers who quit more than three years, CPS-TUS, 2014/5.

Variable	Category	Ever Use of an E-Cigarette		Current Use (≥1 of Past 30 Days)		Regular Use (≥20 of Past 30 Days)	
		OR^Δ^	95% CI	OR	95% CI	OR	95% CI
**Intercept**		0.02 ***	0.010–0.042	0.00 ***	0.001–0.013	0.00 ***	0.001–0.022
Sex (ref: Female)	Male	0.57	0.284–1.153	1.49	0.374–5.907	0.32	0.062–1.687
Age (ref: 35–44)	18–24	3.69 ***	2.095–6.490	1.11	0.260–4.730	0.83	0.119–5.741
	25–34	2.29 ***	1.796–2.914	1.48	0.877–2.483	1.54	0.773–3.084
	45–64	0.47 ***	0.377–0.594	0.49 ***	0.291–0.817	0.45 **	0.230–0.863
	65+	0.13 ***	0.090–0.178	0.14 ***	0.066–0.290	0.15 ***	0.062–0.382
Race (ref: White)	Asian	0.96	0.507–1.804	0.00 ***	0.000–0.000	0.00 ***	0.000–0.000
	Black	0.66 *	0.416–1.050	0.48	0.144–1.625	0.50	0.117–2.132
	Other races	1.09	0.630–1.885	1.63	0.629–4.215	0.28	0.040–1.934
Hispanic (ref: Non-Hispanic)	Hispanic	1.02	0.731–1.422	0.79	0.364–1.707	0.95	0.402–2.233
Education (ref: Less than 12 years)	High school degree	1.17	0.634–2.145	1.46	0.397–5.370	1.25	0.315–4.982
	Associate degree	1.48	0.818–2.676	1.06	0.300–3.742	0.79	0.207–2.978
	College degree or higher	1.03	0.554–1.915	0.63	0.165–2.411	0.36	0.077–1.639
Family income (ref: $0–$19,999)	$20,000–$39,999	0.81	0.591–1.098	1.44	0.731–2.832	1.34	0.577–3.131
	$40,000–$74,999	0.75 *	0.546–1.017	1.06	0.523–2.145	0.96	0.395–2.324
	$75,000 or more	0.94	0.673–1.303	0.94	0.411–2.142	0.71	0.271–1.858
Marital status (ref: Married-Spouse Present)	Never Married	1.73 ***	1.367–2.179	1.39	0.830–2.334	1.32	0.698–2.485
	Married-Spouse Absent or Widowed/Divorced/Separated	1.59 ***	1.277–1.988	1.71 **	1.037–2.817	1.17	0.651–2.105
Employment status (ref: Employed)	Not in labor force or unemployed	1.14	0.873–1.480	1.02	0.565–1.834	1.27	0.578–2.802
Metropolitan status (ref: Non-MSA)	MSA	1.43 ***	1.130–1.800	1.30	0.831–2.032	0.92	0.524–1.627
Indoor workers (ref: No)	Yes	1.05	0.842–1.318	1.14	0.680–1.913	1.39	0.699–2.746
Smokeless tobacco use ^§^ (ref: No)	Yes	3.52 ***	2.838–4.374	1.35	0.563–3.246	0.00 ***	0.000–0.000
State level cigarette tax ranking ^†^ (ref: Lowest quarter)	Med-low quarter	1.21	0.852–1.722	0.93	0.442–1.935	0.91	0.370–2.242
	Med-high quarter	1.27	0.892–1.800	1.03	0.497–2.145	1.02	0.428–2.428
	Highest quarter	1.50 *	0.984–2.295	1.60	0.621–4.126	1.31	0.444–3.890
Tobacco control spending per capita ^†^ (ref: Lowest quarter)	Med-low quarter	1.22	0.928–1.594	1.54	0.852–2.775	1.47	0.703–3.054
	Med-high quarter	1.16	0.883–1.516	1.44	0.793–2.615	1.16	0.525–2.562
	Highest quarter	1.04	0.750–1.432	1.20	0.569–2.515	1.03	0.364–2.931
State level worksite smoking ban	Highest level	0.83	0.626–1.092	1.01	0.570–1.790	1.07	0.535–2.145
State level worksite e-cigarette ban	Highest level	1.42	0.928–2.164	0.52	0.151–1.821	0.45	0.055–3.634
State level e-cigarette tax (ref: No)	Yes	0.51 *	0.240–1.068	1.14	0.291–4.495	3.11	0.509–18.966
Survey time (ref: July 2014)	Jan 2015	1.10	0.892–1.346	1.40	0.871–2.239	1.48	0.798–2.747
	May 2015	1.14	0.923–1.398	1.57 *	0.983–2.515	1.79 *	0.969–3.298
Census division (ref: Pacific)	East North Central	1.20	0.819–1.768	0.81	0.338–1.932	0.80	0.243–2.653
	East South Central	1.28	0.820–1.998	2.45 **	1.031–5.806	2.22	0.705–6.967
	Middle Atlantic	0.83	0.553–1.256	0.28 **	0.083–0.952	0.10 **	0.010–0.916
	Mountain	1.61 **	1.104–2.344	2.09 *	0.913–4.797	2.11	0.672–6.599
	New England	0.65 *	0.408–1.033	0.49	0.165–1.430	0.36	0.074–1.785
	South Atlantic	1.05	0.724–1.529	1.29	0.600–2.762	1.26	0.458–3.458
	West North Central	1.36	0.888–2.075	1.09	0.428–2.788	0.54	0.133–2.215
	West South Central	1.24	0.835–1.827	2.21 *	0.938–5.188	2.98 *	0.960–9.259

**^Δ^** Odds ratio and significance level of *p*-value: “***” < 0.01; 0.01 < “**” < 0.05; 0.05 < “*” ≤0.1. **^§^** The smokeless tobacco use measure in each model corresponds to the e-cigarette use measure. ^†^ Data for state level cigarette tax ranking are based on the January 2015 level and for per capita tobacco control spending are for the year 2015.

**Table 6:** Odds ratios for ever, current and regular measures of e-cigarette use for never smokers, CPS-TUS, 2014/5.

Variable	Category	Ever use of an E-Cigarette		Current Use (≥1 of Past 30 Days)		Regular Use (≥20 of Past 30 Days)	
		OR ^Δ^	95% CI	OR	95% CI	OR	95% CI
**Intercept**		0.01 ***	0.004–0.012	0.00 ***	0.000–0.004	0.00 ***	0.000–0.001
Sex (ref: Female)	Male	0.99	0.806–1.214	1.49 *	0.971–2.297	1.35	0.653–2.774
Age (ref: 35–44)	18–24	2.84 ***	2.246–3.594	2.44 ***	1.393–4.284	1.50	0.611–3.659
	25–34	1.67 ***	1.336–2.079	1.58 *	0.937–2.649	1.32	0.605–2.893
	45–64	0.44 ***	0.359–0.536	0.43 ***	0.275–0.679	0.22 ***	0.103–0.477
	65+	0.17 ***	0.116–0.239	0.23 ***	0.106–0.478	0.17 ***	0.056–0.540
	Age 18–34 * Male	1.55 ***	1.212–1.989	1.46	0.852–2.503	1.75	0.693–4.414
Race (ref: White)	Asian	0.53 ***	0.375–0.753	0.31 **	0.119–0.820	0.00 ***	0.000–0.000
	Black	0.43 ***	0.338–0.553	0.31 ***	0.180–0.524	0.25 ***	0.099–0.614
	Other races	1.47 ***	1.106–1.949	1.19	0.574–2.484	0.09 ***	0.023–0.364
Hispanic (ref: Non-Hispanic)	Hispanic	0.66 ***	0.543–0.813	0.53 ***	0.333–0.832	0.36 **	0.152–0.826
Education (ref: Less than 12 years)	High school degree	1.29	0.951–1.743	1.99 **	1.111–3.562	2.42 *	0.917–6.364
	Associate degree	1.69 ***	1.251–2.273	1.88 **	1.067–3.321	1.77	0.655–4.777
	College degree or higher	1.06	0.773–1.449	0.83	0.435–1.590	0.74	0.236–2.334
Family income (ref: $0–$19,999)	$20,000–$39,999	0.92	0.755–1.126	0.76	0.491–1.177	1.15	0.545–2.425
	$40,000–$74,999	0.81 **	0.662–0.989	0.65 **	0.421–0.986	0.99	0.479–2.029
	$75,000 or more	0.88	0.714–1.088	0.95	0.609–1.484	1.52	0.722–3.198
Marital status (ref: Married-Spouse Present)	Never Married	2.06 ***	1.754–2.411	1.80 ***	1.231–2.630	2.04 **	1.091–3.801
	Married-Spouse Absent or Widowed/Divorced/Separated	1.48 ***	1.205–1.812	1.84 ***	1.212–2.782	2.72 ***	1.420–5.192
Employment status (ref: Employed)	Not in labor force or unemployed	0.92	0.745–1.125	0.74	0.473–1.167	0.66	0.314–1.402
Metropolitan status (ref: Non-MSA)	MSA	1.23 **	1.027–1.476	1.03	0.700–1.522	1.81	0.891–3.685
Indoor workers (ref: No)	Yes	1.09	0.922–1.298	0.95	0.661–1.364	0.71	0.393–1.284
Current smoking status (ref: Never smokers one year ago)	Current Smokers	30.66 ***	25.002–37.601	33.62 ***	24.620–45.899	37.77 ***	22.361–63.809
Smokeless tobacco use ^§^ (ref: No)	Yes	6.99 ***	5.833–8.375	1.53	0.745–3.157	0.60	0.065–5.515
State level cigarette tax ranking ^†^ (ref: Lowest quarter)	Med-low quarter	1.11	0.881–1.407	1.23	0.753–2.016	1.38	0.655–2.924
	Med-high quarter	1.13	0.888–1.427	1.34	0.808–2.209	1.17	0.521–2.626
	Highest quarter	1.68 ***	1.257–2.233	1.17	0.625–2.171	1.20	0.495–2.930
Tobacco control spending per capita ^†^ (ref: Lowest quarter)	Med-low quarter	1.23 **	1.010–1.504	1.57 **	1.021–2.421	2.52 ***	1.275–4.990
	Med-high quarter	1.00	0.822–1.223	1.00	0.651–1.530	1.64	0.766–3.524
	Highest quarter	0.85	0.680–1.067	0.89	0.550–1.444	1.31	0.514–3.350
State level worksite smoking ban (ref: Not highest level)	Highest level	0.80 **	0.664–0.972	0.88	0.591–1.295	0.84	0.460–1.542
State level worksite e-cigarette ban (ref: Not highest level)	Highest level	0.64 **	0.444–0.915	1.24	0.578–2.658	1.75	0.488–6.299
State level e-cigarette tax (ref: No)	Yes	0.53 **	0.296–0.958	0.82	0.233–2.895	1.72	0.280–10.549
Survey time (ref: July 2014)	Jan 2015	1.09	0.938–1.267	0.91	0.643–1.281	0.60 *	0.332–1.095
	May 2015	1.03	0.881–1.199	0.87	0.623–1.207	1.07	0.625–1.845
Census division (ref: Pacific)	East North Central	0.73 **	0.549–0.981	0.80	0.407–1.556	1.01	0.340–3.011
	East South Central	0.59 ***	0.426–0.804	0.79	0.390–1.601	1.43	0.509–4.036
	Middle Atlantic	0.70 **	0.512–0.958	0.57	0.273–1.200	0.34	0.093–1.258
	Mountain	1.28 *	0.970–1.679	1.09	0.574–2.070	1.64	0.543–4.938
	New England	0.48 ***	0.330–0.697	0.58	0.251–1.340	0.99	0.223–4.361
	South Atlantic	0.69 ***	0.537–0.891	1.09	0.639–1.841	1.40	0.553–3.534
	West North Central	0.83	0.616–1.123	0.82	0.422–1.605	1.05	0.310–3.531
	West South Central	0.88	0.662–1.161	1.11	0.608–2.030	1.00	0.329–3.011

**^Δ^** Odds ratio and significance level of *p*-value: “***” < 0.01; 0.01 < “**” < 0.05; 0.05 < “*” ≤0.1. **^§^** The smokeless tobacco use measure in each model corresponds to the e-cigarette use measure; ^†^ Data for state level cigarette tax ranking are based on the January 2015 level and for per capita tobacco control spending are for the year 2015.

## Supplementary Materials

The following are available online at www.mdpi.com/1660-4601/14/10/1200/s1, Supplementary Table S1a: Ever, Current (at Least Once in the Last 30 Days), 5 or More, 10 or More, 20 or More of the Last 30 Days Measures of E-cigarette Use, Smokers 12 Months Ago, TUS-CPS, May 2014, Table S1b: Ever, Current (at Least Once in the Last 30 Days), 5 or More, 10 or More, 20 or More of the Last 30 Days Measures of E-cigarette Use, Former Smokers Who Quit More Than One Year and Less Than or Equal to Three Years, TUS-CPS, May 2014, Table S1c: Ever, Current (at Least Once in the Last 30 Days), 5 or More, 10 or More, 20 or More of the Last 30 Days Measures of E-cigarette Use, Former Smokers Who Quit More Than Three Years, TUS-CPS, May 2014, Table S1d: Ever, Current (at Least Once in the Last 30 Days), 5 or More, 10 or More, 20 or More of the Last 30 Days Measures of E-cigarette Use, Never Smokers 12 Months Ago, TUS-CPS, May 2014, Table S1e: Ever, Current (at Least Once in the Last 30 Days), 5 or More, 10 or More, 20 or More of the Last 30 Days Measures of E-cigarette Use, Total Males, TUS-CPS, May 2014, Table S1f: Ever, Current (at Least Once in the Last 30 Days), 5 or More, 10 or More, 20 or More of the Last 30 Days Measures of E-cigarette Use, Total Females, TUS-CPS, May 2014, Table S2a: Logistic Regression Equations, Male Smokers 12 Months Ago, Ever, Current (at Least Once in the Last 30 Days), 5 or More, 10 or More, 20 or More of the Last 30 Days Measures of E-cigarette Use, TUS-CPS, May 2014, Table S2b: Logistic Regression Equations, Female Smokers 12 Months Ago, Ever, Current (at Least Once in the Last 30 Days), 5 or More, 10 or More, 20 or More of the Last 30 Days Measures of E-cigarette Use, TUS-CPS, May 2014, Table S3a: Logistic Regression Equations, Male Former Smokers Who Quit More Than One Year and Less Than or Equal to Three Years, Ever, Current (at Least Once in the Last 30 Days), 5 or More, 10 or More, 20 or More of the Last 30 Days Measures of E-cigarette Use, TUS-CPS, May 2014, Table S3b: Logistic Regression Equations, Female Former Smokers Who Quit More Than One Year and Less Than or Equal to Three Years, Ever, Current (at Least Once in the Last 30 Days), 5 or More, 10 or More, 20 or More of the Last 30 Days Measures of E-cigarette Use, TUS-CPS, May 2014, Table S4a: Logistic Regression Equations, Male Former Smokers Who Quit More Than Three Years, Ever, Current (at Least Once in the Last 30 Days), 5 or More, 10 or More, 20 or More of the Last 30 Days Measures of E-cigarette Use, TUS-CPS, May 2014, Table S4b: Logistic Regression Equations, Female Former Smokers Who Quit More Than Three Years, Ever, Current (at Least Once in the Last 30 Days), 5 or More, 10 or More, 20 or More of the Last 30 Days Measures of E-cigarette Use, TUS-CPS, May 2014, Table S5a: Logistic Regression Equations, Male Never Smokers 12 Months Ago, Ever, Current (at Least Once in the Last 30 Days), 5 or More, 10 or More, 20 or More of the Last 30 Days Measures of E-cigarette Use, TUS-CPS, May 2014, Table S5b: Logistic Regression Equations, Female Never Smokers 12 Months Ago, Ever, Current (at Least Once in the Last 30 Days), 5 or More, 10 or More, 20 or More of the Last 30 Days Measures of E-cigarette Use, TUS-CPS, 2014/5.
