# Supplementary material for: The Prevalence and Characteristics of E-Cigarette Users in the U.S
Source: Int J Environ Res Public Health. 2017 Oct 11;14(10):1200. doi: 10.3390/ijerph14101200 (PMC5664701; doi:10.3390/ijerph14101200)
Supplement: Supplementary file 1 [file ijerph-14-01200-s001.pdf]

International Journal of
Environmental Research
and Public Health

Supplementary Materials

List of Tables: (supplementary)

Supplementary Table S1a. Ever, Current (at Least Once in the Last 30 Days), 5 or More, 10 or More, 20 or More of the Last 30 Days Measures of E–cigarette Use, Smokers 12 Months Ago, TUS-CPS, 2014/5
Supplementary Table S1b. Ever, Current (at Least Once in the Last 30 Days), 5 or More, 10 or More, 20 or More of the Last 30 Days Measures of E-cigarette Use, Former Smokers Who Quit More Than One Year and Less Than or Equal to Three Years, TUS-CPS, 2014/5
Supplementary Table S1c. Ever, Current (at Least Once in the Last 30 Days), 5 or More, 10 or More, 20 or More of the Last 30 Days Measures of E-cigarette Use, Former Smokers Who Quit More Than Three Years, TUS-CPS, 2014/5
Supplementary Table S1d. Ever, Current (at Least Once in the Last 30 Days), 5 or More, 10 or More, 20 or More of the Last 30 Days Measures of E-cigarette Use, Never Smokers 12 Months Ago, TUS-CPS, 2014/5
Supplementary Table S1e. Ever, Current (at Least Once in the Last 30 Days), 5 or More, 10 or More, 20 or More of the Last 30 Days Measures of E-cigarette Use, Total Males, TUS-CPS, 2014/5
Supplementary Table 1f. Ever, Current (at Least Once in the Last 30 Days), 5 or More, 10 or More, 20 or More of the Last 30 Days Measures of E-cigarette Use, Total Females, TUS-CPS, 2014/5
Supplementary Table S2a. Logistic Regression Equations, Male Smokers 12 Months Ago, Ever, Current (at Least Once in the Last 30 Days), 5 or More, 10 or More, 20 or More of the Last 30 Days Measures of E-cigarette Use, TUS-CPS, 2014/5
Supplementary Table S2b. Logistic Regression Equations, Female Smokers 12 Months Ago, Ever, Current (at Least Once in the Last 30 Days), 5 or More, 10 or More, 20 or More of the Last 30 Days Measures of E-cigarette Use, TUS-CPS, 2014/5
Supplementary Table S3a. Logistic Regression Equations, Male Former Smokers Who Quit More Than One Year and Less Than or Equal to Three Years, Ever, Current (at Least Once in the Last 30 Days), 5 or More, 10 or More, 20 or More of the Last 30 Days Measures of E-cigarette Use, TUS-CPS, 2014/5
Supplementary Table S3b. Logistic Regression Equations, Female Former Smokers Who Quit More Than One Year and Less Than or Equal to Three Years, Ever, Current (at Least Once in the Last 30 Days), 5 or More, 10 or More, 20 or More of the Last 30 Days Measures of E-cigarette Use, TUS-CPS, 2014/5
Supplementary Table S4a. Logistic Regression Equations, Male Former Smokers Who Quit More Than Three Years, Ever, Current (at Least Once in the Last 30 Days), 5 or More, 10 or More, 20 or More of the Last 30 Days Measures of E-cigarette Use, TUS-CPS, 2014/5
Supplementary Table S4b. Logistic Regression Equations, Female Former Smokers Who Quit More Than Three Years, Ever, Current (at Least Once in the Last 30 Days), 5 or More, 10 or More, 20 or More of the Last 30 Days Measures of E-cigarette Use, TUS-CPS, 2014/5
Supplementary Table S5a. Logistic Regression Equations, Male Never Smokers 12 Months Ago, Ever, Current (at Least Once in the Last 30 Days), 5 or More, 10 or More, 20 or More of the Last 30 Days Measures of E-cigarette Use, TUS-CPS, 2014/5
Supplementary Table S5b. Logistic Regression Equations, Female Never Smokers 12 Months Ago, Ever, Current (at Least Once in the Last 30 Days), 5 or More, 10 or More, 20 or More of the Last 30 Days Measures of E-cigarette Use, TUS-CPS, 2014/5

Supplementary Table S1a. Ever, Current (at Least Once in the Last 30 Days), 5 or More, 10 or More, 20 or More of the Last 30 Days Measures of E-cigarette Use, Smokers 12 Months Ago, TUS-CPS, 2014/5

Variable	Categories	Sample Size	Ever use	χ2 p	Current use	χ2 p	5 more days	χ2 p	10 more days	χ2 p	20 more days	χ2 p	Ratio (current /ever use)	Ratio (regular /current use)
**Overall**		**21300**	**38.7%**		**11.4%**		**8.3%**		**6.9%**		**4.8%**		**29.6%**	**42.0%**
Gender	Male	10554	37.0%	24.6	10.7%	10.0	8.2%	0.5	7.0%	0.4	5.0%	2.3	29.0%	46.8%
	Female	10746	40.3%	<0.001	12.1%	0.002	8.5%	0.488	6.8%	0.530	4.6%	0.130	30.1%	37.8%
Age	18–24	1469	54.2%		14.6%		11.6%		9.7%		6.5%		26.9%	44.4%
	25–34	4181	47.2%		12.7%		9.2%		7.4%		5.3%		26.9%	42.0%
	35–44	4026	42.8%		12.9%		9.5%		7.7%		5.3%		30.1%	40.8%
	45–64	9013	34.5%	599.5	10.6%	63.3	7.5%	54.8	6.3%	40.9	4.4%	25.0	30.8%	41.6%
	65+	2611	24.3%	<0.001	8.1%	<0.001	6.2%	<0.001	5.2%	<0.001	3.6%	<0.001	33.4%	43.9%
Race	White	17852	40.4%		12.1%		8.9%		7.3%		5.1%		29.9%	42.4%
	Black	2182	24.2%		6.0%		3.7%		2.9%		2.1%		25.0%	34.8%
	Asian	399	31.3%	230.8	8.3%	75.7	7.0%	70.1	6.8%	61.1	3.5%	40.9	26.4%	42.4%
	Other Races	867	42.9%	<0.001	12.8%	<0.001	8.9%	<0.001	8.0%	<0.001	5.4%	<0.001	29.8%	42.3%
Hispanic	Hispanic	1338	29.5%	50.4	8.4%	12.6	5.5%	15.6	4.6%	11.2	3.2%	7.8	28.6%	38.1%
	Non-Hispanic	19962	39.3%	<0.001	11.6%	<0.001	8.5%	<0.001	7.0%	0.001	4.9%	0.005	29.6%	42.2%
Education	Less than 12 years	3325	31.0%		8.6%		6.4%		4.8%		3.4%		27.8%	39.5%
	High school degree	8374	36.7%		10.7%		7.9%		6.6%		4.6%		29.2%	43.2%
	Associate degree	6947	43.9%	181.5	13.5%	59.2	9.7%	36.6	8.0%	36.2	5.6%	24.4	30.7%	41.5%
	College degree or higher	2654	41.0%	<0.001	11.9%	<0.001	8.7%	<0.001	7.4%	<0.001	5.0%	<0.001	29.1%	42.1%
Family income	$0 – $19,999	5932	35.6%		10.6%		7.8%		6.4%		4.5%		29.9%	42.1%
	$20,000 – $39,999	6014	37.9%		11.6%		8.5%		6.8%		4.7%		30.7%	40.4%
	$40,000 – $74,999	5558	39.7%	63.1	11.2%	10.1	7.9%	12.2	6.5%	15.6	4.6%	9.4	28.3%	41.0%
	$75,000 or more	3796	43.3%	<0.001	12.7%	0.018	9.6%	0.007	8.3%	0.001	5.7%	0.024	29.2%	45.3%
Marital status	Never Married	6062	41.4%		11.2%		8.3%		6.7%		4.7%		27.0%	41.8%
	Married-Spouse Present	7938	39.0%	40.1	12.0%	3.6	8.5%	0.4	7.0%	0.5	4.9%	0.3	30.7%	40.5%
	Married-Spouse Absent or Widowed/Divorced/Separated	7300	36.1%	<0.001	11.1%	0.2	8.2%	0.8	6.9%	0.8	4.8%	0.9	30.7%	43.8%
Employment status	Employed	12341	41.1%	71.0	11.6%	0.9	8.7%	5.8	7.3%	8.4	5.2%	8.9	28.3%	44.6%
	Not in labor force or unemployed	8959	35.4%	<0.001	11.2%	0.355	7.8%	0.016	6.3%	0.004	4.3%	0.003	31.7%	38.3%
Metropolitan status	MSA	15070	38.9%	1.6	11.7%	2.9	8.6%	3.6	7.1%	5.2	5.0%	3.6	30.0%	42.6%
	Non-MSA	6230	38.0%	0.202	10.9%	0.087	7.8%	0.056	6.3%	0.023	4.4%	0.058	28.5%	40.2%
Indoor workers	Yes	7908	41.8%	51.7	12.2%	6.7	9.2%	10.7	7.6%	10.7	5.2%	5.6	29.1%	43.1%
	No	13392	36.8%	<0.001	11.0%	0.010	7.9%	0.001	6.4%	0.001	4.5%	0.018	29.9%	41.2%
Current smoking status	Current smokers	19002	37.7%	72.2	10.6%	126.9	7.3%	265.8	5.7%	389.4	3.6%	563.6	28.1%	33.9%
	Ex-smokers (<1 year)	2298	46.8%	<0.001	18.5%	<0.001	17.2%	<0.001	16.7%	<0.001	14.8%	<0.001	39.5%	80.0%
Smoking frequency	Everyday smokers	17578	39.3%	14.7	11.6%	1.9	8.4%	0.3	7.0%	1.1	4.9%	3.0	29.5%	42.5%
	Someday smokers	3722	35.9%	<0.001	10.8%	0.165	8.1%	0.616	6.5%	0.291	4.2%	0.082	30.0%	39.4%
Cigarette per day	1–4	3849	32.7%		9.0%		6.7%		5.2%		3.7%		27.7%	40.5%
	5–14	7629	37.5%		10.6%		7.8%		6.4%		4.4%		28.3%	41.8%

	15–24	7896	41.3%	115.6	12.5%	62.0	9.0%	43.5	7.5%	40.4	5.2%	33.1	30.2%	41.8%
	25+	1926	44.7%	<0.001	15.2%	<0.001	11.3%	<0.001	9.2%	<0.001	6.8%	<0.001	33.9%	44.9%
First cigarette upon awakening§	≤ 5 min	3786	43.7%		13.1%		9.3%		7.9%		5.9%		30.0%	45.3%
	5–30 min	7196	39.5%	69.0	11.4%	14.6	8.1%	5.8	6.7%	7.9	4.3%	14.3	28.9%	38.1%
	>30 min	10318	36.2%	<0.001	10.8%	0.0	8.2%	0.1	6.6%	0.0	4.7%	0.0	29.9%	43.4%
Smokeless tobacco use†	Yes	2956	65.1%	1007.34	16.7%	14.10	9.9%	1.34	7.6%	0.31	4.5%	0.06	-	-
	No	18344	34.4%	<0.001	11.3%	0.0	8.3%	0.2	6.9%	0.6	4.8%	0.8	-	-
State level cigarette tax ranking‡	Lowest quarter	5263	39.1%		12.5%		9.1%		7.4%		5.0%		31.9%	40.1%
	Med-low quarter	6307	39.1%		11.6%		8.8%		7.4%		5.2%		29.7%	45.0%
	Med-high quarter	5205	39.1%	5.6	11.2%	12.5	7.8%	12.2	6.3%	10.9	4.4%	6.3	28.7%	39.5%
	Highest quarter	4525	37.1%	0.132	10.2%	0.006	7.4%	0.007	6.2%	0.012	4.4%	0.099	27.5%	43.0%
Tobacco control spending per capita‡	Lowest quarter	5647	39.0%		12.7%		9.2%		7.6%		5.0%		32.5%	39.5%
	Med-low quarter	5817	38.4%		10.9%		8.2%		6.7%		4.8%		28.5%	43.6%
	Med-high quarter	5628	38.5%	0.6	11.7%	18.1	8.4%	11.0	7.1%	11.2	5.2%	7.3	30.3%	44.3%
	Highest quarter	4208	38.8%	0.894	10.1%	<0.001	7.4%	0.012	5.9%	0.011	4.1%	0.064	26.0%	40.2%
State level worksite smoking ban	Highest level	13252	38.6%	0.2	10.9%	8.3	7.9%	10.4	6.5%	9.7	4.5%	7.3	28.4%	41.0%
	Not highest level	8048	38.8%	0.690	12.2%	0.004	9.1%	0.001	7.6%	0.002	5.3%	0.007	31.5%	43.4%
State level worksite e-cigarette ban	Highest level	785	38.5%	0.0	11.5%	0.0	7.9%	0.2	6.6%	0.1	4.3%	0.4	29.8%	37.8%
	Not highest level	20515	38.7%	0.907	11.4%	0.976	8.4%	0.643	6.9%	0.779	4.8%	0.533	29.6%	42.1%
State level e-cigarette tax	yes	396	45.2%	7.3	10.4%	0.5	8.3%	0.0	7.6%	0.3	6.1%	1.4	22.9%	58.5%
	no	20904	38.5%	0.007	11.5%	0.496	8.3%	0.992	6.9%	0.577	4.8%	0.235	29.7%	41.7%
Census division	East North Central	2791	39.8%		11.5%		8.5%		6.7%		4.7%		28.9%	40.5%
	East South Central	1966	36.9%		11.5%		9.0%		7.5%		5.0%		31.1%	43.8%
	Middle Atlantic	1573	37.4%		10.4%		7.5%		6.1%		4.2%		27.7%	40.5%
	Mountain	2387	41.9%		14.1%		9.8%		8.3%		5.7%		33.7%	40.1%
	New England	1765	36.1%		9.9%		6.7%		5.7%		4.1%		27.4%	41.1%
	Pacific	1953	42.2%		12.3%		8.9%		7.7%		5.6%		29.1%	45.4%
	South Atlantic	3884	35.2%		10.4%		7.5%		6.2%		4.5%		29.5%	43.4%
	West North Central	2517	42.3%	65.8	12.0%	30.3	8.9%	21.5	7.2%	18.9	4.6%	10.9	28.5%	38.6%
	West South Central	2464	37.3%	<0.001	10.8%	<0.001	8.4%	0.006	6.7%	0.015	4.8%	0.206	29.0%	44.6%

§ The time reported for current smokers was their current habit while for quitters in last year was their habit one year ago; † Sample size for smokeless tobacco use was in terms of ever use. For ever use, current use, 5 more days, 10 more days and 20 more days in last month, the number of respondents who use SLT with the same measure of e-cigarette are 2956, 496, 392, 327, and 246. The prevalence of e-cigarette use by five measures was estimated among SLT users who were categorized by the same measure; ‡ Data for state level cigarette tax ranking are in Jan 2015 and tobacco control spending per capita are in 2015.

Supplementary Table S1b. Ever, Current (at Least Once in the Last 30 Days), 5 or More, 10 or More, 20 or More of the Last 30 Days Measures of E-cigarette Use, Former Smokers Who Quit More Than One Year and Less Than or Equal to Three Years, TUS-CPS, 2014/5

Variable	Categories	Sample Size	Ever use	χ^2 p	Current use	χ^2 p	5 more days	χ^2 p	10 more days	χ^2 p	20 more days	χ^2 p	Ratio (current to ever use)	Ratio (20 days to current use)
Overall		**2892**	**30.2%**		**9.8%**		**9.0%**		**8.5%**		**7.4%**		**32.5%**	**75.4%**
Gender	Male	1467	30.8%	0.5	9.3%	0.8	8.8%	0.2	8.5%	0.0	7.3%	0.0	30.3%	78.1%
	Female	1425	29.6%	0.483	10.3%	0.377	9.3%	0.659	8.6%	0.863	7.5%	0.825	34.8%	72.8%
Age	18–24	168	38.1%		7.1%		6.0%		6.0%		3.6%		18.8%	50.0%
	25–34	776	34.4%		7.3%		6.3%		5.9%		5.2%		21.3%	70.2%
	35–44	546	32.6%		11.9%		11.2%		10.3%		8.8%		36.5%	73.8%
	45–64	1012	29.4%	44.6	11.8%	15.2	11.1%	18.3	10.6%	16.6	9.5%	18.2	39.9%	80.7%
	65+	390	17.2%	<0.001	7.9%	0.4%	7.4%	0.1%	7.2%	0.2%	6.2%	0.1%	46.3%	77.4%
Race	White	2526	30.8%		10.1%		9.3%		8.8%		7.6%		32.9%	75.0%
	Black	206	18.4%		3.4%		2.9%		2.9%		2.4%		18.4%	71.4%
	Asian	57	33.3%	17.1	12.3%	11.9	8.8%	12.3	8.8%	11.9	7.0%	11.7	36.8%	57.1%
	Other Races	103	37.9%	0.001	13.6%	0.008	13.6%	0.006	13.6%	0.008	12.6%	0.009	35.9%	92.9%
Hispanic	Hispanic	210	23.8%	4.4	4.8%	6.5	3.8%	7.5	3.3%	7.9	3.3%	5.5	20.0%	70.0%
	Non-Hispanic	2682	30.7%	0.036	10.2%	0.011	9.4%	0.006	8.9%	0.005	7.7%	0.019	33.3%	75.5%
Education	Less than 12 years	282	18.8%		7.1%		6.0%		6.0%		5.0%		37.7%	70.0%
	High school degree	882	27.9%		10.0%		9.4%		9.3%		8.2%		35.8%	81.8%
	Associate degree	1034	34.4%	29.0	11.6%	8.5	10.6%	8.9	9.8%	7.7	8.3%	6.3	33.7%	71.7%
	College degree or higher	694	31.6%	<0.001	8.1%	0.036	7.3%	0.031	6.8%	0.053	6.1%	0.098	25.6%	75.0%
Family income	$0–$19,999	496	21.2%		6.9%		6.3%		5.8%		4.6%		32.4%	67.6%
	$20,000–$39,999	735	32.1%		9.9%		9.3%		9.1%		7.8%		30.9%	78.1%
	$40,000–$74,999	871	32.0%	23.3	9.9%	7.5	8.8%	7.6	8.4%	6.7	7.8%	6.9	30.8%	79.1%
	$75,000 or more	790	32.2%	<0.001	11.5%	0.057	10.8%	0.054	9.9%	0.081	8.4%	0.074	35.8%	72.5%
Marital status	Never Married	758	33.4%		6.9%		5.8%		5.7%		5.0%		20.6%	73.1%
	Married-Spouse Present	1332	30.0%	6.3	11.3%	10.8	10.5%	13.5	10.0%	11.6	8.7%	9.6	37.5%	77.3%
	Married-Spouse Absent or Widowed/Divorced/Separated	802	27.6%	0.0	10.2%	0.0	9.6%	0.0	8.9%	0.0	7.5%	0.0	37.1%	73.2%
Employment status	Employed	1835	33.8%	31.2	9.9%	0.1	9.2%	0.2	8.7%	0.2	7.6%	0.2	29.3%	76.4%
	Not in labor force or unemployed	1057	23.9%	<0.001	9.6%	0.815	8.7%	0.648	8.2%	0.651	7.1%	0.635	40.3%	73.5%
Metropolitan status	MSA	2205	30.7%	0.8	10.5%	4.5	9.7%	4.6	9.2%	4.6	7.9%	3.3	34.2%	75.3%
	Non-MSA	687	28.8%	0.360	7.7%	0.034	7.0%	0.033	6.6%	0.033	5.8%	0.071	26.8%	75.5%
Indoor workers	Yes	1263	35.6%	31.1	10.6%	1.6	9.7%	1.4	9.3%	1.5	8.2%	2.3	29.8%	77.6%
	No	1629	26.0%	<0.001	9.2%	0.209	8.5%	0.238	8.0%	0.221	6.8%	0.131	35.4%	73.3%
Smokeless tobacco use§	Yes	509	47.3%	85.9	6.5%	1.4	4.9%	2.3	5.1%	1.5	4.6%	1.0	-	-
	No	2383	26.6%	<0.001	9.9%	0.2	9.2%	0.1	8.7%	0.2	7.5%	0.3	-	-
State level cigarette tax ranking†	Lowest quarter	632	30.5%		11.4%		10.4%		9.8%		9.2%		37.3%	80.6%
	Med-low quarter	833	29.4%		8.6%		8.3%		7.4%		6.4%		29.4%	73.6%
	Med-high quarter	771	30.4%	0.4	9.7%	3.1	8.7%	2.2	8.3%	2.8	6.7%	4.8	32.1%	69.3%
	Highest quarter	656	30.8%	0.941	9.9%	0.380	9.0%	0.530	9.0%	0.421	7.8%	0.184	32.2%	78.5%

Tobacco control spending per capita†	Lowest quarter	752	30.3%		11.3%		11.0%		10.5%		9.7%		37.3%	85.9%
	Med-low quarter	754	28.4%		9.3%		8.5%		8.0%		6.6%		32.7%	71.4%
	Med-high quarter	788	31.9%	2.2	9.5%	2.6	8.2%	5.1	7.9%	5.1	6.5%	7.9	29.9%	68.0%
	Highest quarter	598	30.3%	0.530	9.0%	0.455	8.2%	0.168	7.7%	0.168	6.7%	0.048	29.8%	74.1%
State level worksite smoking ban	Highest level	1838	29.8%	0.5	9.2%	1.9	8.5%	1.4	8.2%	0.9	6.8%	2.6	31.1%	73.5%
	Not highest level	1054	31.0%	0.476	10.8%	0.173	9.9%	0.231	9.2%	0.335	8.4%	0.104	34.9%	78.1%
State level worksite e-cigarette ban	Highest level	135	28.9%	0.1	11.9%	0.7	11.1%	0.8	10.4%	0.6	9.6%	1.0	41.0%	81.3%
	Not highest level	2757	30.3%	0.730	9.7%	0.417	8.9%	0.386	8.5%	0.436	7.3%	0.311	32.1%	75.0%
State level e-cigarette tax	yes	56	37.5%	1.4	17.9%	4.2	14.3%	1.9	14.3%	2.4	10.7%	0.9	47.6%	60.0%
	no	2836	30.1%	0.231	9.7%	0.041	8.9%	0.165	8.4%	0.120	7.3%	0.339	32.1%	75.9%
Census division	East North Central	377	30.2%		8.2%		8.0%		7.2%		5.8%		27.2%	71.0%
	East South Central	186	25.8%		10.2%		10.2%		10.2%		9.7%		39.6%	94.7%
	Middle Atlantic	227	22.0%		8.8%		8.4%		8.4%		6.6%		40.0%	75.0%
	Mountain	373	39.4%		13.9%		12.1%		11.3%		10.5%		35.4%	75.0%
	New England	263	22.8%		8.4%		7.2%		6.8%		6.5%		36.7%	77.3%
	Pacific	327	32.1%		7.0%		6.1%		5.8%		5.5%		21.9%	78.3%
	South Atlantic	481	29.5%		9.1%		8.1%		7.5%		6.4%		31.0%	70.5%
	West North Central	356	28.7%	35.2	9.3%	16.4	8.4%	16.8	8.1%	16.3	6.5%	14.8	32.4%	69.7%
	West South Central	302	35.1%	<0.001	13.2%	0.037	13.2%	0.033	12.6%	0.038	10.3%	0.063	37.7%	77.5%

§ Sample size for smokeless tobacco use was in terms of ever use. For ever use, current use, 5 more days, 10 more days and 20 more days in last month, the number of respondents who use SLT with the same measure of e-cigarette are 509, 108, 103, 98, and 87. The prevalence of e-cigarette use by five measures was estimated among SLT users who were categorized by the same measure; † Data for state level cigarette tax ranking are in Jan 2015 and tobacco control spending per capita are in 2015.

Supplementary Table S1c. Ever, Current (at Least Once in the Last 30 Days), 5 or More, 10 or More, 20 or More of the Last 30 Days Measures of E-cigarette Use, Former Smokers Who Quit More Than Three Years, TUS-CPS, 2014/5

Variable	Categories	Sample Size	Ever use	χ2 p	Current use	χ2 p	5 more days	χ2 p	10 more days	χ2 p	20 more days	χ2 p	Ratio (current to ever use)	Ratio (20 days to current use)
**Overall**		**27578**	**3.1%**		**0.5%**		**0.4%**		**0.4%**		**0.3%**		**17.5%**	**59.1%**
Gender	Male	14674	3.3%	2.8	0.6%	4.4	0.5%	5.2	0.5%	6.5	0.4%	3.1	19.2%	59.8%
	Female	12904	2.9%	0.093	0.4%	0.036	0.3%	0.023	0.3%	0.011	0.3%	0.080	15.2%	57.9%
Age	18–24	91	25.3%		2.2%		2.2%		1.1%		1.1%		8.7%	50.0%
	25–34	1711	13.3%		1.8%		1.3%		1.3%		1.1%		13.6%	61.3%
	35–44	3033	6.5%		1.1%		0.9%		0.7%		0.5%		16.2%	50.0%
	45–64	10613	2.9%	1081.2	0.6%	103.7	0.5%	77.4	0.5%	68.0	0.3%	54.4	20.6%	57.8%
	65+	12130	0.8%	<0.001	0.2%	<0.001	0.1%	<0.001	0.1%	<0.001	0.1%	<0.001	21.1%	75.0%
Race	White	24793	3.1%		0.6%		0.5%		0.4%		0.3%		17.9%	61.2%
	Black	1603	1.9%		0.2%		0.2%		0.2%		0.1%		9.7%	66.7%
	Asian	533	3.2%	11.4	0.0%	10.3	0.0%	5.6	0.0%	5.6	0.0%	4.6	0.0%	-
	Other Races	649	4.5%	0.010	1.1%	0.016	0.3%	0.134	0.2%	0.131	0.2%	0.203	24.1%	14.3%
Hispanic	Hispanic	1414	4.7%	13.5	0.9%	4.0	0.8%	5.4	0.8%	5.1	0.6%	2.9	19.4%	61.5%
	Non-Hispanic	26164	3.0%	<0.001	0.5%	0.046	0.4%	0.020	0.4%	0.024	0.3%	0.091	17.3%	58.8%
Education	Less than 12 years	2460	2.0%		0.6%		0.4%		0.4%		0.3%		28.0%	50.0%
	High school degree	8065	2.3%		0.4%		0.4%		0.3%		0.3%		18.3%	67.6%
	Associate degree	8463	4.0%	49.0	0.7%	5.7	0.5%	2.9	0.5%	2.6	0.4%	4.5	17.2%	62.1%
	College degree or higher	8590	3.3%	<0.001	0.5%	0.126	0.4%	0.407	0.4%	0.459	0.3%	0.216	15.4%	51.2%
Family income	$0–$19,999	3994	3.0%		0.5%		0.4%		0.4%		0.3%		14.9%	61.1%
	$20,000–$39,999	6463	2.6%		0.7%		0.6%		0.5%		0.4%		25.4%	62.8%
	$40,000–$74,999	7915	2.8%	20.5	0.5%	2.8	0.4%	4.2	0.4%	3.4	0.3%	2.7	19.2%	57.1%
	$75,000 or more	9206	3.7%	<0.001	0.5%	0.428	0.4%	0.240	0.3%	0.336	0.3%	0.445	13.4%	56.5%
Marital status	Never Married	2669	7.0%		1.0%		0.8%		0.7%		0.6%		13.8%	57.7%
	Married-Spouse Present	16340	2.7%	154.0	0.5%	10.4	0.4%	7.8	0.4%	8.6	0.3%	5.7	18.1%	62.5%
	Married-Spouse Absent or Widowed/Divorced/Separated	8569	2.6%	<0.001	0.5%	0.0	0.4%	0.0	0.4%	0.0	0.3%	0.1	19.2%	53.5%
Employment status	Employed	13475	4.5%	164.3	0.7%	21.5	0.6%	15.7	0.5%	12.0	0.4%	6.6	16.8%	54.5%
	Not in labor force or unemployed	14103	1.8%	<0.001	0.3%	<0.001	0.3%	<0.001	0.3%	0.001	0.2%	0.010	19.0%	68.8%
Metropolitan status	MSA	20830	3.3%	9.3	0.6%	0.2	0.5%	0.4	0.4%	0.3	0.3%	0.1	16.9%	56.5%
	Non-MSA	6748	2.5%	0.002	0.5%	0.639	0.4%	0.515	0.4%	0.596	0.3%	0.716	19.9%	67.6%
Indoor workers	Yes	8548	4.7%	104.0	0.8%	16.4	0.7%	21.8	0.6%	15.6	0.5%	8.6	17.3%	58.0%
	No	19030	2.4%	<0.001	0.4%	<0.001	0.3%	<0.001	0.3%	<0.001	0.3%	0.003	17.7%	60.0%
Smokeless tobacco use§	Yes	3768	8.0%	356.5	1.6%	12.6	1.0%	4.1	0.8%	2.8	0.0%	1.8	-	-
	No	23810	2.3%	<0.001	0.5%	0.0	0.4%	0.0	0.4%	0.1	0.3%	0.2	-	-
State level cigarette tax ranking†	Lowest quarter	5302	2.9%		0.6%		0.5%		0.5%		0.4%		20.5%	65.6%
	Med-low quarter	7677	3.3%		0.6%		0.5%		0.5%		0.4%		19.5%	59.2%
	Med-high quarter	7147	3.2%	2.7	0.5%	3.4	0.4%	3.6	0.3%	2.5	0.3%	3.9	15.2%	60.0%
	Highest quarter	7452	2.9%	0.434	0.4%	0.331	0.4%	0.311	0.3%	0.468	0.2%	0.272	15.3%	51.5%

Tobacco control spending per capita†	Lowest quarter	6895	2.9%		0.5%		0.4%		0.4%		0.3%		17.2%	68.6%
	Med-low quarter	7310	2.9%		0.5%		0.5%		0.4%		0.3%		18.9%	60.0%
	Med-high quarter	8075	3.3%	2.9	0.6%	0.5	0.5%	0.8	0.4%	2.5	0.3%	1.2	17.7%	57.4%
	Highest quarter	5298	3.2%	0.404	0.5%	0.919	0.4%	0.846	0.3%	0.484	0.2%	0.761	15.7%	48.1%
State level worksite smoking ban	Highest level	17963	3.2%	0.6	0.5%	0.3	0.4%	0.2	0.4%	0.2	0.3%	2.0	16.6%	54.3%
	Not highest level	9615	3.0%	0.448	0.6%	0.599	0.5%	0.688	0.4%	0.698	0.4%	0.157	19.2%	67.3%
State level worksite e-cigarette ban	Highest level	1114	4.3%	5.7	0.3%	1.6	0.1%	3.3	0.1%	2.9	0.1%	1.9	6.3%	33.3%
	Not highest level	26464	3.0%	0.017	0.6%	0.208	0.5%	0.069	0.4%	0.090	0.3%	0.166	18.1%	59.6%
State level e-cigarette tax	yes	597	2.3%	1.1	0.8%	1.0	0.5%	0.0	0.5%	0.1	0.5%	0.6	35.7%	60.0%
	no	26981	3.1%	0.286	0.5%	0.317	0.4%	0.834	0.4%	0.708	0.3%	0.422	17.2%	59.0%
Census division	East North Central	3384	3.3%		0.5%		0.4%		0.4%		0.3%		15.2%	64.7%
	East South Central	1591	2.9%		0.8%		0.8%		0.7%		0.4%		26.1%	58.3%
	Middle Atlantic	2501	2.4%		0.2%		0.1%		0.1%		0.0%		6.6%	25.0%
	Mountain	3051	4.5%		0.9%		0.7%		0.6%		0.5%		20.3%	57.1%
	New England	3384	2.0%		0.2%		0.2%		0.2%		0.1%		11.6%	50.0%
	Pacific	3548	3.4%		0.5%		0.4%		0.4%		0.3%		15.0%	55.6%
	South Atlantic	4863	2.6%		0.5%		0.4%		0.4%		0.3%		20.6%	65.4%
	West North Central	3039	3.4%	45.1	0.6%	26.3	0.4%	23.1	0.4%	21.1	0.2%	25.2	16.7%	41.2%
	West South Central	2217	3.6%	<0.001	0.9%	0.001	0.8%	0.003	0.7%	0.007	0.7%	0.002	24.1%	78.9%

§ Sample size for smokeless tobacco use was in terms of ever use. For ever use, current use, 5 more days, 10 more days and 20 more days in last month, the number of respondents who use SLT with the same measure of e-cigarette are 3768, 643, 610, 598, and 545. The prevalence of e-cigarette use by five measures was estimated among SLT users who were categorized by the same measure; † Data for state level cigarette tax ranking are in Jan 2015 and tobacco control spending per capita are in 2015.

Supplementary Table S1d. Ever, Current (at Least Once in the Last 30 Days), 5 or More, 10 or More, 20 or More of the Last 30 Days Measures of E-cigarette Use, Never Smokers 12 Months Ago, TUS-CPS, 2014/5

Variable	Categories	Sample Size	Ever use	χ2 p	Current use	χ2 p	5 more days	χ2 p	10 more days	χ2 p	20 more days	χ2 p	Ratio (current to ever use)	Ratio (20 days to current use)
**Overall**		**106034**	**1.9%**		**0.3%**		**0.2%**		**0.2%**		**0.1%**		**17.5%**	**34.4%**
Gender	Male	43492	2.5%	153.7	0.4%	27.2	0.3%	17.9	0.2%	19.0	0.2%	13.5	17.5%	36.1%
	Female	62542	1.5%	<0.001	0.3%	<0.001	0.2%	<0.001	0.1%	<0.001	0.1%	<0.001	17.4%	32.3%
Age	18–24	8657	7.3%		1.3%		0.8%		0.6%		0.3%		17.8%	26.8%
	25–34	18840	3.9%		0.6%		0.4%		0.4%		0.2%		16.2%	37.8%
	35–44	18431	1.6%		0.3%		0.2%		0.2%		0.1%		17.4%	44.2%
	45–64	37396	0.7%	2371.9	0.1%	390.1	0.1%	230.7	0.1%	161.5	0.0%	99.8	18.6%	30.8%
	65+	22710	0.2%	<0.001	0.1%	<0.001	0.0%	<0.001	0.0%	<0.001	0.0%	<0.001	25.5%	42.9%
Race	White	85640	1.9%		0.3%		0.2%		0.2%		0.1%		17.8%	36.9%
	Black	12138	1.2%		0.2%		0.2%		0.1%		0.1%		18.1%	30.8%
	Asian	5383	1.2%	143.3	0.1%	22.6	0.0%	17.7	0.0%	14.0	0.0%	10.0	12.1%	0.0%
	Other Races	2873	4.4%	<0.001	0.7%	<0.001	0.4%	0.001	0.2%	0.003	0.1%	0.018	15.9%	15.0%
Hispanic	Hispanic	13700	1.9%	0.0	0.3%	0.2	0.2%	2.1	0.1%	2.4	0.1%	0.9	16.5%	28.6%
	Non-Hispanic	92334	1.9%	0.839	0.3%	0.621	0.2%	0.144	0.2%	0.118	0.1%	0.340	17.6%	35.2%
Education	Less than 12 years	9728	1.3%		0.3%		0.2%		0.2%		0.1%		21.1%	22.2%
	High school degree	26836	1.9%		0.4%		0.3%		0.2%		0.2%		23.3%	40.5%
	Associate degree	29222	2.8%	186.7	0.5%	84.0	0.4%	61.5	0.3%	47.3	0.2%	40.3	18.6%	34.0%
	College degree or higher	40248	1.4%	<0.001	0.1%	<0.001	0.1%	<0.001	0.1%	<0.001	0.0%	<0.001	9.9%	28.6%
Family income	$0–$19,999	16737	2.3%		0.5%		0.3%		0.2%		0.2%		20.7%	32.5%
	$20,000–$39,999	22943	2.0%		0.4%		0.2%		0.2%		0.1%		19.1%	36.4%
	$40,000–$74,999	28917	1.9%	39.8	0.3%	21.4	0.2%	8.7	0.2%	9.1	0.1%	7.7	15.6%	36.4%
	$75,000 or more	37437	1.6%	<0.001	0.2%	<0.001	0.2%	0.034	0.1%	0.027	0.1%	0.053	15.9%	32.3%
Marital status	Never Married	24738	4.5%		0.8%		0.5%		0.4%		0.2%		17.1%	31.3%
	Married-Spouse Present	57401	1.0%	1229.7	0.2%	203.6	0.1%	120.2	0.1%	76.7	0.1%	55.8	15.3%	33.0%
	Married-Spouse Absent or Widowed/Divorced/Separated	23895	1.2%	<0.001	0.3%	<0.001	0.2%	<0.001	0.2%	<0.001	0.1%	<0.001	23.7%	45.5%
Employment status	Employed	66199	2.2%	118.4	0.4%	10.4	0.3%	11.8	0.2%	10.3	0.1%	6.1	16.7%	35.6%
	Not in labor force or unemployed	39835	1.3%	<0.001	0.3%	0.001	0.2%	0.001	0.1%	0.001	0.1%	0.014	19.7%	31.4%
Metropolitan status	MSA	83965	1.9%	4.2	0.3%	0.8	0.2%	0.4	0.2%	0.1	0.1%	2.5	17.5%	36.0%
	Non-MSA	22069	1.7%	0.042	0.3%	0.381	0.2%	0.529	0.2%	0.729	0.1%	0.117	17.4%	27.3%
Indoor workers	Yes	46612	2.3%	75.7	0.4%	4.0	0.3%	1.4	0.2%	1.0	0.1%	0.6	16.1%	33.1%
	No	59422	1.6%	<0.001	0.3%	0.045	0.2%	0.231	0.2%	0.316	0.1%	0.434	19.1%	35.6%
Current smoking status	Current smokers	1380	35.0%	8298.2	9.6%	3635.8	7.5%	3172.9	6.2%	2843.1	3.7%	1587.4	27.3%	38.6%
	Never smokers	104654	1.4%	<0.001	0.2%	<0.001	0.1%	<0.001	0.1%	<0.001	0.1%	<0.001	14.3%	31.8%
Smokeless tobacco use§	Yes	4695	10.8%	2092.9	1.2%	29.1	0.8%	17.2	0.7%	22.5	0.1%	0.0	-	-
	No	101339	1.5%	<0.001	0.3%	<0.001	0.2%	<0.001	0.2%	<0.001	0.1%	0.9	-	-
	Lowest quarter	21631	1.8%		0.4%		0.3%		0.2%		0.1%		22.3%	34.1%

State level cigarette tax ranking†	Med-low quarter	31972	1.9%		0.3%		0.2%		0.2%		0.1%		16.9%	42.7%
	Med-high quarter	26764	2.0%	3.0	0.4%	7.3	0.2%	3.5	0.2%	4.2	0.1%	5.2	18.0%	26.3%
	Highest quarter	25667	1.9%	0.398	0.3%	0.062	0.2%	0.324	0.1%	0.240	0.1%	0.160	13.8%	33.3%
Tobacco control spending per capita†	Lowest quarter	27464	1.8%		0.3%		0.2%		0.1%		0.1%		16.6%	24.1%
	Med-low quarter	26310	1.8%		0.3%		0.2%		0.2%		0.1%		18.6%	39.1%
	Med-high quarter	34777	1.9%	5.7	0.3%	1.3	0.2%	1.3	0.2%	3.1	0.1%	5.4	17.2%	39.1%
	Highest quarter	17483	2.1%	0.128	0.4%	0.720	0.3%	0.737	0.2%	0.379	0.1%	0.142	17.7%	32.8%
State level worksite smoking ban	Highest level	65143	1.9%	0.5	0.3%	1.3	0.2%	0.2	0.2%	0.3	0.1%	0.0	16.8%	35.8%
	Not highest level	40891	1.9%	0.490	0.4%	0.251	0.2%	0.644	0.2%	0.559	0.1%	0.892	18.5%	32.4%
State level worksite e-cigarette ban	Highest level	5062	1.5%	4.6	0.3%	0.0	0.2%	0.3	0.1%	0.4	0.1%	0.1	21.3%	31.3%
	Not highest level	100972	1.9%	0.031	0.3%	0.868	0.2%	0.611	0.2%	0.508	0.1%	0.755	17.3%	34.5%
State level e-cigarette tax	yes	1917	1.8%	0.1	0.3%	0.3	0.2%	0.0	0.2%	0.0	0.2%	0.3	14.7%	60.0%
	no	104117	1.9%	0.721	0.3%	0.598	0.2%	0.837	0.2%	0.834	0.1%	0.569	17.5%	34.0%
Census division	East North Central	11428	1.9%		0.3%		0.2%		0.2%		0.1%		17.1%	40.5%
	East South Central	7112	1.6%		0.3%		0.3%		0.2%		0.1%		21.1%	37.5%
	Middle Atlantic	8865	1.4%		0.2%		0.1%		0.1%		0.0%		14.0%	23.5%
	Mountain	12876	2.4%		0.5%		0.4%		0.3%		0.2%		18.8%	46.6%
	New England	8771	1.3%		0.2%		0.1%		0.1%		0.0%		14.8%	23.5%
	Pacific	15102	2.4%		0.3%		0.2%		0.2%		0.1%		13.9%	30.0%
	South Atlantic	20399	1.6%		0.3%		0.2%		0.2%		0.1%		21.1%	34.3%
	West North Central	10486	2.1%	87.2	0.3%	18.5	0.2%	19.1	0.2%	15.6	0.1%	18.8	14.7%	30.3%
	West South Central	10995	2.0%	<0.001	0.4%	0.018	0.3%	0.015	0.2%	0.048	0.1%	0.016	20.9%	28.3%

§ Sample size for smokeless tobacco use was in terms of ever use. For ever use, current use, 5 more days, 10 more days and 20 more days in last month, the number of respondents who use SLT with the same measure of e-cigarette are 4695, 1388, 1273, 1207, and 1043. The prevalence of e-cigarette use by five measures was estimated among SLT users who were categorized by the same measure; † Data for state level cigarette tax ranking are in Jan 2015 and tobacco control spending per capita are in 2015.

Supplementary Table S1e. Ever, Current (at Least Once in the Last 30 Days), 5 or More, 10 or More, 20 or More of the Last 30 Days Measures of E-cigarette Use, Total Males, TUS-CPS, 2014/5

Variable	Categories	Sample Size	Ever use	χ2 p	Current use	χ2 p	5 more days	χ2 p	10 more days	χ2 p	20 more days	χ2 p	Ratio (current to ever use)	Ratio (20 days to current use)
**Overall**		**70588**	**8.6%**		**2.3%**		**1.8%**		**1.5%**		**1.1%**		**26.4%**	**49.7%**
Age	18–24	4808	18.2%		4.0%		3.1%		2.6%		1.9%		22.0%	48.4%
	25–34	11473	15.6%		3.7%		2.8%		2.4%		1.8%		24.0%	48.1%
	35–44	11867	10.2%		2.9%		2.4%		2.1%		1.5%		28.8%	52.0%
	45–64	26515	6.8%	2182.8	2.0%	384.4	1.6%	278.7	1.3%	232.6	1.0%	176.0	28.8%	48.7%
	65+	15925	2.4%	<0.001	0.7%	<0.001	0.6%	<0.001	0.5%	<0.001	0.4%	<0.001	29.1%	54.9%
Race	White	59238	8.9%		2.4%		1.9%		1.6%		1.2%		26.8%	51.0%
	Black	6385	5.7%		1.3%		0.9%		0.7%		0.5%		22.7%	39.8%
	Asian	3048	5.0%	193.1	1.1%	69.4	0.7%	63.3	0.7%	54.5	0.4%	43.7	21.7%	36.4%
	Other Races	1917	13.8%	<0.001	3.8%	<0.001	2.6%	<0.001	2.3%	<0.001	1.6%	<0.001	27.2%	41.7%
Hispanic	Hispanic	7375	5.7%	86.4	1.3%	32.2	0.9%	36.9	0.7%	35.3	0.5%	26.6	23.4%	39.4%
	Non-Hispanic	63213	8.9%	<0.001	2.4%	<0.001	1.9%	<0.001	1.6%	<0.001	1.2%	<0.001	26.6%	50.3%
Education	Less than 12 years	7096	8.9%		2.2%		1.7%		1.4%		1.0%		25.0%	44.3%
	High school degree	19993	10.3%		2.8%		2.2%		1.9%		1.5%		27.2%	52.0%
	Associate degree	19466	11.2%	657.4	3.2%	241.5	2.5%	180.7	2.1%	148.6	1.5%	119.9	28.2%	48.6%
	College degree or higher	24033	4.9%	<0.001	1.1%	<0.001	0.9%	<0.001	0.8%	<0.001	0.6%	<0.001	22.5%	50.4%
Family income	$0–$19,999	10232	11.1%		2.8%		2.1%		1.8%		1.3%		25.2%	47.9%
	$20,000–$39,999	15368	10.3%		3.0%		2.4%		2.0%		1.5%		29.2%	50.8%
	$40,000–$74,999	20068	8.7%	280.3	2.2%	88.3	1.7%	59.6	1.5%	50.3	1.1%	44.2	24.8%	49.3%
	$75,000 or more	24920	6.5%	<0.001	1.7%	<0.001	1.4%	<0.001	1.2%	<0.001	0.9%	<0.001	26.3%	50.0%
Marital status	Never Married	16970	14.2%		3.3%		2.6%		2.2%		1.6%		23.5%	47.4%
	Married-Spouse Present	39584	5.8%	1092.3	1.7%	165.2	1.3%	116.6	1.2%	88.1	0.9%	62.2	28.6%	51.7%
	Married-Spouse Absent or Widowed/Divorced/Separated	14034	9.6%	<0.001	2.7%	<0.001	2.1%	<0.001	1.8%	<0.001	1.3%	<0.001	27.9%	49.5%
Employment status	Employed	46738	9.3%	84.5	2.4%	14.1	1.9%	17.6	1.7%	14.5	1.2%	12.7	26.1%	50.8%
	Not in labor force or unemployed	23850	7.2%	<0.001	2.0%	<0.001	1.5%	<0.001	1.3%	<0.001	0.9%	<0.001	27.3%	47.0%
Metropolitan status	MSA	54742	8.3%	23.0	2.3%	0.5	1.8%	0.1	1.5%	0.3	1.1%	0.3	27.0%	49.6%
	Non-MSA	15846	9.5%	<0.001	2.3%	0.479	1.8%	0.742	1.6%	0.597	1.2%	0.603	24.6%	49.7%
Indoor workers	Yes	26872	9.3%	23.9	2.5%	11.4	2.0%	11.0	1.7%	9.7	1.3%	9.3	27.1%	51.0%
	No	43716	8.2%	<0.001	2.1%	0.001	1.7%	0.001	1.4%	0.002	1.0%	0.002	25.9%	48.7%
Current smoking status	Current smokers	10005	35.8%		9.7%		7.0%		5.7%		3.7%		27.0%	37.8%
	Never smokers	42853	2.0%		0.3%		0.2%		0.2%		0.1%		14.7%	33.6%
	Ex-smokers (<1 year)	1589	43.5%		17.7%		16.7%		16.2%		14.2%		40.7%	80.4%
	Ex-smokers (1–3 years)	1467	30.8%	15702.0	9.3%	5419.0	8.8%	4740.1	8.5%	4498.5	7.3%	3994.1	30.3%	78.1%
	Ex-smokers (≥3 years)	14674	3.3%	<0.001	0.6%	<0.001	0.5%	<0.001	0.5%	<0.001	0.4%	<0.001	19.2%	59.8%
Smoking frequency	Everyday smokers	8154	35.6%	0.89	9.3%	5.99	6.5%	18.58	5.2%	21.17	3.1%	40.30	25.9%	48.9%
	Someday smokers	1851	36.8%	0.3	11.2%	0.0	9.3%	<0.001	7.9%	<0.001	6.2%	<0.001	30.4%	55.1%

Cigarette per day	1–4	2011	33.2%		10.1%		8.6%		7.5%		5.9%		30.6%	57.8%
	5–14	3264	35.4%		10.4%		7.2%		5.9%		3.8%		29.4%	36.9%
	15–24	3746	37.1%	11.6	8.8%	5.5	6.0%	13.5	4.7%	20.1	2.6%	43.2	23.8%	29.0%
	25+	984	38.3%	0.0	9.5%	0.1	6.7%	0.0	4.8%	0.0	2.7%	<0.001	24.7%	29.0%
First cigarette upon awakening	≤5 min	1708	39.5%		9.5%		6.7%		5.7%		3.8%		24.0%	40.1%
	5–30 min	3405	36.4%	16.8	9.8%	0.1	6.5%	2.3	5.3%	1.0	3.2%	3.1	26.8%	32.7%
	>30 min	4892	34.1%	0.0	9.6%	0.9	7.4%	0.3	5.9%	0.6	3.9%	0.2	28.3%	40.7%
Smokeless tobacco use§	Yes	10637	23.3%	3434.81	4.3%	48.82	2.4%	5.98	1.9%	2.46	0.9%	1.21	-	-
	No	59951	6.0%	<0.001	2.2%	<0.001	1.8%	0.0	1.5%	0.1	1.1%	0.3	-	-
State level cigarette tax ranking†	Lowest quarter	14377	9.4%		2.8%		2.1%		1.9%		1.3%		29.9%	48.0%
	Med-low quarter	21076	8.8%		2.3%		1.9%		1.6%		1.1%		26.2%	49.3%
	Med-high quarter	17929	8.5%	24.3	2.2%	29.7	1.7%	20.2	1.4%	19.5	1.1%	9.5	25.7%	50.0%
	Highest quarter	17206	7.9%	<0.001	1.9%	<0.001	1.5%	<0.001	1.3%	<0.001	1.0%	0.024	24.1%	51.8%
Tobacco control spending per capita†	Lowest quarter	18235	8.5%		2.5%		1.9%		1.6%		1.2%		29.2%	47.6%
	Med-low quarter	17783	8.8%		2.3%		1.8%		1.6%		1.1%		25.8%	49.8%
	Med-high quarter	22108	8.1%	19.7	2.2%	5.1	1.7%	4.8	1.5%	2.6	1.1%	1.4	27.0%	52.4%
	Highest quarter	12462	9.5%	<0.001	2.2%	0.164	1.7%	0.184	1.4%	0.451	1.0%	0.696	22.7%	48.1%
State level worksite smoking ban	Highest level	44117	8.6%	0.2	2.2%	4.6	1.7%	3.2	1.5%	2.6	1.1%	1.4	25.2%	50.1%
	Not highest level	26471	8.6%	0.687	2.4%	0.032	1.9%	0.072	1.6%	0.104	1.2%	0.236	28.4%	49.0%
State level worksite e-cigarette ban	Highest level	3277	8.0%	1.6	2.2%	0.2	1.6%	1.0	1.3%	0.9	1.1%	0.1	27.1%	49.3%
	Not highest level	67311	8.6%	0.200	2.3%	0.674	1.8%	0.320	1.5%	0.351	1.1%	0.735	26.4%	49.7%
State level e-cigarette tax	yes	1379	10.2%	4.3	2.7%	1.1	2.3%	2.3	2.2%	4.7	1.7%	4.7	26.4%	64.9%
	no	69209	8.6%	0.039	2.3%	0.303	1.8%	0.126	1.5%	0.031	1.1%	0.030	26.4%	49.3%
Census division	East North Central	8062	9.7%		2.5%		2.0%		1.7%		1.2%		25.7%	49.3%
	East South Central	4758	9.4%		2.8%		2.4%		2.0%		1.5%		30.1%	53.3%
	Middle Atlantic	5796	7.0%		1.7%		1.3%		1.1%		0.8%		24.9%	48.5%
	Mountain	8554	9.5%		2.8%		2.2%		1.9%		1.4%		29.7%	50.8%
	New England	6428	6.9%		1.6%		1.2%		1.0%		0.8%		22.5%	49.0%
	Pacific	9481	8.1%		2.0%		1.4%		1.2%		0.9%		24.3%	46.8%
	South Atlantic	13021	7.6%		2.0%		1.6%		1.4%		1.0%		26.3%	48.7%
	West North Central	7488	10.6%	127.5	2.6%	58.9	2.0%	57.7	1.8%	48.2	1.3%	37.8	24.2%	49.5%
	West South Central	7000	9.0%	<0.001	2.7%	<0.001	2.2%	<0.001	1.8%	<0.001	1.4%	<0.001	29.8%	51.3%

§ Sample size for smokeless tobacco use was in terms of ever use. For ever use, current use, 5 more days, 10 more days and 20 more days in last month, the number of respondents who use SLT with the same measure of e-cigarette are 10637, 2531, 2290, 2156, and 1855. The prevalence of e-cigarette use by five measures was estimated among SLT users who were categorized by the same measure; † Data for state level cigarette tax ranking are in Jan 2015 and tobacco control spending per capita are in 2015.

Supplementary Table S1f. Ever, Current (at Least Once in the Last 30 Days), 5 or More, 10 or More, 20 or More of the Last 30 Days Measures of E-cigarette Use, Total Females, TUS-CPS, 2014/5

Variable	Categories	Sample Size	Ever use	χ2 p	Current use	χ2 p	5 more days	χ2 p	10 more days	χ2 p	20 more days	χ2 p	Ratio (current to ever use)	Ratio (20 days to current use)
**Overall**		**88038**	**7.0%**		**1.9%**		**1.4%**		**1.1%**		**0.8%**		**27.6%**	**41.3%**
Age	18–24	5662	11.9%		2.7%		1.9%		1.5%		0.8%		22.7%	28.1%
	25–34	14232	10.6%		2.4%		1.7%		1.3%		1.0%		22.5%	40.7%
	35–44	14324	8.6%		2.3%		1.7%		1.3%		0.9%		27.2%	36.9%
	45–64	31818	7.2%	1319.2	2.2%	215.1	1.6%	140.5	1.4%	105.1	1.0%	77.8	31.0%	45.2%
	65+	22002	2.2%	<0.001	0.8%	<0.001	0.6%	<0.001	0.5%	<0.001	0.4%	<0.001	35.2%	46.2%
Race	White	72226	7.5%		2.1%		1.5%		1.2%		0.9%		28.1%	41.6%
	Black	9863	4.1%		0.9%		0.6%		0.4%		0.3%		22.2%	33.7%
	Asian	3340	2.3%	363.9	0.5%	125.0	0.4%	96.4	0.4%	83.6	0.2%	61.4	21.1%	43.8%
	Other Races	2609	11.8%	<0.001	3.2%	<0.001	2.2%	<0.001	1.9%	<0.001	1.4%	<0.001	26.9%	43.4%
Hispanic	Hispanic	9349	4.0%	152.0	0.9%	57.5	0.6%	47.8	0.5%	37.2	0.4%	24.0	23.2%	40.7%
	Non-Hispanic	78689	7.4%	<0.001	2.1%	<0.001	1.5%	<0.001	1.2%	<0.001	0.9%	<0.001	27.9%	41.3%
Education	Less than 12 years	8807	7.5%		2.2%		1.7%		1.2%		0.9%		30.0%	38.4%
	High school degree	24424	8.3%		2.5%		1.8%		1.5%		1.0%		29.5%	42.3%
	Associate degree	26474	9.4%	789.6	2.6%	309.3	1.9%	235.7	1.5%	197.4	1.1%	145.1	28.1%	42.4%
	College degree or higher	28333	3.6%	<0.001	0.8%	<0.001	0.5%	<0.001	0.4%	<0.001	0.3%	<0.001	21.2%	37.3%
Family income	$0–$19,999	17124	9.6%		2.8%		2.1%		1.7%		1.2%		29.6%	40.4%
	$20,000–$39,999	21012	7.8%		2.3%		1.6%		1.3%		0.9%		29.0%	38.5%
	$40,000–$74,999	23405	6.9%	383.6	1.9%	166.6	1.3%	118.3	1.0%	89.2	0.8%	51.7	27.0%	42.5%
	$75,000 or more	26497	4.9%	<0.001	1.2%	<0.001	0.9%	<0.001	0.7%	<0.001	0.5%	<0.001	24.2%	45.0%
Marital status	Never Married	17522	10.0%		2.3%		1.6%		1.2%		0.8%		23.1%	34.5%
	Married-Spouse Present	43751	5.4%	447.6	1.5%	92.6	1.1%	82.9	0.9%	68.2	0.6%	49.9	27.8%	41.3%
	Married-Spouse Absent or Widowed/Divorced/Separated	26765	7.8%	<0.001	2.4%	<0.001	1.8%	<0.001	1.5%	<0.001	1.1%	<0.001	31.1%	45.5%
Employment status	Employed	47624	7.6%	53.9	1.9%	0.8	1.4%	0.0	1.2%	0.5	0.8%	0.5	25.0%	43.1%
	Not in labor force or unemployed	40414	6.4%	<0.001	2.0%	0.363	1.4%	0.870	1.1%	0.459	0.8%	0.491	31.3%	39.2%
Metropolitan status	MSA	67960	6.7%	65.2	1.8%	23.2	1.3%	15.1	1.1%	7.5	0.8%	3.9	27.3%	42.2%
	Non-MSA	20078	8.3%	<0.001	2.4%	<0.001	1.7%	<0.001	1.3%	0.006	0.9%	0.048	28.3%	38.7%
Indoor workers	Yes	50234	6.6%	33.4	2.0%	1.1	1.4%	0.0	1.1%	0.2	0.8%	0.0	30.1%	40.6%
	No	37804	7.6%	<0.001	1.9%	0.284	1.4%	0.825	1.2%	0.652	0.8%	0.869	24.8%	42.2%
Current smoking status	Current smokers	10345	39.4%		11.5%		7.7%		5.8%		3.5%		29.0%	30.9%
	Never smokers	61801	1.0%		0.1%		0.1%		0.1%		0.0%		13.8%	29.2%
	Ex-smokers (<1 year)	1563	43.3%		14.9%		13.4%		12.7%		11.1%		34.4%	74.7%
	Ex-smokers (1–3 years)	1425	29.6%	24573.8	10.3%	8016.2	9.3%	6067.8	8.6%	5306.8	7.5%	4373.1	34.8%	72.8%
	Ex-smokers (≥3 years)	12904	2.9%	<0.001	0.4%	<0.001	0.3%	<0.001	0.3%	<0.001	0.3%	<0.001	15.2%	57.9%
Smoking frequency	Everyday smokers	8473	40.0%	5.60	10.9%	14.55	7.1%	20.79	5.3%	20.07	3.1%	25.84	26.3%	41.6%
	Someday smokers	1872	37.0%	0.0	14.0%	0.0	10.2%	<0.001	8.0%	<0.001	5.5%	<0.001	37.8%	39.3%

Cigarette per day	1–4	2173	35.5%		13.0%		9.2%		7.1%		4.9%		36.6%	37.6%
	5–14	4393	38.9%		10.4%		7.5%		5.9%		3.5%		26.8%	33.6%
	15–24	3243	42.2%	28.8	11.5%	12.0	6.9%	10.1	5.0%	10.8	2.7%	18.0	27.2%	23.7%
	25+	536	43.7%	<0.001	13.6%	0.0	7.5%	0.0	5.6%	0.0	3.4%	0.0	31.2%	24.7%
First cigarette upon awakening	≤5 min	1793	45.5%		13.5%		8.4%		6.6%		4.3%		29.7%	31.8%
	5–30 min	3405	41.0%	52.1	11.4%	9.7	7.4%	1.8	5.7%	2.7	3.1%	4.6	27.8%	27.6%
	>30 min	5147	36.3%	<0.001	10.8%	0.0	7.6%	0.4	5.6%	0.3	3.5%	0.1	29.7%	32.8%
Smokeless tobacco use§	Yes	1402	40.2%	2386.96	8.8%	30.88	6.5%	20.41	4.3%	8.48	1.3%	0.23	-	-
	No	86636	6.5%	<0.001	1.9%	<0.001	1.4%	<0.001	1.1%	0.0	0.8%	0.6	-	-
State level cigarette tax ranking†	Lowest quarter	18613	8.0%		2.5%		1.8%		1.5%		1.1%		31.5%	42.0%
	Med-low quarter	25948	7.0%		1.9%		1.5%		1.2%		0.9%		27.6%	46.8%
	Med-high quarter	22165	7.2%	54.5	1.9%	52.7	1.3%	41.7	1.0%	33.4	0.6%	34.7	26.1%	34.1%
	Highest quarter	21312	6.2%	<0.001	1.5%	<0.001	1.1%	<0.001	0.9%	<0.001	0.6%	<0.001	25.0%	40.9%
Tobacco control spending per capita†	Lowest quarter	22725	7.3%		2.2%		1.6%		1.3%		0.9%		30.1%	42.1%
	Med-low quarter	22645	7.4%		2.0%		1.5%		1.2%		0.9%		27.5%	43.0%
	Med-high quarter	27398	6.0%	71.5	1.6%	26.6	1.1%	28.9	0.9%	26.5	0.6%	15.8	26.6%	39.5%
	Highest quarter	15270	8.0%	<0.001	2.1%	<0.001	1.5%	<0.001	1.2%	<0.001	0.8%	0.001	25.7%	39.8%
State level worksite smoking ban	Highest level	54635	7.0%	0.0	1.9%	5.1	1.3%	6.6	1.1%	7.2	0.7%	10.8	26.5%	38.9%
	Not highest level	33403	7.1%	0.849	2.1%	0.024	1.5%	0.010	1.3%	0.007	0.9%	0.001	29.4%	44.7%
State level worksite e-cigarette ban	Highest level	3852	5.6%	13.6	1.5%	4.5	1.0%	3.9	0.9%	2.8	0.5%	3.3	26.6%	36.8%
	Not highest level	84186	7.1%	<0.001	2.0%	0.033	1.4%	0.048	1.1%	0.096	0.8%	0.068	27.6%	41.4%
State level e-cigarette tax	yes	1624	7.7%	1.1	1.8%	0.2	1.3%	0.1	1.0%	0.1	1.0%	0.7	23.2%	55.2%
	no	86414	7.0%	0.297	1.9%	0.642	1.4%	0.699	1.1%	0.736	0.8%	0.403	27.7%	41.0%
Census division	East North Central	10026	8.0%		2.1%		1.6%		1.2%		0.8%		26.5%	39.9%
	East South Central	6168	8.4%		2.6%		2.0%		1.8%		1.2%		30.9%	46.0%
	Middle Atlantic	7430	5.9%		1.5%		1.0%		0.9%		0.5%		25.4%	36.0%
	Mountain	10214	8.1%		2.4%		1.7%		1.4%		1.0%		29.6%	41.8%
	New England	7821	5.8%		1.6%		1.0%		0.9%		0.6%		27.8%	38.6%
	Pacific	11521	5.8%		1.3%		1.0%		0.8%		0.6%		22.8%	45.8%
	South Atlantic	16760	6.1%		1.8%		1.2%		1.0%		0.7%		29.5%	41.5%
	West North Central	9041	8.3%	167.1	2.3%	71.9	1.6%	63.6	1.2%	55.0	0.8%	38.0	27.7%	34.3%
	West South Central	9057	8.0%	<0.001	2.2%	<0.001	1.7%	<0.001	1.3%	<0.001	1.0%	<0.001	27.0%	46.4%

§ Sample size for smokeless tobacco use was in terms of ever use. For ever use, current use, 5 more days, 10 more days and 20 more days in last month, the number of respondents who use SLT with the same measure of e-cigarette are 1402, 125, 107, 92, and 78. The prevalence of e-cigarette use by five measures was estimated among SLT users who were categorized by the same measure; † Data for state level cigarette tax ranking are in Jan 2015 and tobacco control spending per capita are in 2015.

Supplementary Table S2a. Logistic Regression Equations, Male Smokers 12 Months Ago, Ever, Current (at Least Once in the Last 30 Days), 5 or More, 10 or More, 20 or More of the Last 30 Days Measures of E-cigarette Use, TUS-CPS, 2014/5

Variable	Category	Ever use			Current use			>5 days			>10 days			>20 days		
		OR	Prob Δ	95% CI	OR	Prob	95% CI	OR	Prob	95% CI	OR	Prob	95% CI	OR	Prob	95% CI
Intercept		0.18	***	0.12–0.27	0.06	***	0.03–0.11	0.05	***	0.02–0.09	0.03	***	0.02–0.07	0.03	***	0.01–0.06
Age (ref: 35–44)	18–24	2.02	***	1.61–2.52	1.24		0.91–1.70	1.24		0.88–1.75	1.30		0.90–1.87	1.43	*	0.95–2.15
	22–25	1.53	***	1.30–1.79	1.19		0.95–1.50	1.03		0.80–1.33	0.99		0.75–1.30	1.09		0.79–1.49
	45–64	0.67	***	0.58–0.77	0.72	***	0.58–0.88	0.66	***	0.53–0.83	0.66	***	0.52–0.85	0.64	***	0.48–0.85
	65+	0.44	***	0.36–0.55	0.48	***	0.34–0.67	0.50	***	0.34–0.72	0.51	***	0.34–0.75	0.49	***	0.31–0.79
Race (ref: White)	Asian	0.68	**	0.49–0.95	0.42	***	0.23–0.76	0.44	**	0.22–0.86	0.52	*	0.26–1.04	0.29	***	0.11–0.72
	Black	0.81	**	0.67–0.98	0.64	***	0.46–0.89	0.55	***	0.36–0.83	0.58	**	0.37–0.91	0.63	*	0.37–1.07
	Other races	1.26		0.94–1.68	1.64	**	1.10–2.44	1.27		0.78–2.05	1.22		0.76–1.98	1.17		0.67–2.07
Hispanic (ref: Non-Hispanic)	Hispanic	0.76	***	0.61–0.94	0.57	***	0.41–0.80	0.45	***	0.30–0.69	0.44	***	0.28–0.68	0.40	***	0.24–0.67
Education (ref: Less than 12 years)	High school degree	1.16	*	0.99–1.37	1.28	*	0.99–1.67	1.28		0.94–1.73	1.41	**	1.01–1.96	1.57	**	1.07–2.31
	Associate degree	1.60	***	1.34–1.89	1.79	***	1.36–2.35	1.67	***	1.22–2.29	1.70	***	1.20–2.41	1.61	**	1.07–2.43
	College degree or higher	1.77	***	1.43–2.19	1.65	***	1.18–2.31	1.45	*	0.98–2.12	1.53	**	1.01–2.32	1.33		0.81–2.19
Family income (ref: $0–$19,999)	$20,000–$39,999	1.02		0.88–1.19	1.09		0.87–1.37	1.09		0.84–1.41	1.13		0.86–1.49	1.07		0.78–1.46
	$40,000–$74,999	0.96		0.82–1.13	0.93		0.72–1.19	0.91		0.69–1.21	0.98		0.72–1.33	0.92		0.64–1.32
	$75,000 or more	1.22	**	1.01–1.46	1.19		0.90–1.58	1.18		0.86–1.61	1.27		0.91–1.78	1.19		0.80–1.77
Marital status (ref: Married-Spouse Present)	Never Married	0.98		0.85–1.13	0.89		0.73–1.08	0.94		0.75–1.18	0.89		0.70–1.14	0.84		0.64–1.11
	Married-Spouse Absent or Widowed/Divorced/Separated	1.05		0.92–1.20	0.92		0.76–1.12	0.95		0.77–1.18	0.99		0.78–1.25	0.99		0.75–1.30
Employment status (ref: Employed)	Not in labor force or unemployed	0.96		0.83–1.11	1.14		0.91–1.43	1.05		0.81–1.34	1.07		0.81–1.40	1.04		0.75–1.44
Metropolitan status (ref: Non-MSA)	MSA	1.19	***	1.05–1.36	1.15		0.94–1.40	1.13		0.90–1.41	1.01		0.80–1.29	0.99		0.75–1.31
Indoor workers (ref: No)	Yes	1.04		0.91–1.18	1.33	***	1.10–1.61	1.26	**	1.01–1.56	1.27	**	1.01–1.60	1.25		0.96–1.64
Current smoking status (ref: Current Smokers)	Ex-Smokers (<1 year)	1.46	***	1.23–1.72	2.34	***	1.90–2.89	3.20	***	2.57–3.98	4.16	***	3.33–5.21	5.82	***	4.53–7.48
Smoking frequency 12 months ago (ref: Everyday smokers)	Someday smokers	1.31	**	1.06–1.62	1.36	*	0.98–1.89	1.33		0.90–1.96	1.43	*	0.96–2.14	1.19		0.76–1.85
Cigarette per day 12 months ago (ref: 1–4)	5–14	1.63	***	1.31–2.02	1.41	*	1.00–2.00	1.29		0.86–1.93	1.46	*	0.96–2.23	1.26		0.79–1.99
	15–24	1.83	***	1.44–2.32	1.84	***	1.28–2.65	1.66	**	1.09–2.52	1.84	***	1.18–2.88	1.67	**	1.03–2.73
	25+	2.42	***	1.83–3.20	2.69	***	1.77–4.07	2.62	***	1.63–4.21	2.75	***	1.66–4.56	2.51	***	1.43–4.41

First cigarette upon awakening § (ref: >30 min)	≤5 min	1.22	**	1.03–1.43	0.98		0.77–1.24	0.87		0.66–1.14	0.95		0.71–1.27	1.05		0.74–1.47
	5–30 min	1.17	**	1.03–1.33	1.02		0.84–1.23	0.90		0.72–1.12	0.91		0.72–1.15	0.95		0.72–1.26
Smokeless tobacco use † (ref: No)	Yes	3.79	***	3.35–4.27	1.47	**	1.03–2.10	0.98		0.57–1.68	0.69		0.36–1.32	0.49	*	0.22–1.09
State level cigarette tax ranking ‡ (ref: Lowest quarter)	Med-low quarter	1.08		0.89–1.31	0.88		0.67–1.17	1.02		0.75–1.40	1.06		0.75–1.49	0.98		0.65–1.47
	Med-high quarter	1.17		0.96–1.44	0.86		0.63–1.16	0.88		0.62–1.24	0.85		0.59–1.22	0.98		0.64–1.50
	Highest quarter	1.03		0.80–1.33	0.83		0.56–1.23	0.98		0.62–1.56	0.88		0.54–1.44	1.01		0.57–1.78
Tobacco control spending per capita ‡ (ref: Lowest quarter)	Med-low quarter	1.01		0.86–1.19	0.96		0.76–1.23	0.98		0.74–1.29	0.95		0.71–1.27	1.06		0.75–1.50
	Med-high quarter	1.02		0.86–1.21	0.98		0.77–1.25	1.01		0.77–1.34	1.09		0.81–1.47	1.27		0.89–1.81
	Highest quarter	0.83	**	0.69–1.00	0.67	***	0.51–0.88	0.68	**	0.50–0.93	0.69	**	0.49–0.96	0.76		0.50–1.13
State level worksite smoking ban (ref: Not highest level)	Highest level	1.06		0.90–1.25	1.02		0.80–1.30	0.99		0.75–1.30	1.02		0.75–1.37	1.04		0.73–1.50
State level worksite e-cigarette ban (ref: Not highest level)	Highest level	0.81		0.58–1.12	1.19		0.76–1.87	1.17		0.69–1.99	1.42		0.81–2.48	1.09		0.53–2.24
State level e-cigarette tax (ref: No)	Yes	1.12		0.73–1.71	0.93		0.49–1.75	0.98		0.49–1.99	1.11		0.53–2.29	0.97		0.42–2.23
Survey time (ref: July 2014)	Jan 2015	0.97		0.85–1.10	0.96		0.80–1.15	1.01		0.83–1.23	1.00		0.80–1.24	0.95		0.74–1.22
	May 2015	0.95		0.84–1.08	0.78	**	0.64–0.94	0.79	**	0.63–0.98	0.76	**	0.60–0.97	0.71	**	0.53–0.94
Census division (ref: Pacific)	East North Central	0.85		0.66–1.09	0.81		0.56–1.17	0.94		0.61–1.44	0.82		0.51–1.32	0.75		0.43–1.31
	East South Central	0.77	**	0.60–1.00	0.83		0.57–1.21	1.05		0.69–1.60	0.95		0.61–1.48	0.88		0.51–1.49
	Middle Atlantic	0.82		0.63–1.07	0.90		0.59–1.35	1.06		0.66–1.70	0.97		0.58–1.63	0.87		0.48–1.59
	Mountain	1.00		0.78–1.30	1.39	*	0.96–2.02	1.42		0.92–2.19	1.27		0.80–2.03	1.23		0.71–2.15
	New England	0.87		0.64–1.17	0.82		0.52–1.28	0.85		0.51–1.44	0.88		0.50–1.55	0.70		0.36–1.36
	South Atlantic	0.87		0.70–1.10	0.82		0.58–1.16	0.94		0.63–1.40	0.89		0.58–1.36	0.88		0.53–1.45
	West North Central	0.88		0.67–1.16	0.91		0.62–1.35	1.10		0.70–1.72	1.03		0.63–1.67	0.98		0.55–1.74
	West South Central	0.67	***	0.53–0.86	0.94		0.65–1.34	1.27		0.84–1.92	1.15		0.73–1.80	1.04		0.61–1.77

Δ Odds ratio and significance level of *p*-value: "***" < 0.01; 0.01 < "**" < 0.05; 0.05 < "*" <=0.1; § The time reported for current smokers was their current habit while for quitters in last year was their habit one year ago; † Smokeless tobacco use and e-cigarette use were using the same measure in each model; ‡ Data for state level cigarette tax ranking are in Jan 2015 and tobacco control spending per capita are in 2015.

Supplementary Table S2b. Logistic Regression Equations, Female Smokers 12 Months Ago, Ever, Current (at Least Once in the Last 30 Days), 5 or More, 10 or More, 20 or More of the Last 30 Days Measures of E-cigarette Use, TUS-CPS, 2014/5

Variable	Category	Ever use			Current use			>5 days			>10 days			>20 days		
		OR	ProbΔ	95% CI	OR	Prob	95% CI	OR	Prob	95% CI	OR	Prob	95% CI	OR	Prob	95% CI
Intercept		0.48	***	0.32–0.71	0.08	***	0.04–0.15	0.04	***	0.02–0.08	0.02	***	0.01–0.05	0.02	***	0.01–0.04
Age (ref: 35–44)	18–24	1.79	***	1.41–2.26	1.47	**	1.07–2.02	1.54	**	1.07–2.22	1.64	**	1.08–2.48	1.18		0.69–2.02
	22–25	1.17	**	1.01–1.37	1.03		0.82–1.29	1.07		0.82–1.40	1.07		0.79–1.45	1.15		0.80–1.64
	45–64	0.80	***	0.70–0.91	0.93		0.77–1.11	0.94		0.76–1.16	1.04		0.82–1.33	1.13		0.84–1.50
	65+	0.47	***	0.39–0.57	0.67	***	0.51–0.88	0.76	*	0.56–1.04	0.82		0.58–1.16	0.85		0.56–1.28
Race (ref: White)	Asian	0.72		0.45–1.15	1.07		0.50–2.32	1.43		0.62–3.28	1.80		0.77–4.18	1.42		0.43–4.69
	Black	0.52	***	0.43–0.63	0.47	***	0.34–0.64	0.39	***	0.25–0.58	0.36	***	0.23–0.56	0.36	***	0.21–0.62
	Other races	1.28	*	0.99–1.66	1.29		0.90–1.86	1.24		0.82–1.87	1.34		0.86–2.08	1.48		0.90–2.45
Hispanic (ref: Non-Hispanic)	Hispanic	0.70	***	0.55–0.88	0.68	**	0.47–0.99	0.65	*	0.41–1.03	0.63	*	0.39–1.01	0.58	*	0.33–1.02
Education (ref: Less than 12 years)	High school degree	1.20	**	1.02–1.40	1.18		0.93–1.49	1.08		0.82–1.42	1.30		0.95–1.79	1.18		0.81–1.72
	Associate degree	1.43	***	1.22–1.69	1.27	**	1.00–1.61	1.16		0.88–1.52	1.34	*	0.97–1.84	1.31		0.89–1.93
	College degree or higher	1.36	***	1.11–1.67	1.24		0.91–1.69	1.00		0.70–1.44	1.13		0.75–1.72	1.21		0.74–1.99
Family income (ref: $0–$19,999)	$20,000–$39,999	1.02		0.89–1.17	0.89		0.73–1.09	0.86		0.68–1.09	0.83		0.64–1.07	0.86		0.62–1.18
	$40,000–$74,999	1.09		0.94–1.26	0.88		0.71–1.10	0.84		0.65–1.09	0.82		0.61–1.10	0.91		0.64–1.29
	$75,000 or more	1.15		0.96–1.36	0.85		0.66–1.09	0.84		0.62–1.13	0.90		0.65–1.26	0.95		0.65–1.40
Marital status (ref: Married-Spouse Present)	Never Married	0.90		0.78–1.04	0.86		0.69–1.07	0.91		0.71–1.17	0.89		0.66–1.19	0.94		0.65–1.36
	Married-Spouse Absent or Widowed/Divorced/Separated	1.04		0.93–1.18	1.02		0.86–1.21	1.13		0.93–1.38	1.20		0.95–1.50	1.22		0.94–1.59
Employment status (ref: Employed)	Not in labor force or unemployed	0.95		0.80–1.12	1.15		0.90–1.46	1.17		0.87–1.57	1.01		0.74–1.39	0.91		0.64–1.30
Metropolitan status (ref: Non-MSA)	MSA	1.21	***	1.07–1.36	1.14		0.96–1.36	1.17		0.95–1.44	1.22		0.95–1.55	1.21		0.92–1.59
Indoor workers (ref: No)	Yes	0.88		0.74–1.03	0.97		0.76–1.24	1.10		0.82–1.47	1.03		0.76–1.41	0.87		0.61–1.23
Current smoking status (ref: Current Smokers)	Ex-Smokers (<1 year)	1.30	***	1.11–1.52	1.53	***	1.24–1.88	2.17	***	1.73–2.72	2.78	***	2.20–3.52	4.02	***	3.09–5.22
Smoking frequency 12 months ago (ref: Everyday smokers)	Someday smokers	1.23	**	1.01–1.49	1.57	***	1.17–2.11	1.68	***	1.18–2.39	1.69	**	1.13–2.54	1.48		0.90–2.42
	5–14	1.37	***	1.13–1.66	1.70	***	1.26–2.31	2.05	***	1.42–2.97	2.16	***	1.41–3.31	2.17	***	1.29–3.63
	15–24	1.56	***	1.26–1.92	2.11	***	1.52–2.93	2.41	***	1.62–3.58	2.65	***	1.68–4.17	2.53	***	1.45–4.40

Cigarette per day 12 months ago (ref: 1–4)	25+	2.12	***	1.62–2.77	2.69	***	1.81–4.00	3.04	***	1.89–4.88	3.17	***	1.85–5.42	2.83	***	1.49–5.38
First cigarette upon awakening § (ref: >30 min)	≤5 min	1.30	***	1.13–1.51	1.18		0.96–1.44	1.01		0.80–1.28	1.08		0.83–1.40	1.15		0.85–1.55
	5–30 min	1.18	***	1.05–1.32	0.93		0.78–1.10	0.89		0.73–1.08	0.86		0.69–1.06	0.75	**	0.57–0.98
Smokeless tobacco use † (ref: No)	Yes	4.54	***	3.43–6.01	5.34	***	1.96–14.49	11.02	***	2.63–46.15	12.21	***	2.02–73.89	1.46		0.08–28.28
State level cigarette tax ranking ‡ (ref: Lowest quarter)	Med-low quarter	0.99		0.82–1.18	1.02		0.78–1.32	1.22		0.90–1.66	1.45	**	1.03–2.06	1.71	**	1.12–2.60
	Med-high quarter	1.06		0.88–1.28	0.97		0.73–1.27	1.07		0.77–1.49	1.23		0.84–1.80	1.13		0.70–1.81
	Highest quarter	1.00		0.79–1.26	0.82		0.57–1.18	0.97		0.62–1.52	1.22		0.74–2.01	1.10		0.59–2.03
Tobacco control spending per capita ‡ (ref: Lowest quarter)	Med-low quarter	0.82	***	0.71–0.95	0.77	**	0.62–0.95	0.85		0.66–1.10	0.86		0.65–1.14	0.98		0.71–1.36
	Med-high quarter	0.78	***	0.67–0.91	0.83		0.65–1.04	0.86		0.66–1.13	0.74	*	0.55–1.00	0.75		0.51–1.08
	Highest quarter	0.82	**	0.69–0.98	0.70	***	0.54–0.92	0.77	*	0.57–1.04	0.72	*	0.52–1.00	0.69	*	0.46–1.04
State level worksite smoking ban (ref: Not highest level)	Highest level	0.99		0.85–1.15	1.03		0.81–1.30	0.86		0.66–1.13	0.76	*	0.56–1.02	0.79		0.56–1.11
State level worksite e-cigarette ban (ref: Not highest level)	Highest level	0.81		0.59–1.12	0.90		0.55–1.47	0.89		0.49–1.61	0.79		0.40–1.56	0.91		0.38–2.17
State level e-cigarette tax (ref: No)	Yes	1.21		0.81–1.80	0.69		0.36–1.34	0.54		0.25–1.18	0.56		0.23–1.33	1.03		0.39–2.73
Survey time (ref: July 2014)	Jan 2015	1.03		0.92–1.16	0.96		0.81–1.13	0.94		0.77–1.15	0.97		0.77–1.22	0.86		0.66–1.12
	May 2015	0.90	*	0.80–1.02	0.78	***	0.65–0.93	0.85		0.69–1.04	0.94		0.75–1.18	0.93		0.71–1.21
Census division (ref: Pacific)	East North Central	0.83		0.65–1.05	1.05		0.73–1.51	1.12		0.73–1.71	0.97		0.61–1.54	0.91		0.53–1.56
	East South Central	0.86		0.68–1.09	0.99		0.70–1.42	1.21		0.81–1.82	1.18		0.76–1.84	1.08		0.65–1.80
	Middle Atlantic	0.94		0.72–1.22	1.07		0.71–1.61	1.18		0.73–1.93	1.10		0.63–1.92	1.05		0.54–2.04
	Mountain	1.06		0.82–1.36	1.21		0.83–1.76	1.32		0.85–2.05	1.29		0.80–2.08	1.18		0.68–2.07
	New England	0.69	**	0.52–0.92	1.10		0.72–1.69	1.16		0.70–1.94	1.07		0.62–1.86	1.15		0.59–2.24
	South Atlantic	0.86		0.69–1.07	1.04		0.75–1.45	1.16		0.79–1.72	1.14		0.75–1.75	1.28		0.78–2.08
	West North Central	0.81		0.63–1.05	1.05		0.72–1.53	1.29		0.83–2.01	1.19		0.74–1.94	0.97		0.54–1.72
	West South Central	0.84		0.66–1.06	0.97		0.68–1.37	1.05		0.69–1.58	0.90		0.57–1.42	1.08		0.63–1.84

Δ Odds ratio and significance level of *p*-value: "***" < 0.01; 0.01 < "**" < 0.05; 0.05 < "*" <=0.1; § The time reported for current smokers was their current habit while for quitters in last year was their habit one year ago; † Smokeless tobacco use and e-cigarette use were using the same measure in each model; ‡ Data for state level cigarette tax ranking are in Jan 2015 and tobacco control spending per capita are in 2015.

Supplementary Table S3a. Logistic Regression Equations, Male Former Smokers Who Quit More Than One Year and Less Than or Equal to Three Years, Ever, Current (at Least Once in the Last 30 Days), 5 or More, 10 or More, 20 or More of the Last 30 Days Measures of E-cigarette Use, TUS-CPS, 2014/5

Variable	Category	Ever use			Current use			>5 days			>10 days			>20 days		
		OR	ProbΔ	95% CI	OR	Prob	95% CI	OR	Prob	95% CI	OR	Prob	95% CI	OR	Prob	95% CI
Intercept		0.06	***	0.02–0.18	0.02	***	0.00–0.12	0.02	***	0.00–0.09	0.02	***	0.00–0.09	0.01	***	0.00–0.08
Age (ref: 35–44)	18–24	1.73	*	0.92–3.24	0.97		0.38–2.51	0.90		0.35–2.34	0.89		0.34–2.32	0.57		0.17–1.91
	22–25	1.13		0.76–1.69	0.58	*	0.32–1.07	0.53	*	0.28–1.01	0.51	**	0.26–0.98	0.60		0.30–1.19
	45–64	0.93		0.61–1.41	0.75		0.41–1.36	0.75		0.41–1.39	0.81		0.43–1.51	0.91		0.47–1.75
	65+	0.41	***	0.21–0.79	0.61		0.23–1.67	0.65		0.24–1.80	0.68		0.24–1.92	0.67		0.22–2.09
Race (ref: White)	Asian	1.03		0.48–2.22	2.09		0.73–5.98	1.47		0.42–5.16	1.48		0.42–5.22	1.34		0.32–5.54
	Black	0.55		0.27–1.13	0.13	*	0.02–1.02	0.14	*	0.02–1.07	0.14	*	0.02–1.10	0.18	*	0.02–1.35
	Other races	0.55		0.25–1.24	0.68		0.18–2.54	0.78		0.20–3.00	0.80		0.21–3.06	0.49		0.12–1.99
Hispanic (ref: Non–Hispanic)	Hispanic	0.78		0.45–1.36	0.24	**	0.07–0.85	0.10	***	0.02–0.51	0.05	***	0.01–0.43	0.06	**	0.01–0.58
Education (ref: Less than 12 years)	High school degree	1.73	*	0.92–3.25	1.47		0.54–3.97	1.25		0.47–3.35	1.26		0.47–3.40	1.55		0.54–4.40
	Associate degree	1.94	**	1.05–3.59	1.82		0.67–4.93	1.40		0.53–3.72	1.30		0.49–3.49	1.40		0.50–3.93
	College degree or higher	1.89	*	0.98–3.65	0.72		0.24–2.19	0.62		0.21–1.86	0.61		0.20–1.85	0.76		0.23–2.46
Family income (ref: $0–$19,999)	$20,000–$39,999	2.59	***	1.45–4.64	2.00		0.75–5.32	2.81	*	0.98–8.08	2.92	*	0.99–8.55	4.19	**	1.23–14.30
	$40,000–$74,999	1.78	*	0.98–3.23	1.14		0.41–3.16	1.58		0.51–4.86	1.62		0.51–5.09	2.38		0.64–8.88
	$75,000 or more	1.72	*	0.91–3.23	1.50		0.51–4.39	2.04		0.61–6.83	1.82		0.53–6.20	2.46		0.60–10.09
Marital status (ref: Married-Spouse Present)	Never Married	0.95		0.66–1.37	0.55	**	0.31–0.96	0.47	**	0.26–0.86	0.48	**	0.26–0.88	0.47	**	0.24–0.92
	Married-Spouse Absent or Widowed/Divorced/Separated	1.28		0.86–1.90	0.70		0.38–1.28	0.73		0.39–1.34	0.68		0.37–1.24	0.77		0.40–1.45
Employment status (ref: Employed)	Not in labor force or unemployed	1.12		0.70–1.79	0.74		0.33–1.66	0.69		0.29–1.62	0.74		0.31–1.79	0.83		0.31–2.22
Metropolitan status (ref: Non-MSA)	MSA	1.52	**	1.02–2.25	1.90	**	1.04–3.49	1.79	*	0.97–3.31	1.84	*	0.98–3.42	1.46		0.76–2.80
Indoor workers (ref: No)	Yes	1.46	**	1.03–2.08	1.36		0.79–2.35	1.39		0.79–2.45	1.55		0.87–2.76	2.03	**	1.08–3.80

Smokeless tobacco use § (ref: No)	Yes	2.52	***	1.84–3.44	0.50		0.19–1.37	0.38		0.11–1.25	0.42		0.13–1.40	0.42		0.11–1.55
State level cigarette tax ranking † (ref: Lowest quarter)	Med-low quarter	1.08		0.59–1.97	0.85		0.33–2.21	0.84		0.31–2.24	0.83		0.31–2.24	0.76		0.27–2.13
	Med-high quarter	1.33		0.73–2.41	1.85		0.77–4.44	2.03		0.84–4.90	2.07		0.84–5.06	2.08		0.82–5.24
	Highest quarter	1.88	*	0.91–3.87	1.95		0.61–6.24	1.90		0.58–6.25	2.16		0.65–7.23	2.37		0.65–8.56
Tobacco control spending per capita † (ref: Lowest quarter)	Med-low quarter	0.99		0.63–1.56	0.94		0.45–1.97	0.91		0.44–1.87	0.97		0.47–2.00	1.03		0.47–2.24
	Med-high quarter	1.13		0.69–1.84	1.63		0.77–3.45	1.46		0.67–3.15	1.44		0.66–3.10	1.17		0.53–2.61
	Highest quarter	1.07		0.62–1.86	1.37		0.58–3.22	1.31		0.54–3.21	1.34		0.56–3.22	1.14		0.44–2.92
State level worksite smoking ban (ref: Not highest level)	Highest level	0.87		0.54–1.40	1.06		0.50–2.22	1.29		0.58–2.86	1.16		0.52–2.55	1.16		0.50–2.71
State level worksite e-cigarette ban (ref: Not highest level)	Highest level	0.99		0.45–2.15	0.91		0.29–2.80	0.81		0.25–2.64	0.79		0.24–2.62	1.00		0.28–3.52
State level e-cigarette tax (ref: No)	Yes	0.88		0.30–2.56	1.03		0.18–5.81	1.04		0.18–6.13	1.01		0.17–6.10	0.56		0.08–3.97
Survey time (ref: July 2014)	Jan 2015	1.32		0.93–1.87	1.61	*	0.92–2.80	1.53		0.87–2.67	1.71	*	0.96–3.04	1.73	*	0.92–3.22
	May 2015	1.46	**	1.02–2.07	1.45		0.82–2.55	1.64	*	0.94–2.85	1.76	*	0.99–3.13	1.66		0.89–3.08
Census division (ref: Pacific)	East North Central	0.53	*	0.27–1.07	1.06		0.35–3.17	1.10		0.35–3.52	0.99		0.31–3.16	0.60		0.18–2.01
	East South Central	0.91		0.44–1.88	2.51	*	0.86–7.34	3.45	**	1.09–10.96	3.32	**	1.04–10.58	3.29	**	1.01–10.72
	Middle Atlantic	0.37	***	0.18–0.76	0.62		0.21–1.84	0.80		0.25–2.56	0.80		0.25–2.56	0.46		0.13–1.62
	Mountain	0.97		0.47–1.99	1.69		0.58–4.93	1.96		0.61–6.30	2.09		0.65–6.67	2.46		0.72–8.38
	New England	0.31	***	0.14-0.71	0.52		0.13-2.11	0.63		0.14-2.77	0.57		0.13-2.51	0.45		0.10-2.11
	South Atlantic	0.87		0.47-1.61	1.94		0.76-4.97	2.62	*	0.94-7.29	2.34		0.83-6.58	1.91		0.64-5.75
	West North Central	0.74		0.35-1.57	0.71		0.20-2.60	0.91		0.23-3.55	0.93		0.24-3.63	0.64		0.16-2.59
	West South Central	0.76		0.39-1.47	1.56		0.58-4.16	2.08		0.72-5.94	2.12		0.74-6.04	2.02		0.67-6.09

Δ Odds ratio and significance level of *p*-value: "***" < 0.01; 0.01 < "**" < 0.05; 0.05 < "*" <=0.1; § Smokeless tobacco use and e–cigarette use were using the same measure in each model; ** Data for state level cigarette tax ranking are in Jan 2015 and tobacco control spending per capita are in 2015.

Supplementary Table S3b. Logistic Regression Equations, Female Former Smokers Who Quit More Than One Year and Less Than or Equal to Three Years, Ever, Current (at Least Once in the Last 30 Days), 5 or More, 10 or More, 20 or More of the Last 30 Days Measures of E-cigarette Use, TUS-CPS, 2014/5

Variable	Category	Ever use			Current use			>5 days			>10 days			>20 days		
		OR	ProbΔ	95% CI	OR	Prob	95% CI	OR	Prob	95% CI	OR	Prob	95% CI	OR	Prob	95% CI
Intercept		0.20	***	0.06–0.64	0.05	***	0.01–0.30	0.05	***	0.01–0.36	0.05	***	0.01–0.36	0.05	***	0.01–0.45
Age (ref: 35–44)	18–24	1.20		0.61–2.38	0.16	**	0.04–0.68	0.08	***	0.01–0.46	0.08	***	0.01–0.49	0.03	***	0.00–0.23
	22–25	1.06		0.68–1.65	0.74		0.37–1.49	0.72		0.34–1.51	0.71		0.33–1.53	0.71		0.31–1.63
	45–64	1.06		0.70–1.63	1.11		0.62–1.99	1.13		0.61–2.08	1.28		0.68–2.41	1.54		0.77–3.06
	65+	0.82		0.44–1.54	0.75		0.31–1.82	0.76		0.30–1.94	0.77		0.29–2.05	1.01		0.36–2.84
Race (ref: White)	Asian	0.64		0.18–2.35	0.13	*	0.01–1.14	0.15	*	0.02–1.37	0.20		0.02–1.75	0.26		0.03–2.28
	Black	0.62		0.33–1.17	0.47		0.17–1.30	0.50		0.17–1.41	0.57		0.20–1.61	0.57		0.19–1.74
	Other races	2.75	***	1.41–5.34	2.45	*	0.97–6.18	2.81	**	1.09–7.22	3.12	**	1.26–7.74	3.77	***	1.46–9.72
Hispanic (ref: Non-Hispanic)	Hispanic	0.45	**	0.23–0.88	0.39		0.11–1.33	0.45		0.13–1.56	0.53		0.15–1.87	0.70		0.20–2.42
Education (ref: Less than 12 years)	High school degree	1.27		0.69–2.34	0.95		0.41–2.21	1.14		0.46–2.86	1.12		0.44–2.83	1.36		0.49–3.73
	Associate degree	1.30		0.70–2.42	1.40		0.60–3.28	1.62		0.64–4.09	1.43		0.56–3.69	1.59		0.57–4.45
	College degree or higher	0.99		0.52–1.89	0.63		0.25–1.60	0.72		0.27–1.96	0.65		0.23–1.84	0.70		0.23–2.17
Family income (ref: $0–$19,999)	$20,000–$39,999	1.01		0.63–1.61	1.26		0.63–2.51	1.18		0.58–2.43	1.23		0.60–2.51	1.09		0.51–2.34
	$40,000–$74,999	1.29		0.80–2.08	1.49		0.75–2.97	1.24		0.60–2.55	1.25		0.59–2.64	1.25		0.57–2.73
	$75,000 or more	0.87		0.52–1.47	1.61		0.76–3.44	1.47		0.67–3.22	1.46		0.65–3.26	1.38		0.58–3.26
Marital status (ref: Married-Spouse Present)	Never Married	1.20		0.80–1.80	0.86		0.42–1.73	0.71		0.32–1.55	0.77		0.35–1.70	0.90		0.40–2.01
	Married-Spouse Absent or Widowed/Divorced/Separated	1.06		0.74–1.53	1.28		0.75–2.20	1.20		0.69–2.09	1.06		0.60–1.88	0.86		0.47–1.57
Employment status (ref: Employed)	Not in labor force or unemployed	0.83		0.50–1.40	1.68		0.80–3.51	1.40		0.65–2.99	1.22		0.56–2.63	1.11		0.49–2.49
Metropolitan status (ref: Non-MSA)	MSA	1.21		0.83–1.75	1.78	*	0.99–3.17	1.98	**	1.07–3.66	1.71	*	0.94–3.11	1.73	*	0.90–3.31
Indoor workers (ref: No)	Yes	1.26		0.79–2.02	0.95		0.44–2.05	0.84		0.39–1.82	0.81		0.37–1.78	0.77		0.33–1.80
Smokeless tobacco use § (ref: No)	Yes	6.12	***	2.79–13.41	0.00	***	0.00–0.00	0.00	***	0.00–0.00	0.00	***	0.00–0.00	0.00	***	0.00–0.00
State level cigarette tax ranking † (ref: Lowest quarter)	Med-low quarter	1.03		0.59–1.81	0.95		0.47–1.95	1.02		0.48–2.16	0.78		0.36–1.69	0.83		0.35–1.99
	Med-high quarter	1.35		0.76–2.40	1.12		0.50–2.48	1.00		0.42–2.40	1.07		0.45–2.52	0.83		0.30–2.29
	Highest quarter	1.92	*	0.92–3.99	1.17		0.36–3.78	1.11		0.31–3.97	1.17		0.34–4.00	0.98		0.24–4.02
Tobacco control spending per capita † (ref: Lowest quarter)	Med-low quarter	1.37		0.87–2.15	1.87	*	0.99–3.55	1.57		0.80–3.08	1.46		0.75–2.84	1.36		0.67–2.74
	Med-high quarter	1.06		0.65–1.72	0.51	*	0.26–1.01	0.44	**	0.21–0.90	0.45	**	0.22–0.91	0.38	**	0.17–0.82
	Highest quarter	1.11		0.65–1.90	1.39		0.65–2.99	1.27		0.58–2.76	1.16		0.55–2.42	1.18		0.54–2.61

State level worksite smoking ban (ref: Not highest level)	Highest level	0.58	**	0.36–0.92	0.41	***	0.21–0.78	0.43	**	0.22–0.83	0.54	*	0.27–1.08	0.52	*	0.24–1.08
State level worksite e-cigarette ban (ref: Not highest level)	Highest level	1.26		0.60–2.65	2.22	*	0.86–5.73	2.45	*	0.89–6.79	2.33	*	0.88–6.17	2.30		0.74–7.09
State level e-cigarette tax (ref: No)	Yes	1.01		0.28–3.59	9.72	***	1.78–52.99	5.73	**	1.01–32.45	3.96		0.74–21.14	6.20	**	1.03–37.38
Survey time (ref: July 2014)	Jan 2015	1.03		0.72–1.46	0.85		0.50–1.43	0.86		0.49–1.49	1.01		0.56–1.80	0.97		0.52–1.82
	May 2015	1.25		0.88–1.79	1.24		0.73–2.11	1.37		0.79–2.37	1.67	*	0.94–2.96	1.56		0.85–2.89
Census division (ref: Pacific)	East North Central	1.18		0.56–2.48	0.80		0.24–2.66	0.86		0.26–2.88	0.66		0.19–2.23	0.67		0.18–2.45
	East South Central	0.99		0.44–2.23	1.43		0.42–4.86	1.45		0.41–5.06	1.72		0.47–6.27	1.43		0.35–5.79
	Middle Atlantic	0.76		0.34–1.67	1.42		0.42–4.83	1.37		0.38–4.91	1.22		0.35–4.28	1.33		0.34–5.18
	Mountain	2.52	***	1.26–5.05	3.00	**	1.06–8.50	2.34		0.81–6.76	2.20		0.74–6.57	1.74		0.55–5.47
	New England	0.50	*	0.22–1.13	1.04		0.29–3.73	0.94		0.25–3.59	0.90		0.24–3.37	1.05		0.26–4.22
	South Atlantic	1.12		0.58–2.16	0.98		0.29–3.27	0.87		0.24–3.10	0.94		0.26–3.45	0.82		0.19–3.48
	West North Central	1.13		0.53–2.39	1.97		0.61–6.34	1.91		0.58–6.30	2.09		0.63–6.90	1.68		0.47–6.05
	West South Central	1.72		0.87–3.40	1.31		0.45–3.82	1.37		0.46–4.06	1.24		0.39–3.90	0.90		0.27–3.04

Δ Odds ratio and significance level of *p*-value: "***" < 0.01; 0.01 < "**" < 0.05; 0.05 < "*" <=0.1; § Smokeless tobacco use and e-cigarette use were using the same measure in each model; † Data for state level cigarette tax ranking are in Jan 2015 and tobacco control spending per capita are in 2015.

Supplementary Table S4a. Logistic Regression Equations, Male Former Smokers Who Quit More Than Three Years, Ever, Current (at Least Once in the Last 30 Days), 5 or More, 10 or More, 20 or More of the Last 30 Days Measures of E-cigarette Use, TUS-CPS, 2014/5

Variable	Category	Ever use			Current use			>5 days			>10 days			>20 days		
		OR	ProbΔ	95% CI	OR	Prob	95% CI	OR	Prob	95% CI	OR	Prob	95% CI	OR	Prob	95% CI
Intercept		0.01	***	0.00–0.03	0.00	***	0.00–0.02	0.00	***	0.00–0.01	0.00	***	0.00–0.01	0.00	***	0.00–0.01
Age (ref: 35–44)	18–24	5.06	***	2.36–10.86	1.69		0.37–7.69	1.82		0.39–8.48	1.04		0.15–7.39	1.14		0.17–7.64
	22–25	2.73	***	1.97–3.77	1.44		0.75–2.77	1.41		0.67–2.98	1.46		0.69–3.13	1.38		0.59–3.21
	45–64	0.49	***	0.36–0.67	0.48	**	0.25–0.93	0.55	*	0.27–1.10	0.56		0.28–1.15	0.40	**	0.18–0.92
	65+	0.15	***	0.09–0.23	0.11	***	0.04–0.30	0.13	***	0.05–0.38	0.13	***	0.05–0.38	0.12	***	0.04–0.40
Race (ref: White)	Asian	0.61		0.28–1.35	0.00	***	0.00–0.00	0.00	***	0.00–0.00	0.00	***	0.00–0.00	0.00	***	0.00–0.00
	Black	0.63		0.32–1.24	0.26		0.03–2.05	0.27		0.03–2.15	0.28		0.03–2.17	0.37		0.05–3.01
	Other races	0.86		0.35–2.08	1.94		0.63–5.96	0.40		0.08–2.00	0.32		0.05–2.29	0.44		0.06–3.24
Hispanic (ref: Non–Hispanic)	Hispanic	0.80		0.50–1.28	0.79		0.30–2.07	1.30		0.56–3.03	1.15		0.48–2.76	1.14		0.42–3.12
Education (ref: Less than 12 years)	High school degree	1.44		0.83–2.48	0.66		0.22–1.96	1.88		0.57–6.25	1.86		0.56–6.23	3.10		0.71–13.59
	Associate degree	1.70	*	0.98–2.94	1.12		0.36–3.44	3.37	**	1.05–10.88	3.27	**	1.00–10.64	5.18	**	1.24–21.68
	College degree or higher	1.46		0.81–2.62	0.85		0.26–2.82	2.86	*	0.86–9.51	2.93	*	0.87–9.80	3.27		0.76–14.11

Family income (ref: $0–$19,999)	$20,000–$39,999	0.92		0.58–1.47	1.31		0.54–3.19	1.48		0.55–3.97	1.45		0.54–3.88	1.55		0.48–5.02
	$40,000–$74,999	0.80		0.50–1.27	0.99		0.40–2.47	0.97		0.35–2.64	0.90		0.33–2.43	0.91		0.26–3.18
	$75,000 or more	0.97		0.60–1.58	0.94		0.32–2.74	0.66		0.22–2.02	0.56		0.19–1.66	0.70		0.18–2.68
Marital status (ref: Married–Spouse Present)	Never Married	1.82	***	1.35–2.45	1.35		0.69–2.62	1.33		0.65–2.74	1.27		0.61–2.64	1.09		0.47–2.54
	Married-Spouse Absent or Widowed/Divorced /Separated	1.82	***	1.34–2.47	1.81	*	0.94–3.50	1.36		0.70–2.64	1.27		0.65–2.49	1.15		0.53–2.51
Employment status (ref: Employed)	Not in labor force or unemployed	0.97		0.67–1.40	0.89		0.43–1.83	1.00		0.43–2.31	0.95		0.41–2.23	0.94		0.34–2.58
Metropolitan status (ref: Non-MSA)	MSA	1.46	**	1.07–1.99	1.27		0.71–2.25	1.22		0.63–2.33	1.19		0.62–2.30	0.83		0.40–1.75
Indoor workers (ref: No)	Yes	1.26		0.96–1.66	1.22		0.68–2.18	1.66		0.88–3.14	1.58		0.83–3.02	1.48		0.71–3.09
Smokeless tobacco use § (ref: No)	Yes	3.44	***	2.67–4.42	1.24		0.49–3.11	0.87		0.29–2.61	0.87		0.27–2.78	0.00	***	0.00–0.00
State level cigarette tax ranking † (ref: Lowest quarter)	Med–low quarter	1.61	*	0.98–2.64	1.20		0.49–2.94	1.19		0.47–3.01	1.12		0.43–2.91	1.48		0.50–4.39
	Med-high quarter	1.54	*	0.93–2.56	1.13		0.46–2.78	0.86		0.36–2.09	0.89		0.37–2.12	0.91		0.31–2.65
	Highest quarter	2.04	**	1.11–3.74	1.96		0.53–7.22	3.45	*	0.98–12.07	3.34	*	0.96–11.64	2.77		0.65–11.78
Tobacco control spending per capita † (ref: Lowest quarter)	Med–low quarter	1.18		0.81–1.74	1.43		0.59–3.45	1.52		0.61–3.78	1.61		0.63–4.07	1.62		0.55–4.82
	Med-high quarter	1.16		0.81–1.65	1.44		0.68–3.03	1.74		0.85–3.55	1.92	*	0.93–3.96	1.23		0.50–3.03
	Highest quarter	1.02		0.66–1.57	1.23		0.48–3.13	1.11		0.36–3.44	0.99		0.28–3.51	0.88		0.22–3.43
State level worksite smoking ban (ref: Not highest level)	Highest level	0.76		0.52–1.12	0.94		0.46–1.91	0.92		0.44–1.95	0.92		0.43–1.95	0.65		0.27–1.55
State level worksite e–cigarette ban (ref: Not highest level)	Highest level	1.09		0.60–1.99	0.28		0.04–2.10	0.00	***	0.00–0.00	0.00	***	0.00–0.00	0.00	***	0.00–0.00
State level e-cigarette tax (ref: No)	Yes	0.38	*	0.13–1.12	1.23		0.20–7.49	0.39		0.06–2.74	0.44		0.06–3.12	2.00		0.20–20.14
Survey time (ref: July 2014)	Jan 2015	1.03		0.78–1.35	1.67		0.89–3.16	1.44		0.74–2.81	1.61		0.81–3.20	1.71		0.74–3.95

	May 2015	1.01		0.76–1.34	1.91	**	1.02–3.58	1.70		0.88–3.29	1.87	*	0.94–3.71	2.14	*	0.95–4.80
Census division (ref: Pacific)	East North Central	1.02		0.61–1.71	0.55		0.17–1.83	0.61		0.16–2.31	0.62		0.16–2.37	0.64		0.11–3.60
	East South Central	1.03		0.56–1.86	2.86	**	1.04–7.82	5.57	***	1.89–16.37	5.32	***	1.79–15.81	4.30	**	1.20–15.44
	Middle Atlantic	0.83		0.48–1.41	0.26		0.05–1.35	0.16	*	0.02–1.38	0.15	*	0.02–1.33	0.00	***	0.00–0.00
	Mountain	1.74	**	1.07–2.84	1.96		0.72–5.33	3.98	**	1.30–12.17	3.65	**	1.13–11.77	3.98	*	0.94–16.78
	New England	0.58	*	0.31–1.08	0.20	**	0.04–0.91	0.24	*	0.05–1.23	0.24	*	0.05–1.29	0.31		0.04–2.28
	South Atlantic	1.05		0.63–1.77	1.07		0.42–2.72	1.47		0.51–4.19	1.47		0.51–4.25	1.53		0.42–5.63
	West North Central	1.31		0.75–2.28	1.27		0.41–3.88	2.62		0.80–8.55	2.23		0.65–7.72	1.35		0.27–6.75
	West South Central	1.00		0.59–1.69	2.30		0.81–6.54	5.02	***	1.60–15.75	5.28	***	1.64–17.06	5.64	**	1.32–24.03

Δ Odds ratio and significance level of p-value: "***" < 0.01; 0.01 < "**" < 0.05; 0.05 < "*" <=0.1; § Smokeless tobacco use and e-cigarette use were using the same measure in each model; † Data for state level cigarette tax ranking are in Jan 2015 and tobacco control spending per capita are in 2015.

Supplementary Table S4b. Logistic Regression Equations, Female Former Smokers Who Quit More Than Three Years, Ever, Current (at Least Once in the Last 30 Days), 5 or More, 10 or More, 20 or More of the Last 30 Days Measures of E-cigarette Use, TUS-CPS, 2014/5

Variable	Category	Ever use			Current use			>5 days			>10 days			>20 days		
		OR	ProbΔ	95% CI	OR	Prob	95% CI	OR	Prob	95% CI	OR	Prob	95% CI	OR	Prob	95% CI
Intercept		0.02	***	0.01–0.06	0.00	***	0.00–0.02	0.00	***	0.00–0.02	0.00	***	0.00–0.02	0.00	***	0.00–0.04
Age (ref: 35–44)	18–24	2.97	**	1.21–7.29	0.00	***	0.00–0.00	0.00	***	0.00–0.00	0.00	***	0.00–0.00	0.00	***	0.00–0.00
	22–25	1.80	***	1.24–2.60	1.54		0.66–3.61	1.27		0.48–3.37	1.46		0.53–4.07	2.12		0.65–6.88
	45–64	0.47	***	0.34–0.66	0.51		0.23–1.16	0.40	**	0.17–0.96	0.42	*	0.16–1.11	0.60		0.18–1.93
	65+	0.12	***	0.08–0.20	0.18	***	0.06–0.57	0.16	***	0.05–0.52	0.18	***	0.05–0.63	0.24	*	0.05–1.09
Race (ref: White)	Asian	1.89		0.69–5.20	0.00	***	0.00–0.00	0.00	***	0.00–0.00	0.00	***	0.00–0.00	0.00	***	0.00–0.00
	Black	0.69		0.36–1.31	0.80		0.19–3.31	1.00		0.23–4.30	1.01		0.24–4.30	0.61		0.09–3.91
	Other races	1.47		0.76–2.85	1.24		0.21–7.45	0.00	***	0.00–0.00	0.00	***	0.00–0.00	0.00	***	0.00–0.00
Hispanic (ref: Non-Hispanic)	Hispanic	1.46		0.93–2.30	0.73		0.19–2.80	0.46		0.08–2.68	0.50		0.08–2.95	0.52		0.08–3.31
Education (ref: Less than 12 years)	High school degree	1.38		0.75–2.54	1.55		0.39–6.10	1.55		0.38–6.26	1.53		0.37–6.26	1.21		0.27–5.37
	Associate degree	1.87	**	1.02–3.43	1.22		0.33–4.52	0.88		0.23–3.39	0.91		0.23–3.55	0.81		0.20–3.24
	College degree or higher	1.34		0.70–2.57	0.73		0.18–3.01	0.61		0.14–2.74	0.51		0.10–2.49	0.35		0.07–1.79
Family income (ref: $0–$19,999)	$20,000–$39,999	0.67	*	0.44–1.03	1.58		0.54–4.57	1.24		0.39–3.96	1.16		0.34–3.93	1.06		0.28–4.09
	$40,000–$74,999	0.66	*	0.43–1.02	1.12		0.37–3.37	0.89		0.27–2.97	0.95		0.28–3.22	1.04		0.30–3.58
	$75,000 or more	0.84		0.54–1.31	0.86		0.25–3.03	0.70		0.17–2.82	0.73		0.17–3.13	0.76		0.19–2.94

Marital status (ref: Married-Spouse Present)	Never Married	1.54	**	1.06–2.25	1.48		0.62–3.57	1.17		0.41–3.37	1.33		0.46–3.88	1.80		0.66–4.96
	Married-Spouse Absent or Widowed/Divorced/ Separated	1.34	*	0.97–1.85	1.53		0.71–3.32	1.31		0.57–3.02	1.36		0.56–3.33	1.21		0.48–3.08
Employment status (ref: Employed)	Not in labor force or unemployed	1.17		0.77–1.76	1.36		0.42–4.37	2.97		0.38–23.14	2.87		0.37–22.11	2.62		0.34–20.38
Metropolitan status (ref: Non-MSA)	MSA	1.37	*	0.96–1.95	1.27		0.62–2.60	1.16		0.53–2.53	1.14		0.50–2.59	1.00		0.41–2.45
Indoor workers (ref: No)	Yes	0.83		0.57–1.21	1.19		0.38–3.70	2.76		0.37–20.57	2.36		0.32–17.46	1.81		0.24–13.43
Smokeless tobacco use § (ref: No)	Yes	3.91	***	2.53–6.05	0.00	***	0.00–0.00	0.00	***	0.00–0.00	0.00	***	0.00–0.00	0.00	***	0.00–0.00
State level cigarette tax ranking † (ref: Lowest quarter)	Med-low quarter	0.83		0.50–1.37	0.51		0.14–1.85	0.46		0.12–1.80	0.46		0.11–1.98	0.38		0.07–1.92
	Med-high quarter	0.99		0.61–1.60	0.76		0.22–2.70	0.72		0.19–2.79	0.72		0.17–3.04	0.82		0.18–3.82
	Highest quarter	1.02		0.57–1.82	1.00		0.23–4.31	0.85		0.19–3.84	0.78		0.16–3.85	0.49		0.09–2.80
Tobacco control spending per capita † (ref: Lowest quarter)	Med-low quarter	1.26		0.86–1.85	1.65		0.74–3.68	1.46		0.59–3.60	1.31		0.52–3.29	1.20		0.43–3.34
	Med-high quarter	1.19		0.78–1.82	1.45		0.49–4.29	1.90		0.55–6.53	1.51		0.41–5.59	1.16		0.24–5.56
	Highest quarter	1.06		0.65–1.73	1.09		0.32–3.71	1.37		0.33–5.61	1.09		0.22–5.32	1.07		0.19–6.09
State level worksite smoking ban (ref: Not highest level)	Highest level	0.90		0.60–1.36	1.35		0.48–3.85	1.65		0.53–5.07	1.86		0.57–6.08	2.56		0.79–8.29
State level worksite e-cigarette ban (ref: Not highest level)	Highest level	1.97	**	1.08–3.60	0.91		0.17–4.84	0.64		0.07–5.54	0.67		0.07–6.24	0.94		0.10–8.68
State level e-cigarette tax (ref: No)	Yes	0.71		0.25–2.00	0.98		0.12–7.99	0.95		0.06–14.54	2.91		0.14–61.49	5.28		0.22–128.04
Survey time (ref: July 2014)	Jan 2015	1.17		0.86–1.60	1.03		0.52–2.06	1.36		0.63–2.91	1.47		0.64–3.39	1.20		0.49–2.96
	May 2015	1.31	*	0.96–1.78	1.18		0.58–2.43	1.37		0.60–3.13	1.58		0.66–3.82	1.40		0.55–3.57
Census division (ref: Pacific)	East North Central	1.56		0.86–2.83	1.36		0.26–7.19	1.32		0.21–8.19	1.39		0.21–9.31	0.84		0.12–5.74
	East South Central	1.80	*	0.91–3.54	1.78		0.30–10.60	1.57		0.26–9.45	1.69		0.26–11.08	0.46		0.04–5.96
	Middle Atlantic	0.85		0.44–1.61	0.34		0.04–2.72	0.29		0.03–2.33	0.33		0.04–3.02	0.12		0.01–1.95
	Mountain	1.46		0.80–2.66	2.54		0.52–12.45	1.55		0.27–8.82	1.65		0.27–10.22	1.08		0.18–6.43
	New England	0.77		0.39–1.55	1.13		0.20–6.37	0.95		0.13–6.96	0.78		0.09–7.01	0.37		0.03–4.71
	South Atlantic	1.06		0.62–1.82	1.80		0.43–7.60	0.95		0.22–4.15	1.06		0.23–4.96	0.97		0.22–4.37
	West North Central	1.45		0.74–2.84	0.83		0.13–5.24	0.34		0.04–2.77	0.14		0.01–1.89	0.09	*	0.01–1.22
	West South Central	1.63		0.90–2.96	2.07		0.38–11.33	1.97		0.33–11.85	1.93		0.28–13.25	1.38		0.20–9.79

Δ Odds ratio and significance level of *p*-value: "***" < 0.01; 0.01 < "**" < 0.05; 0.05 < "*" <=0.1; § Smokeless tobacco use and e-cigarette use were using the same measure in each model; † Data for state level cigarette tax ranking are in Jan 2015 and tobacco control spending per capita are in 2015.

Supplementary Table S5a. Logistic Regression Equations, Male Never Smokers 12 Months Ago, Ever, Current (at Least Once in the Last 30 Days), 5 or More, 10 or More, 20 or More of the Last 30 Days Measures of E-cigarette Use, TUS-CPS, 2014/5

Variable	Category	Ever use			Current use			>5 days			>10 days			>20 days		
		OR	ProbΔ	95% CI	OR	Prob	95% CI	OR	Prob	95% CI	OR	Prob	95% CI	OR	Prob	95% CI
Intercept		0.01	***	0.00–0.01	0.00	***	0.00–0.01	0.00	***	0.00–0.00	0.00	***	0.00–0.00	0.00	***	0.00–0.00
Age (ref: 35–44)	18–24	3.99	***	3.03–5.27	3.97	***	2.01–7.81	3.42	***	1.47–7.93	2.37	*	0.98–5.75	2.10		0.68–6.49
	22–25	2.22	***	1.74–2.83	2.27	***	1.28–4.03	2.24	**	1.15–4.38	2.14	**	1.02–4.46	1.88		0.77–4.58
	45–64	0.42	***	0.32–0.56	0.43	***	0.23–0.80	0.40	**	0.19–0.82	0.38	**	0.17–0.86	0.14	***	0.04–0.44
	65+	0.22	***	0.14–0.36	0.35	**	0.13–0.95	0.45		0.14–1.44	0.16	**	0.03–0.80	0.17	**	0.03–0.90
Race (ref: White)	Asian	0.62	**	0.40–0.95	0.46		0.16–1.27	0.00	***	0.00–0.00	0.00	***	0.00–0.00	0.00	***	0.00–0.00
	Black	0.49	***	0.35–0.69	0.36	***	0.18–0.72	0.33	***	0.14–0.76	0.34	**	0.13–0.91	0.24	**	0.06–0.86
	Other races	1.49	**	1.03–2.17	1.16		0.40–3.37	1.16		0.32–4.26	0.06	***	0.01–0.33	0.07	***	0.01–0.52
Hispanic (ref: Non–Hispanic)	Hispanic	0.75	**	0.58–0.98	0.61	*	0.34–1.10	0.41	**	0.19–0.89	0.38	**	0.15–0.95	0.41		0.14–1.20
Education (ref: Less than 12 years)	High school degree	1.37		0.92–2.05	2.23	*	0.99–5.03	2.83	*	0.99–8.11	1.85		0.62–5.54	2.44		0.62–9.61
	Associate degree	1.66	**	1.11–2.48	2.16	*	0.97–4.81	2.65	*	0.92–7.61	1.92		0.64–5.76	1.96		0.48–8.01
	College degree or higher	1.18		0.77–1.80	0.96		0.40–2.32	1.16		0.37–3.66	0.82		0.24–2.78	1.29		0.28–6.03
Family income (ref: $0–$19,999)	$20,000–$39,999	1.03		0.78–1.36	0.74		0.39–1.39	0.79		0.34–1.82	1.23		0.48–3.19	1.26		0.41–3.84
	$40,000–$74,999	0.85		0.64–1.11	0.68		0.38–1.21	0.84		0.39–1.80	1.04		0.42–2.62	0.95		0.34–2.65
	$75,000 or more	0.90		0.68–1.20	1.05		0.58–1.90	1.38		0.64–2.98	1.90		0.76–4.75	1.78		0.65–4.90
Marital status (ref: Married-Spouse Present)	Never Married	2.39	***	1.92–2.98	1.58	*	0.95–2.62	1.51		0.80–2.87	1.68	*	0.93–3.03	2.07	*	0.97–4.40
	Married-Spouse Absent or Widowed/Divorced/Separated	1.57	***	1.16–2.13	1.71	*	0.95–3.10	1.41		0.73–2.73	1.24		0.56–2.73	1.82		0.68–4.89
Employment status (ref: Employed)	Not in labor force or unemployed	0.88		0.68–1.16	0.76		0.42–1.39	0.55		0.26–1.14	0.77		0.36–1.66	0.96		0.37–2.48
Metropolitan status (ref: Non-MSA)	MSA	1.31	**	1.03–1.67	1.21		0.70–2.09	1.27		0.69–2.33	1.17		0.61–2.26	1.55		0.58–4.14
Indoor workers (ref: No)	Yes	1.02		0.83–1.25	1.00		0.65–1.53	0.87		0.54–1.40	0.90		0.53–1.52	0.82		0.40–1.65
Current smoking status (ref: Never smokers one year ago)	Current Smokers	22.51	***	16.64–30.45	26.98	***	17.53–41.52	31.82	***	19.52–51.87	36.65	***	21.74–61.76	35.74	***	18.42–69.36
Smokeless tobacco use § (ref: No)	Yes	6.74	***	5.54–8.21	1.80		0.88–3.70	1.88		0.84–4.20	2.32	*	0.97–5.57	0.77		0.09–6.78
State level cigarette tax ranking † (ref: Lowest quarter)	Med-low quarter	1.38	**	1.02–1.88	1.57		0.81–3.02	2.89	***	1.33–6.29	1.96		0.87–4.45	1.81		0.69–4.73
	Med-high quarter	1.32	*	0.96–1.79	1.35		0.68–2.66	2.27	*	0.99–5.22	1.49		0.62–3.58	1.22		0.42–3.57
	Highest quarter	2.04	***	1.40–2.98	1.02		0.44–2.37	2.21	*	0.89–5.49	2.15		0.80–5.80	1.19		0.38–3.72
	Med-low quarter	1.31	*	1.00–1.71	1.45		0.83–2.55	1.93	**	1.06–3.50	1.93	*	0.98–3.82	1.70		0.78–3.70

Tobacco control spending per capita † (ref: Lowest quarter)	Med-high quarter	1.00		0.77–1.30	0.79		0.45–1.37	1.03		0.54–1.97	1.40		0.66–2.94	1.18		0.43–3.23
	Highest quarter	0.77	*	0.57–1.04	0.57	*	0.30–1.10	0.69		0.31–1.51	0.92		0.37–2.25	0.83		0.25–2.78
State level worksite smoking ban (ref: Not highest level)	Highest level	0.77	**	0.60–0.99	0.94		0.56–1.58	0.62		0.33–1.17	0.70		0.36–1.38	1.04		0.50–2.17
State level worksite e-cigarette ban (ref: Not highest level)	Highest level	0.67	*	0.42–1.06	1.77		0.66–4.77	1.74		0.56–5.37	1.89		0.50–7.14	3.41	*	0.80–14.45
State level e-cigarette tax (ref: No)	Yes	0.50	*	0.23–1.09	0.73		0.12–4.40	0.37		0.03–4.03	0.21		0.02–2.44	0.73		0.05–10.64
Survey time (ref: July 2014)	Jan 2015	1.04		0.85–1.27	0.97		0.62–1.51	0.83		0.48–1.44	0.76		0.41–1.42	0.64		0.30–1.39
	May 2015	1.03		0.84–1.26	0.72		0.47–1.11	0.87		0.52–1.43	1.02		0.58–1.80	0.89		0.44–1.81
Census division (ref: Pacific)	East North Central	0.69	*	0.47–1.00	0.74		0.31–1.74	1.14		0.40–3.23	1.03		0.34–3.06	1.14		0.29–4.45
	East South Central	0.53	***	0.34–0.82	0.65		0.27–1.58	1.37		0.53–3.53	1.12		0.39–3.23	1.61		0.45–5.68
	Middle Atlantic	0.76		0.51–1.14	0.45		0.17–1.22	0.41		0.10–1.72	0.18	**	0.04–0.86	0.26		0.04–1.54
	Mountain	1.36	*	0.95–1.94	0.79		0.35–1.78	1.69		0.61–4.70	1.63		0.52–5.11	0.95		0.23–4.01
	New England	0.38	***	0.23–0.64	0.46		0.14–1.49	0.65		0.16–2.69	0.64		0.14–2.96	1.30		0.23–7.29
	South Atlantic	0.75	*	0.54–1.04	0.78		0.39–1.57	1.48		0.62–3.54	1.22		0.47–3.17	1.42		0.45–4.51
	West North Central	0.83		0.56–1.23	0.48		0.19–1.24	0.83		0.25–2.82	1.05		0.30–3.62	0.88		0.18–4.44
	West South Central	0.83		0.57–1.21	1.05		0.48–2.27	1.24		0.48–3.22	1.22		0.43–3.41	0.98		0.23–4.08

Δ Odds ratio and significance level of *p*-value: "***" < 0.01; 0.01 < "**" < 0.05; 0.05 < "*" <=0.1; § Smokeless tobacco use and e-cigarette use were using the same measure in each model; † Data for state level cigarette tax ranking are in Jan 2015 and tobacco control spending per capita are in 2015.

Supplementary Table S5b. Logistic Regression Equations, Female Never Smokers 12 Months Ago, Ever, Current (at Least Once in the Last 30 Days), 5 or More, 10 or More, 20 or More of the Last 30 Days Measures of E-cigarette Use, TUS-CPS, 2014/5

Variable	Category	Ever use			Current use			>5 days			>10 days			>20 days		
		OR	ProbΔ	95% CI	OR	Prob	95% CI	OR	Prob	95% CI	OR	Prob	95% CI	OR	Prob	95% CI
Intercept		0.01	***	0.00–0.02	0.00	***	0.00–0.01	0.00	***	0.00–0.01	0.00	***	0.00–0.01	0.00	***	0.00–0.00
Age (ref: 35–44)	18–24	3.06	***	2.27–4.12	1.91	*	0.96–3.80	1.65		0.73–3.73	2.26		0.85–6.03	2.10		0.62–7.12
	22–25	1.96	***	1.50–2.55	1.57		0.84–2.91	0.99		0.48–2.06	1.37		0.58–3.21	1.91		0.68–5.37
	45–64	0.46	***	0.35–0.61	0.41	***	0.21–0.80	0.30	***	0.14–0.63	0.29	***	0.12–0.73	0.35	*	0.12–1.02
	65+	0.13	***	0.07–0.23	0.13	***	0.04–0.38	0.10	***	0.03–0.31	0.14	***	0.04–0.50	0.16	**	0.03–0.76
Race (ref: White)	Asian	0.44	***	0.23–0.81	0.04	***	0.01–0.16	0.03	***	0.00–0.23	0.00	***	0.00–0.00	0.00	***	0.00–0.00
	Black	0.38	***	0.27–0.54	0.24	***	0.11–0.51	0.28	***	0.12–0.67	0.28	**	0.10–0.74	0.26	**	0.08–0.89
	Other races	1.35		0.87–2.08	1.20		0.49–2.92	0.44	*	0.17–1.11	0.27	*	0.07–1.08	0.12	**	0.02–0.90
Hispanic (ref: Non-Hispanic)	Hispanic	0.54	***	0.40–0.73	0.40	***	0.20–0.79	0.25	***	0.10–0.59	0.31	**	0.12–0.81	0.24	**	0.07–0.86
Education (ref: Less than 12 years)	High school degree	1.17		0.74–1.84	1.70		0.81–3.60	1.73		0.75–3.97	1.84		0.75–4.48	2.41		0.78–7.44
	Associate degree	1.70	**	1.10–2.63	1.54		0.73–3.23	1.31		0.56–3.06	1.34		0.53–3.36	1.44		0.45–4.65
	College degree or higher	0.89		0.56–1.42	0.67		0.27–1.64	0.60		0.21–1.77	0.62		0.18–2.08	0.06	***	0.01–0.36
Family income (ref: $0–$19,999)	$20,000–$39,999	0.80		0.60–1.06	0.83		0.46–1.51	0.76		0.38–1.53	0.52		0.23–1.17	1.07		0.41–2.83
	$40,000–$74,999	0.75	*	0.56–1.01	0.61		0.32–1.14	0.61		0.29–1.29	0.42	*	0.17–1.00	1.13		0.42–3.02
	$75,000 or more	0.84		0.61–1.16	0.80		0.40–1.61	0.78		0.35–1.73	0.58		0.23–1.46	1.04		0.37–2.96
Marital status (ref: Married-Spouse Present)	Never Married	1.69	***	1.33–2.14	2.30	***	1.30–4.08	2.04	**	1.05–3.96	1.32		0.61–2.88	1.75		0.56–5.47
	Married-Spouse Absent or Widowed/Divorced/Separated	1.37	**	1.04–1.80	2.18	***	1.22–3.91	2.53	***	1.29–4.98	2.62	**	1.25–5.47	4.34	***	1.69–11.11
Employment status (ref: Employed)	Not in labor force or unemployed	0.98		0.70–1.37	0.57	*	0.31–1.06	0.40	***	0.20–0.80	0.28	***	0.13–0.60	0.26	***	0.10–0.66
Metropolitan status (ref: Non-MSA)	MSA	1.16		0.89–1.52	0.85		0.49–1.50	0.86		0.45–1.67	0.96		0.43–2.13	2.29		0.83–6.35
Indoor workers (ref: No)	Yes	1.25		0.92–1.71	0.70		0.38–1.28	0.50	**	0.25–0.97	0.42	**	0.20–0.88	0.34	**	0.14–0.81
Current smoking status (ref: Never smokers one year ago)	Current Smokers	39.31	***	30.07–51.39	48.29	***	30.91–75.44	60.50	***	35.95–101.82	61.66	***	32.72–116.20	44.82	***	20.22–99.34

Smokeless tobacco use § (ref: No)	Yes	14.92	***	9.59–23.21	0.00	***	0.00–0.00	0.00	***	0.00–0.00	0.00	***	0.00–0.00	0.00	***	0.00–0.00
State level cigarette tax ranking† (ref: Lowest quarter)	Med-low quarter	0.87		0.61–1.25	0.83		0.39–1.75	0.67		0.30–1.52	0.88		0.35–2.24	1.09		0.36–3.33
	Med-high quarter	0.96		0.67–1.38	1.38		0.67–2.84	1.14		0.51–2.56	0.97		0.38–2.45	1.72		0.49–5.94
	Highest quarter	1.40		0.90–2.17	1.40		0.59–3.35	1.06		0.39–2.89	1.18		0.36–3.88	1.53		0.38–6.21
Tobacco control spending per capita† (ref: Lowest quarter)	Med-low quarter	1.16		0.86–1.55	1.66		0.86–3.21	1.52		0.69–3.35	2.26	*	0.88–5.79	6.53	***	2.17–19.62
	Med-high quarter	1.00		0.74–1.35	1.34		0.71–2.54	1.12		0.54–2.32	1.57		0.67–3.66	3.38	**	1.09–10.50
	Highest quarter	0.98		0.70–1.38	1.58		0.79–3.16	1.16		0.50–2.68	1.28		0.42–3.86	2.98		0.68–13.17
State level worksite smoking ban (ref: Not highest level)	Highest level	0.82		0.62–1.08	0.81		0.45–1.46	1.23		0.64–2.37	1.01		0.47–2.17	0.50		0.20–1.27
State level worksite e–cigarette ban (ref: Not highest level)	Highest level	0.60	*	0.34–1.06	0.60		0.20–1.76	0.55		0.15–2.03	0.48		0.10–2.18	0.34		0.04–3.15
State level e-cigarette tax (ref: No)	Yes	0.56		0.23–1.33	0.75		0.14–4.06	1.12		0.18–6.87	1.27		0.17–9.71	2.53		0.22–29.53
Survey time (ref: July 2014)	Jan 2015	1.16		0.93–1.46	0.79		0.48–1.31	0.67		0.37–1.22	0.95		0.47–1.93	0.50		0.22–1.17
	May 2015	1.03		0.81–1.30	1.19		0.74–1.93	1.30		0.75–2.27	1.90	*	0.97–3.71	1.54		0.66–3.58
Census division (ref: Pacific)	East North Central	0.82		0.53–1.28	0.97		0.33–2.86	0.65		0.18–2.35	0.94		0.22–4.04	0.67		0.11–4.09
	East South Central	0.65	*	0.41–1.03	1.23		0.43–3.52	0.87		0.25–2.95	1.54		0.43–5.47	1.31		0.24–7.18
	Middle Atlantic	0.63	*	0.38–1.04	0.91		0.31–2.70	0.53		0.15–1.89	1.20		0.30–4.81	0.43		0.06–3.04
	Mountain	1.25		0.82–1.91	2.14		0.75–6.07	1.68		0.54–5.22	2.60		0.60–11.21	3.74		0.68-20.59
	New England	0.64		0.37–1.10	1.06		0.33–3.37	0.78		0.19–3.24	1.08		0.19–6.21	0.64		0.05–7.91
	South Atlantic	0.62	**	0.42–0.92	2.08	*	0.90–4.78	1.12		0.45–2.83	1.48		0.51–4.31	1.42		0.37–5.52
	West North Central	0.85		0.53–1.36	1.86		0.68–5.11	1.30		0.42–4.04	1.93		0.52–7.21	1.55		0.24–10.01
	West South Central	0.96		0.63–1.47	1.45		0.57–3.68	1.07		0.36–3.18	1.76		0.49–6.34	1.26		0.27–5.87

Δ Odds ratio and significance level of *p*-value: "***" < 0.01; 0.01 < "**" < 0.05; 0.05 < "*" <=0.1; § Smokeless tobacco use and e–cigarette use were using the same measure in each model; † Data for state level cigarette tax ranking are in Jan 2015 and tobacco control spending per capita are in 2015.
